# Visible-light-promoted radical cyclisation of unactivated alkenes in benzimidazoles: synthesis of difluoromethyl- and aryldifluoromethyl-substituted polycyclic imidazoles

**DOI:** 10.3762/bjoc.21.15

**Published:** 2025-01-30

**Authors:** Yujun Pang, Jinglan Yan, Nawaf Al-Maharik, Qian Zhang, Zeguo Fang, Dong Li

**Affiliations:** 1 New Materials and Green Manufacturing Talent Introduction and Innovation Demonstration Base, Hubei University of Technology, 430068 Wuhan, China; 2 Department of chemistry, Faculty of Science, An Najah National University, Nablus, Palestine

**Keywords:** cyclization, difluoromethylation, hypervalent iodine, polycyclic imidazole, visible light

## Abstract

An efficient and eco-friendly approach for synthesizing difluoromethyl- and aryldifluoromethyl-substituted polycyclic imidazoles was established via a visible-light-promoted radical cyclization reaction. This method employed the readily accessible and inexpensive CF_2_HCO_2_H or PhCF_2_COOH, along with benzimidazoles bearing unactivated alkenes and PhI(OAc)_2_ as substrates, and proceeded without the need of any base, metal catalyst, photocatalyst or additive. In total, 24 examples were examined, and all of them successfully underwent cyclization reaction to produce the target products in good to excellent yields. Mechanistic studies revealed that the reaction proceeds via a radical pathway.

## Introduction

**Figure 1:**
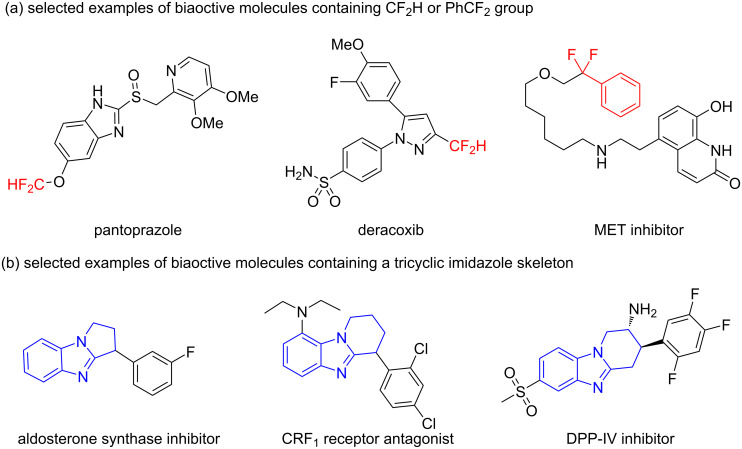
Selected examples containing tricyclic imidazole, CF_2_H or PhCF_2_ group.

Organofluorine compounds continue to play important roles in pharmaceuticals and agrochemicals nowadays, largely due to the unique ability of fluorinated groups to influence the physicochemical and biochemical properties of molecules [1–3]. Among the various fluorinated functionalities, the difluoromethyl (CF_2_H) group and its aryl-substituted derivative, the benzylic difluoromethylene (PhCF_2_) group, stand out as particularly valuable in drug design. The CF_2_H group can serve as a lipophilic isostere for hydroxy (OH), amino (NH_2_), and thiol (SH) groups, thereby enhancing the efficacy and selectivity of therapeutic agents [4–6]. Similarly, the PhCF_2_ group offers unique properties that can modify the activity and pharmacokinetic profiles of drugs [7]. Prominent examples include pantoprazole, a widely used proton-pump inhibitor (PPI) featuring a CF_2_H group; deracoxib, another drug that also incorporates a CF_2_H moiety in its structure; and a MET inhibitor specifically designed with a PhCF_2_ group (Figure 1a) [8–10]. As a result, there is a pressing need for the development of efficient methods for incorporating both the CF_2_H and PhCF_2_ groups into diverse molecular frameworks, particularly those with bioactivity properties.

**Scheme 1:**
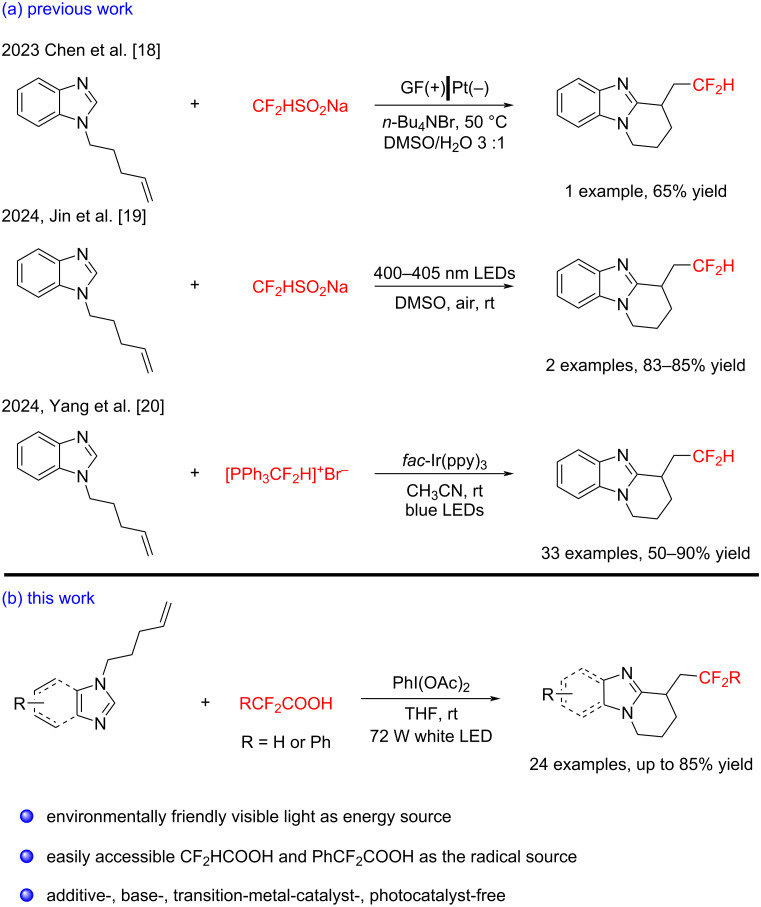
Strategies for the synthesis of difluoromethylated and difluoroarylmethylated tricyclic imidazoles.

The benzimidazole core is widely recognized as a vital pharmacophore in medicinal chemistry due to its special biological activity [11–13]. In particular, the tricyclic benzimidazole skeleton is ubiquitous in many bioactive compounds and therapeutic agents (Figure 1b) [14–16]. Recent studies have shown that fluorinated benzimidazole derivatives exhibit improved pharmacokinetic properties [17], which has further sparked interest in their development. Consequently, constructing benzimidazoles bearing the CF_2_H and PhCF_2_ groups has garnered significant attention. However, despite this growing interest, only a limited number of research groups have reported the direct difluoromethylation/cyclization reaction of benzimidazoles with alkenes for the syntheses of difluoromethylated tricyclic benzimidazoles to date. For example, in 2023, Chen and co-workers pioneered an electrochemical approach for the difluoromethylation and cyclization reaction of unactivated alkenes within benzimidazole molecules using CF_2_HSO_2_Na [18]. Subsequently, in 2024, Jin [19] and Yang [20] developed visible light-induced difluoromethylation strategies for unactivated alkenes within benzimidazoles using different CF_2_H sources (CF_2_HSO_2_Na and ([Ph_3_PCF_2_H]^+^Br^−^), respectively (Scheme 1a). Despite these advances, the above methods still suffer from several limitations, including a narrow substrate scope, the reliance on expensive metal catalysts and excess additives, and the need for multistep synthesis of difluoromethylating reagents. These drawbacks restrict their broader applicability in drug design to some extent. Besides, the incorporation of the PhCF_2_ group into tricyclic imidazoles has never been reported according to our best knowledge. Therefore, it is essential to explore environmentally friendly, cost-effective synthetic approaches for the construction of both difluoromethylated and aryldifluoromethylated benzimidazoles.

Inspired by previous work in radical chemistry, we turned our attention to difluoroacetic acid (CF_2_HCOOH) and α,α-difluorobenzeneacetic acid (PhCF_2_COOH), both of which are inexpensive and readily available industrial raw materials. In 2019, Gouverneur and co-workers reported a hydrodifluoromethylation of unactivated alkenes, wherein a CF_2_H radical was generated from CF_2_HCOOH using (diacetoxyiodo)benzene (PIDA) and light [21]. This CF_2_H radical then added to the double bond to form a new alkyl radical, which underwent hydrogen atom abstraction to yield the hydrodifluoromethylation product. Building upon this work, we hypothesized that if the newly formed alkyl radical could undergo intramolecular cyclization with an aromatic ring, instead of hydrogen abstraction, it could enable the construction of polycyclic structures. Thus, as part of our ongoing interest in radical cyclization reactions [22–26], we report here a sustainable and efficient protocol for synthesizing difluoromethylated and aryldifluoromethylated polycyclic imidazoles via visible-light-promoted cyclization of unactivated alkene-containing imidazoles with CF_2_HCOOH or PhCF_2_COOH, and PIDA under additive-, base-, and metal catalyst-free conditions (Scheme 1b).

## Results and Discussion

Initially, 1-(pent-4-en-1-yl)-1*H*-benzo[*d*]imidazole (**1a**), CF_2_HCOOH, and PIDA were chosen as the template substrates for this radical difluoromethylation and cyclization reaction (Table 1). Employing PIDA as the promoter, THF as the solvent, and 72 W white LED as the light source, the desired product **3a** formed in 85% isolated yield at room temperature (Table 1, entry 1). We found that the hypervalent iodine reagent was of significant importance for the present transformation (Table 1, entries 2 and 3), and PIDA was the most efficient promoter. Changing THF to other solvents, such as DCM, EtOH, DMF, CH_3_CN, EtOAc, or DMSO, resulted in a lower yield (Table 1, entries 4–9). Furthermore, variations in the amounts of PIDA or CF_2_HCOOH led to diminished yields (Table 1, entries 10–13), and conducting the reaction under air instead of nitrogen significantly lowered the yield (Table 1, entry 14). Control experiments showed that the absence of PIDA resulted in no reaction (Table 1, entry 15), while the use of a 40 W light source or the absence of visible light also reduced the product yield (Table 1, entries 16 and 17).

**Table 1:** Optimization of reaction conditions.^a^

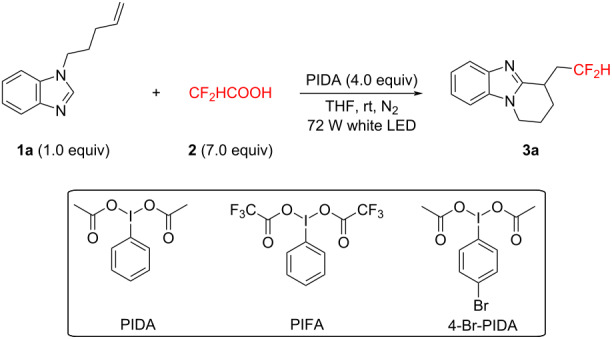		

Entry	Variation from the standard conditions	Yield (%)^b^

1	none	85
2	PIFA	37
3	4-Br-PIDA	trace
4	DCM instead of THF	trace
5	EtOH instead of THF	NR
6	DMF instead of THF	13
7	CH_3_CN instead of THF	17
8	EtOAc instead of THF	12
9	DMSO instead of THF	12
10	PIDA (3.0 equiv) instead of PIDA (4.0 equiv)	62
11	PIDA (5.0 equiv) instead of PIDA (4.0 equiv)	80
12	**2** (6 equiv) instead of **2** (7.0 equiv)	78
13	**2** (8 equiv) instead of **2** (7.0 equiv)	83
14	air instead of N_2_	55
15	without PIDA	NR
16	40 W white LED instead of 72 W white LED	42
17	dark	40

^a^Reaction conditions: **1a** (0.2 mmol), **2** (1.4 mmol), and PIDA (0.8 mmol) in solvent (2 mL) irradiated with 72 W white LEDs at room temperature for 12 h under a N_2_ atmosphere. NR no reaction. ^b^Isolated yield.

**Scheme 2:**
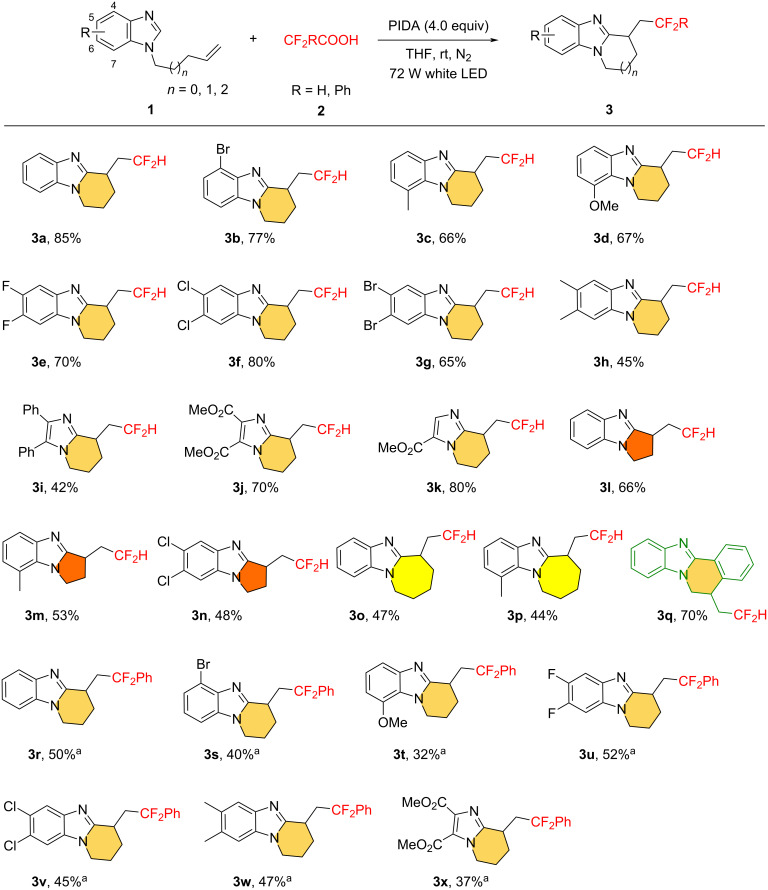
Substrate scope of the protocol. Reaction conditions: **1** (0.2 mmol), **2** (1.4 mmol), and PIDA (0.8 mmol) in solvent (2 mL) irradiated with 72 W white LEDs at room temperature for 12 h under a N_2_ atmosphere. Yields refer to isolated yield. ^a^α,α-Difluorobenzeneacetic acid (2 equiv) was used.

**Scheme 3:**
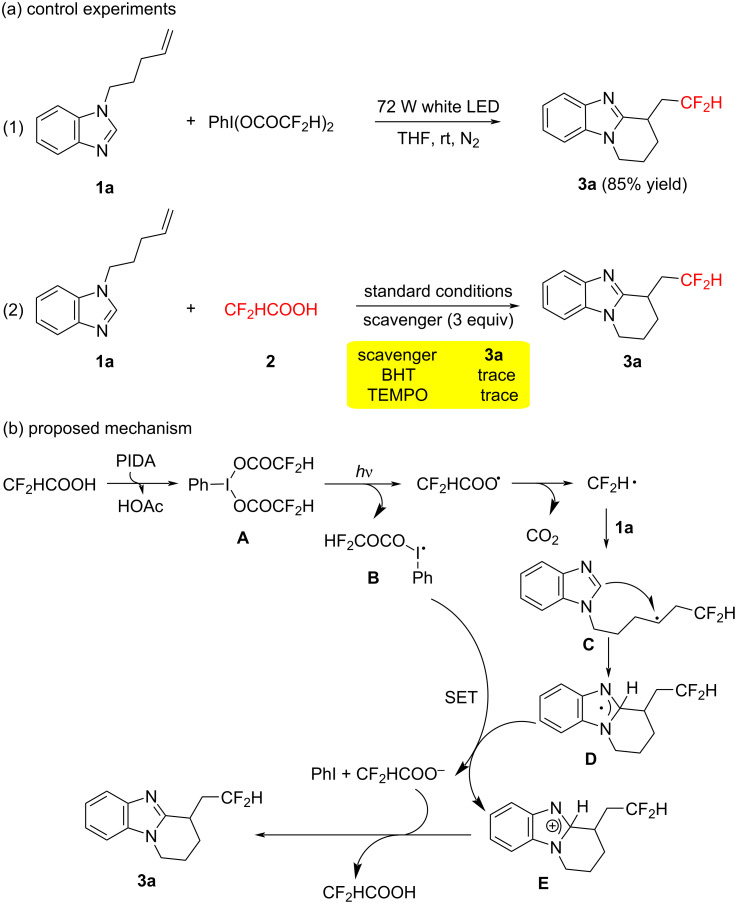
Control experiments and plausible mechanism.

With the optimized conditions in hand (Table 1, entry 1), the generality of the visible-light-promoted radical difluoromethlation/cyclization reaction was first investigated (Scheme 2). We were delighted to observe that the benzimidazole ring exhibited good tolerance for both electron-withdrawing groups such as fluorine (–F), bromine (–Br), and chlorine (–Cl), as well as electron-donating substituents like methoxy (–OMe) and methyl (–Me), yielding the corresponding 6-membered tricyclic imidazoles in moderate to good yields (**3b**–**h**). Benzene rings substituted with halogen atoms (–F, –Cl, –Br) were also suitable for this transformation, efficiently giving the desired products in yields of 65–80% (**3b**, **3e**–**g**), thus facilitating further functionalization possibilities. Notably, substrates with substituents at the sterically hindered 7-position of the benzimidazole ring also successfully underwent smooth cyclization, leading to the formation of products **3c** and **3d**. Furthermore, the methodology was compatible with 5,6-disubstituted *N*-alkenylbenzimidazoles, including those with -difluoro, -dichloro, -dibromo, and -dimethyl substitutions, resulting in the production of the anticipated products in yields ranging from moderate to good (**3e**–**h**). Afterwards, we shifted our focus to substrates containing a single imidazole ring and discovered that the radical difluoromethylation and subsequent cyclization of unactivated olefin-containing imidazoles proceeded efficiently, generating the CF_2_H-substituted bicyclic imidazoles with yields ranging from moderate to high (specifically, **3i** yielded 42%, **3j** yielded 70%, and **3k** yielded 80%). The relatively lower yield of **3i** can be attributed to the formation of side products due to the presence of the phenyl ring. Furthermore, terminal olefins with varying chain lengths also reacted successfully, resulting in 5-membered and 7-membered cyclized products (**3l**–**p**) with yields between 44% and 66%. The lower yields in these cases might be due to the low reactivity of the intermediate **C** (Scheme 3), which may have made it less likely to undergo the desired transformation. To broaden application of this strategy, we tested other substrates as well. For instance, we successfully converted the *N*-alkenyl 2-arylbenzimidazole substrate into the desired product (**3q**). Finally, we examined the substrates for the radical aryldifluoromethylation/cyclization reaction (for details about optimization conditions, please see Supporting Information File 1). We were delighted to find that when 2-fluorophenylacetic acid was employed as the fluorine source, a wide range of benzimidazole substrates were also compatible with this reaction. For example, substrates with a bromine atom occupying the 4-position and a methoxy group at the 7-position could be successfully converted into the target products (**3s** and **3t**). In addition, doubly substituted benzimidazoles (**3u**–**w**), as well as the single imidazole (**3x**), were also found to be applicable. This demonstrates the versatility of our methodology and its potential for further exploration in diverse chemical spaces.

To gain a deeper understanding of the mechanism behind the observed reaction, we conducted a series of control experiments as outlined in Scheme 3a. Initially, we performed the model reaction with **1a** and PhI(OCOCF_2_H)_2_, which resulted in the formation of product **3a** with an 85% yield. This finding indicated that PhI(OCOCF_2_H)_2_ played a crucial role as an intermediate in the reaction. Subsequently, we introduced 3 equivalents of a radical scavenger (either TEMPO or BHT) into the reaction mixture, which significantly impeded the progress of the desired reaction. Therefore, on the basis of the above experimental results and previous reports [21,27–30], we proposed a possible reaction mechanism (Scheme 3b), taking CF_2_HCOOH as the illustrative example. Initially, a double ligand exchange between PIDA and CF_2_HCOOH would generate PhI(OCOCF_2_H)_2_ **A**. Homolysis of **A** under visible light (72 W white light) produced an iodanyl radical **B** and a CF_2_H radical. The CF_2_H radical regioselectively added to **1a** to form intermediate **C**. Subsequently, intermediate **C** could be converted into the radical intermediate **D** via intramolecular radical cyclization. A single-electron-transfer (SET) process then occurred between the radical **B** and the radical **D**, resulting in the generation of cationic intermediate **E**, difluoroacetate anion and PhI. Finally, the product **3a** was obtained after the deprotonation by difluoroacetate anion.

## Conclusion

In summary, we have successfully developed a sustainable and efficient method for synthesizing difluoromethylated and aryldifluoromethylated polycyclic imidazoles through visible-light-promoted radical reactions. In contrast to previous reports, we achieved high yields of tricyclic and bicyclic imidazoles under additive-, base-, and metal catalyst-free conditions utilizing difluoroacetic acid and α,α-difluorobenzeneacetic acid as the readily available fluorine sources. The significant advantages of this approach, including its environmental friendliness and cost-effectiveness, position it as a valuable strategy in drug design and the synthesis of fluorinated compounds.

## Supporting Information

File 1 Experimental procedures, product characterization, and copies of NMR spectra.

## Data Availability

All data that supports the findings of this study is available in the published article and/or the supporting information of this article.
