# Supplementary material for: Visible-light-promoted radical cyclisation of unactivated alkenes in benzimidazoles: synthesis of difluoromethyl- and aryldifluoromethyl-substituted polycyclic imidazoles
Source: Beilstein J Org Chem. 2025 Jan 30;21:234–41. doi: 10.3762/bjoc.21.15 (PMC11789676; doi:10.3762/bjoc.21.15)
Supplement: File 1 — Experimental procedures, product characterization, and copies of NMR spectra. [file Beilstein_J_Org_Chem-21-234-s001.pdf]

BEILSTEIN JOURNAL OF ORGANIC CHEMISTRY

Supporting Information

for

Visible-light-promoted radical cyclisation of unactivated alkenes in benzimidazoles: synthesis of difluoromethyl- and aryldifluoromethyl-substituted polycyclic imidazoles

Yujun Pang, Jinglan Yan, Nawaf Al-Maharik, Qian Zhang, Zeguo Fang and Dong Li

Beilstein J. Org. Chem. **2025,** *21,* 234–241. doi:10.3762/bjoc.21.15

Experimental procedures, product characterization, and copies of NMR spectra

License and Terms: This is a supporting information file under the terms of the Creative Commons Attribution License (https://creativecommons.org/licenses/by/4.0). Please note that the reuse, redistribution and reproduction in particular requires that the author(s) and source are credited and that individual graphics may be subject to special legal provisions.
The license is subject to the *Beilstein Journal of Organic Chemistry* terms and conditions: (https://www.beilstein-journals.org/bjoc/terms)

Table of contents

Experimental section	
Instrumentation and chemicals	S2
Preparation of substrates	S2
Screening the reaction conditions	S2
Experimental procedures	S3
Characterization data	S3–S11
Copies of ^{1}H, ^{13}C, and ^{19}F NMR spectra	S12–S59
References	S60

Experimental section

Instrumentation and chemicals

^{1}H NMR (400 MHz), ^{13}C NMR (100 MHz), and ^{19}F NMR (376 MHz) were recorded on a BrukerNMR apparatus with $CDCl_3$ as the solvent. The chemical shifts are reported in δ (ppm) values. ^{1}H NMR chemical shifts were determined relative to the internal tetramethylsilane signal at δ 0.0. ^{19}F NMR chemical shifts were determined relative to external $CFCl_3$ at δ 0.0. Data for ^{1}H, ^{13}C, and ^{19}F NMR were recorded as follows: chemical shift (δ, ppm), multiplicity (s = singlet, d = doublet, t = triplet, m = multiplet, dd = doublet of doublets, br = broad). Coupling constants (*J*) are reported in hertz (Hz). Melting points were measured by SGW X-4A microscopic apparatus. HRMS was measured by Q Exactive Hybrid Quadrupole-Orbitrap LC/MS spectrometer.

The starting materials, including the 1*H*-benzo[*d*]imidazole, sodium hydride obtained from commercial sources such as Aladdin, Macklin, Alfa Aesar, Ourchem and used as received unless otherwise noted. Ethyl acetate and petroleum ether were used for column chromatography without further purification.

Preparation of substrates

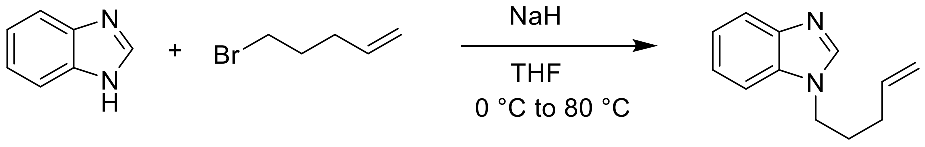

In a manner similar to reference[1]. To a dried 100 mL round bottom flask equipped with a magnetic stir bar were added 1*H*-benzo[*d*]imidazole (10 mmol) and THF (40 mL). After cooling to 0 °C, NaH (480 mg, 20 mmol) was added and stirring was continued for 15 min at room temperature. Subsequently, bromoalkene (12 mmol) was added and stirring was further continued for 6 h at 80 °C to confirm completion of the reaction by TLC analysis. To the reaction mixture were added ethyl acetate (30mL × 3) and water (30 mL) for extraction. The combined organic layers were dried over anhydrous sodium sulfate and concentrated under vacuum. Flash column chromatography on silica gel using ethyl acetate as an eluent afforded product **1**.

Screening the reaction conditions

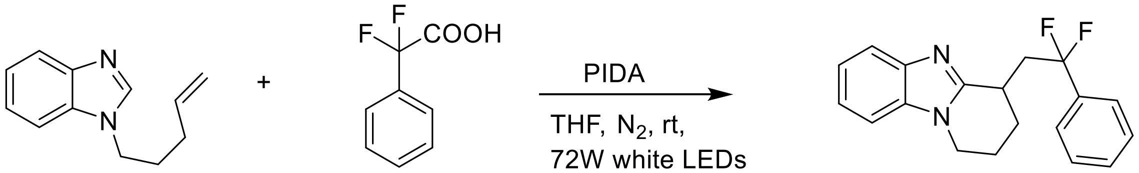

Entry	1:2	PIDA (equiv)	Time (h)	Yield (%)
1	1:7	4.0	12	21
2	1:5	4.0	12	34
3	1:2	4.0	12	50
4	1:2	2.0	12	25
5	1:1	4.0	12	45

Experimental procedures

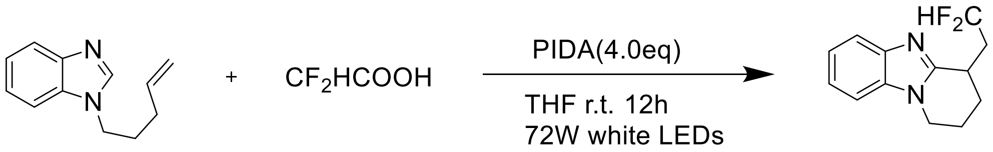

A 10 mL sealed tube was charged with **1a** (0.2 mmol, 37.2 mg), **2** (1.4 mmol, 134.4 mg), PIDA (0.8 mmol, 257.7 mg), and THF (2.0 mL). The tube was evacuated and backfilled with N_2 for 3 times. The resulting mixture was stirred and irradiated by 72 W white LEDs for 12 hours at room temperature. After the reaction was complete, the mixture was added into H_2O (25 mL) and extracted with ethyl acetate (10 mL) three times. The combined organic layer was dried over anhydrous $MgSO_4$ and filtered. After removal of the solvent in vacuo, the residue was purified by column chromatography (ethyl acetate/petroleum ether 1:4) to afford the pure product **3a** in 85% yield (40 mg, colourless oil).

Characterization data

4-(2,2-Difluoroethyl)-1,2,3,4-tetrahydrobenzo[4,5]imidazo[1,2-*a*]pyridine (3a)

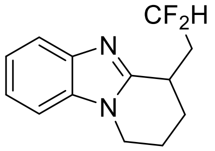

Colourless oil (40 mg, 85% yield); ^{1}H NMR (400 MHz, $CDCl_3$): δ 7.82-7.59 (m, 1H), 7.41-7.10 (m, 3H), 6.39 (tdd, *J* = 56.8, 8.0, 4.7 Hz, 1H), 4.25-4.10 (m, 1H), 4.02-3.90 (m, 1H), 3.40-3.20 (m, 1H), 2.85-2.66 (m, 1H), 2.33-2.04 (m, 4H), 1.81-1.67 (m, 1H); ^{13}C NMR (100 MHz, $CDCl_3$) δ 153.3, 142.6, 134.6, 122.3, 122.2, 119.2, 116.8 (t, *J* = 238.0 Hz), 109.0, 42.4, 38.0 (t, *J* = 22.0 Hz), 31.3 (dd, *J* = 6.0, 4.0 Hz), 27.5, 21.7; ^{19}F {H} NMR (376 MHz, $CDCl_3$) δ -113.5 (d, *J* = 282.0 Hz, 1F), -117.6 (d, *J* = 285.8 Hz, 1F); HRMS (ESI, m/z)[M + H^+]: calculated for $C_{13}H_{15}F_2N_2$: 237.1198, found 237.1186. The spectral data were in accordance with the literature[1][2].

6-Bromo-4-(2,2-difluoroethyl)-1,2,3,4-tetrahydrobenzo[4,5]imidazo[1,2-*a*]pyridine (3b)

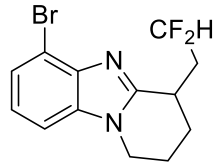

Colourless oil (44.5 mg, 77% yield); ^{1}H NMR (400 MHz, $CDCl_3$) δ 7.46 (d, *J* = 8.0 Hz, 1H), 7.29-7.25 (m, 1H), 7.13 (t, *J* = 8.0 Hz, 1H), 6.42 (tdd, *J* = 56.0, 5.3, 3.9 Hz, 1H), 4.21-4.16 (m, 1H), 4.02-3.97 (m, 1H), 3.40-3.32 (m, 1H), 2.92-2.76 (m, 1H), 2.36-2.03 (m, 4H), 1.82-1.76 (m, 1H); ^{13}C NMR (100 MHz, $CDCl_3$) δ 154.0, 141.3, 135.2, 125.4, 123.1, 116.8 (t, *J* = 238.0 Hz), 112.8, 108,4 42.9, 38.0 (t, *J* = 21.0 Hz), 31.3 (dd, *J* = 6.0, 4.0 Hz), 27.2, 21.5; ^{19}F {H} NMR (376 MHz, $CDCl_3$) δ -113.1 (d, *J* = 282.0 Hz, 1F), -117.6 (d, *J* = 282.0 Hz, 1F); HRMS (ESI, m/z)[M + H^+]: calculated for $C_{13}H_{14}BrF_2N_2$: 315.0303, found 315.0298.

4-(2,2-Difluoroethyl)-9-methyl-1,2,3,4-tetrahydrobenzo[4,5]imidazo[1,2-*a*]pyridine (3c)

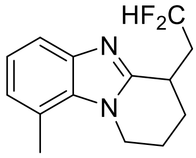

Colourless oil (33 mg, 66% yield); ^{1}H NMR (400 MHz, $CDCl_3$) δ 7.22-7.08 (m, 2H), 7.09-7.00 (m, 1H), 6.47 (tdd, *J* = 56.9, 5.8, 3.6 Hz, 1H), 4.24-4.06 (m, 1H), 4.02-3.86 (m, 1H), 3.40-3.20 (m, 1H), 2.81-2.65 (m, 1H), 2.64 (s, 3H), 2.32-1.98 (m, 4H), 1.83 -1.67 (m, 1H).; ^{13}C NMR (100 MHz, $CDCl_3$) δ 152.4, 141.8, 134.2, 129.2, 122.8, 122.1, 117.1 (t, *J* = 238.0 Hz), 106.5, 42.6, 38.3 (t, *J* = 21.0 Hz), 31.3 (dd, *J* = 7.0, 4.0 Hz), 27.5, 21.6, 16.6; ^{19}F｛H｝NMR (376 MHz, $CDCl_3$) δ -113.2 (d, *J* = 282.0 Hz, 1F), -117.7 (d, *J* = 285.8 Hz, 1F); HRMS (ESI, m/z)[M + H^+]: calculated for $C_{14}H_{17}F_2N_2$: 251.1355, found 251.1304. The spectral data were in accordance with the literature[1].

4-(2,2-Difluoroethyl)-9-methoxy-1,2,3,4-tetrahydrobenzo[4,5]imidazo[1,2-*a*]pyridine (3d)

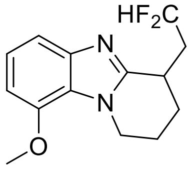

Colourless oil (33 mg, 67% yield); ^{1}H NMR (400 MHz, $CDCl_3$) δ 7.20 (t, *J* = 2.0 Hz, 1H), 6.9 (d, *J* = 2.0 Hz, 1H), 6.69 (d, *J* = 2.0 Hz, 1H), 6.32 (tdd, *J* = 56.7, 5.3, 3.9 Hz, 1H), 4.17-4.12 (m, 1H), 4.01 (s, 3H), 3.97-3.91 (m, 1H), 3.33-3.25 (m, 1H), 2.97-2.82 (m, 1H), 2.32-2.00 (m, 4H), 1.78-1.69 (m, 1H); ^{13}C NMR (100 MHz, $CDCl_3$) δ 151.9, 150.9, 136.1, 132.3, 116.9 (t, *J* = 238.0 Hz), 103.1, 102.4, 55.7, 42.7, 37.9 (t, *J* = 21.0 Hz), 31.2 (dd, *J* = 6.0, 5.0 Hz), 27.4, 21.6; ^{19}F｛H｝NMR (376 MHz, $CDCl_3$) δ -113.3 (d, *J* = 282.0 Hz, 1F), -117.3 (d, *J* = 282.0 Hz, 1F); HRMS (ESI, m/z)[M + H^+]: calculated for $C_{14}H_{17}F_2N_2O$: 267.1298, found 267.1299.

4-(2,2-Difluoroethyl)-7,8-difluoro-1,2,3,4-tetrahydrobenzo[4,5]imidazo[1,2-*a*]pyridine (3e)

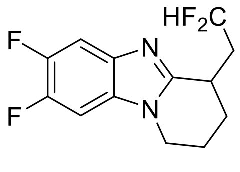

White solid (38.2 mg, 70% yield); m.p. 115.6-116.6 °C;^{1}H NMR (400 MHz, $CDCl_3$) δ 7.50 (dd, *J* = 10.3, 7.2 Hz, 1H), 7.12 (dd, *J* = 9.5, 6.8 Hz, 1H), 6.36 (tdd, *J* = 56.6, 5.3, 3.6 Hz, 1H), 4.25-4.10 (m, 1H), -4.05-3.91 (m, 1H), 3.45-3.29 (m, 1H), 2.88-2.68 (m, 1H), 2.40-2.05 (m, 4H), 1.86-1.70 (m, 1H); ^{13}C NMR (100 MHz, $CDCl_3$) δ 154.6 (d, *J* = 2.3 Hz), 149.4 (d, *J* = 14.0 Hz), 146.6 (dd, J = 15.0, J = 3.0 Hz), 135.9 (d, *J* = 11.0 Hz), 129.9 (d, *J* = 10.6 Hz), 116.7 (t, *J* = 239.0 Hz), 106.2 (d, *J* = 21 Hz), 97.5 (d, *J* = 23.0 Hz), 42.8, 37.7 (t, *J* = 22.0 Hz), 31.1 (dd, *J* = 7.0, 5.0 Hz), 29.71 26.86, 21.4; ^{19}F｛H｝NMR (376 MHz, $CDCl_3$) δ -113.7 (d, *J* = 282.0 Hz, 1F), -117.7 (d, *J* = 282.0 Hz, 1F), -141.3 (d, *J* = 18.8 Hz, 1F), -142.3 (d, *J* = 22.6 Hz, 1F); HRMS (ESI, m/z)[M + H^+]: calculated for $C_{13}H_{13}F_4N_2$: 273.1010, found 273.0999. The spectral data were in accordance with the literature[1].

7,8-Dichloro-4-(2,2-difluoroethyl)-1,2,3,4-tetrahydrobenzo[4,5]imidazo[1,2-*a*]pyridine (3f)

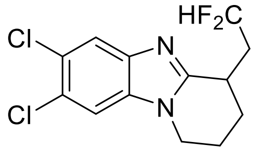

White solid (48.8 mg, 80% yield); m.p. 126.5-127.5 °C; ^{1}H NMR (400 MHz, $CDCl_3$) δ 7.76 (s, 1H), 7.37 (s, 1H), 6.39 (tdd, *J* = 56.7, 5.6, 3.6 Hz, 1H), 4.25-4.05 (m, 1H), 3.92 (td, *J* = 11.4, 4.7 Hz, 1H), 3.35-3.20 (m, 1H), 2.82-2.55 (m, 1H), 2.40-2.00 (m, 4H), 1.82-1.70 (m, 1H); ^{13}C NMR (100 MHz, $CDCl_3$) δ 155.5, 142.0, 134.0, 126.3, 126.1, 120.4, 116.6 (t, *J* = 238.0 Hz), 110.4, 42.7, 37.9 (t, *J* = 21.0 Hz), 31.4 (dd, *J* = 4.3, 2.1 Hz), 27.3, 21.6; ^{19}F｛H｝NMR (376 MHz, $CDCl_3$) δ -113.7 (d, *J* = 285.8 Hz, 1F), -118.0 (d, *J* = 282.0 Hz, 1F); HRMS (ESI, m/z)[M + H^+]: calculated for $C_{13}H_{13}Cl_2F_2N_2$: 305.0418, found 305.0424.The spectral data were in accordance with the literature [1].

7,8-Dibromo-4-(2,2-difluoroethyl)-1,2,3,4-tetrahydrobenzo[4,5]imidazo[1,2-*a*]pyridine (3g)

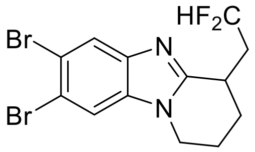

White solid (52mg, 65% yield); m.p. 120.5-121.5 °C; ^{1}H NMR (400 MHz, $CDCl_3$) δ 7.95 (s, 1H), 7.56 (s, 1H), 6.38 (tdd, *J* = 56.7, 5.5, 3.7 Hz, 1H), 4.22-4.07 (m, 1H), 4.00-3.86 (m, 1H), 3.33-3.19 (m, 1H), 2.77-2.59 (m, 1H), 2.35-2.02 (m, 4H), 1.84-1.67 (m, 1H); ^{13}C NMR (100 MHz, $CDCl_3$) δ 155.3, 142.9, 134.8, 123.6, 117.5, 117.3, 116.6 (d, *J* = 237.0 Hz), 113.7, 42.7, 37.9 (t, *J* = 22.0 Hz), 31.3 (dd, *J* = 7.0, 5.0Hz), 27.2, 21.6; ^{19}F｛H｝NMR (376 MHz, $CDCl_3$) δ -113.6 (d, *J* = 282.0 Hz, 1F), -118.0 (d, *J* = 285.8 Hz, 1F); HRMS (ESI, m/z)[M + H^+]: calculated for $C_{13}H_{13}Br_2F_2N_2$: 394.9388, found; 394.9397. The spectral data were in accordance with the literature[1].

4-(2,2-Difluoroethyl)-7,8-dimethyl-1,2,3,4-tetrahydrobenzo[4,5]imidazo[1,2-*a*]pyridine (3h)

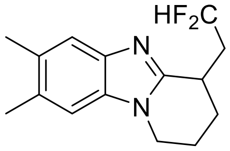

White solid (23.8 mg, 45% yield); m.p. 168.5-169.5 °C; ^{1}H NMR (400 MHz, $CDCl_3$) δ 7.50 (s, 1H), 7.08 (s, 1H), 6.38 (tdd, *J* = 56.8, 5.4, 3.8 Hz, 1H), 4.24-4.08 (m, 1H), 4.02-3.85 (m, 1H), 3.40-3.20 (m, 1H), 2.88-2.67 (m, 1H), 2.39 (d, *J* = 6.9 Hz, 6H), 2.33-2.01 (m, 4H), 1.83-1.69 (m, 1H); ^{13}C NMR (100 MHz, $CDCl_3$) δ 153.1, 141.3, 134.2, 122.9, 122.6, 118.8, 116.7 (t, *J* = 238.0 Hz), 109.2, 42.5, 37.9 (t, *J* = 21.0 Hz), 31.0 (dd, *J* = 6.0, 4.0 Hz), 27.2, 21.5; ^{19}F｛H｝NMR (376 MHz, $CDCl_3$) δ -113.5 (d, *J* = 285.8 Hz, 1F), -117.5 (d, *J* = 282.0 Hz, 1F); HRMS (ESI, m/z)[M + H^+]: calculated for $C_{15}H_{19}F_2N_2$: 265.1511, found 265.1508. The spectral data were in accordance with the literature[1][2].

8-(2,2-Difluoroethyl)-2,3-diphenyl-5,6,7,8-tetrahydroimidazo[1,2-*a*]pyridine (3i)

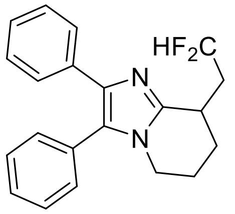

White solid (28.5 mg, 42% yield); m.p. 158.0-159.0 °C; ^{1}H NMR (400 MHz, $CDCl_3$) δ 7.49-7.38 (m, 5H), 7.34-7.31 (m, 2H), 7.20-7.16 (m, 2H), 7.13-7.09 (m, 1H), 6.57 (tdd, *J* = 57.2, 6.2, 3.3 Hz, 1H), 3.74-3.59 (m, 2H), 3.23-3.16 (m, 1H), 2.75-2.60 (m, 1H), 2.24-2.02 (m, 3H), 1.96-1.85 (m, 1H), 1.72-1.62 (m, 1H); ^{13}C NMR (100 MHz, CDCl3) δ 146.2, 135.5, 133.2, 130.7, 130.1, 129.1, 128.8, 128.3, 127.7, 126.9, 126.8, 117.2 (t, *J* = 238.0 Hz), 44.0, 38.6 (t, *J* = 20.0 Hz), 30.3 (dd, *J* = 8.0, 4.0 Hz), 27.1, 22.7; ^{19}F｛H｝NMR (376 MHz, $CDCl_3$) δ -113.0 (d, *J* = 282.0 Hz, 1F), -118.2 (d, *J* = 282.0 Hz, 1F); HRMS (ESI, m/z)[M + H^+]: calculated for $C_{21}H_{20}F_2N_2$: 339.1668, found 339.1646. The spectral data were in accordance with the literature[1].

Dimethyl 8-(2,2-difluoroethyl)-5,6,7,8-tetrahydroimidazo[1,2-*a*]pyridine-2,3-dicarboxylate (3j)

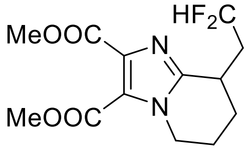

White solid (42 mg, 70% yield); m.p. 90.4-91.4 °C; ^{1}H NMR (400 MHz, $CDCl_3$) δ 6.22 (tdd, *J* = 56.5, 5.3, 3.8 Hz, 1H), 4.38-4.31 (m, 1H), 4.10-4.01 (m, 1H), 3.90 (d, *J* = 5.4 Hz, 6H), 3.20-3.10 (m, 1H), 2.80-2.60 (m, 1H), 2.28 – 1.95 (m, 4H), 1.72-1.61 (m, 1H).; ^{13}C NMR (100 MHz, $CDCl_3$) δ 163.4, 160.5, 149.6, 136.2, 124.0, 116.5 (t, *J* = 238.0 Hz), 52.4, 52.3, 45.6, 37.9 (t, *J* = 21.0 Hz), 30.9 (dd, *J* = 7.0, 5.0 Hz), 26.5, 21.6; ^{19}F｛H｝NMR (376 MHz, $CDCl_3$) δ -113.6 (d, *J* = 282.0 Hz, 1F), -117.6 (d, *J* = 282.0 Hz, 1F); HRMS (ESI, m/z)[M + H^+]: calculated for $C_{13}H_{17}F_2N_2O_4$: 303.1151, found 303.1156. The spectral data were in accordance with the literature[1].

Methyl 8-(2,2-difluoroethyl)-5,6,7,8-tetrahydroimidazo[1,2-*a*]pyridine-3-carboxylate (3k)

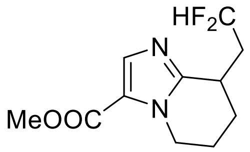

Colourless oil (39 mg, 80% yield); ^{1}H NMR (400 MHz, $CDCl_3$) δ 7.70(s, 1H), 6.30 (tdd, *J* = 56.6, 5.5, 3.6 Hz, 1H), 4.58-4.44 (m, 1H), 4.15-4.07 (m, 1H), 3.85 (s, 3H), 3.24-3.17 (m, 1H), 2.71-2.55 (m, 1H), 2.25-1.96 (m, 4H), 1.73-1.67 (m, 1H); ^{13}C NMR (100 MHz, $CDCl_3$) δ 160.6, 151.3, 135.3, 122.3, 116.5 (t, *J* = 238.0 Hz), 51.3, 45.4, 38.0 (t, *J* = 21.0 Hz), 30.8 (dd, *J* = 7.0, 5.0 Hz), 29.7, 26.4, 21.62; ^{19}F｛H｝NMR (376 MHz, $CDCl_3$) δ -113.7 (d, *J* = 285.8 Hz, 1F), -117.8 (d, *J* = 285.8 Hz, 1F); HRMS (ESI, m/z)[M + H^+]: calculated for $C_{11}H_{14}F_2N_2O_2$: 245.1066, found 245.1065.

3-(2,2-Difluoroethyl)-2,3-dihydro-1*H*-benzo[*d*]pyrrolo[1,2-*a*]imidazole (3l)

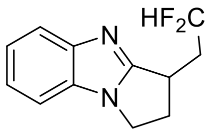

Colourless oil (29.5 mg, 66% yield); ^{1}H NMR (400 MHz, CDCl3) δ ^{1}H NMR (400 MHz, Chloroform-*d*) δ 7.76-7.67 (m, 1H), 7.36-7.28 (m, 1H), 7.29-7.20 (m, 2H), 6.26 (tdd, *J* = 56.2, 5.3, 3.2 Hz, 1H), 4.25-4.15 (m, 1H), 4.13-3.95 (m, 1H), 3.61-3.50 (m, 1H), 3.05-2.95 (m, 1H), 2.72-2.56 (m, 1H), 2.54-2.42 (m, 1H), 2.27-2.10 (m, 1H).; ^{13}C NMR (100 MHz, $CDCl_3$) δ 161.5, 147.9, 132.1, 122.5, 122.2, 119.7, 116.1 (t, *J* = 238 Hz), 109.7, 42.2, 37.1 (t, *J* = 21.0 Hz), 33.7, 30.7 (dd, *J* = 7.0, 5.0 Hz); ^{19}F｛H｝NMR (376 MHz, $CDCl_3$) δ -114.9 (d, *J* = 282.0 Hz, 1F), -118.0 (d, *J* = 285.8 Hz, 1F); HRMS (ESI, m/z)[M + H^+]: calculated for $C_{12}H_{13}F_2N_2$: 223.1042, found 223.1060. The spectral data were in accordance with the literature[1].

3-(2,2-Difluoroethyl)-8-methyl-2,3-dihydro-1*H*-benzo[*d*]pyrrolo[1,2-*a*]imidazole (3m)

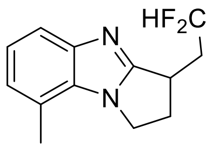

Colourless oil (25 mg, 53% yield); ^{1}H NMR (400 MHz, $CDCl_3$) δ 7.18-7.12 (m, 2H), 7.06-7.04 (m, 1H), 6.22 (tdd, *J* = 56.1, 5.2, 3.4 Hz, 1H), 4.20-4.14 (m, 1H), 4.07-4.01 (m, 1H), 3.03-2.95 (m, 1H), 2.77-2.67 (m, 1H), 2.65 (s, 3H), 2.53-2.43 (m, 1H), 2.21-2.13 (m, 1H); ^{13}C NMR (100 MHz, $CDCl_3$) δ 160.8, 147.6, 131.8, 129.8, 122.5, 122.2, 116.2 (t, *J* = 238.0 Hz), 107.2, 42.0, 37.3 (t, *J* = 21.0 Hz), 30.7 (dd, *J* = 6.0, 4.0 Hz), 16.9; ^{19}F｛H｝NMR (376 MHz, $CDCl_3$) δ -114.9 (d, *J* = 285.8 Hz, 1F), -117.7 (d, *J* = 282.0 Hz, 1F); HRMS (ESI, m/z)[M + H^+]: calculated for $C_{13}H_{14}F_2N_2$: 237.1148, found 237.1149.

6,7-Dichloro-3-(2,2-difluoroethyl)-2,3-dihydro-1*H*-benzo[*d*]pyrrolo[1,2-*a*]imidazole (3n).

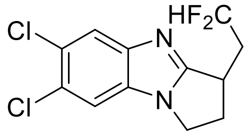

White solid (28 mg, 48% yield); m.p. 125.1-126.1 °C; ^{1}H NMR (400 MHz, $CDCl_3$) δ 7.78 (s, 1H), 7.42 (s, 1H), 6.24 (tdd, *J* = 56.0, 5.3, 3.2 Hz, 1H), 4.19-4.14 (m, 1H), 4.07-4.01 (m, 1H), 3.58-3.50 (m, 1H), 3.06-2.98 (m, 1H), 2.64-2.48 (m, 2H), 2.26-2.15 (m, 1H); ^{13}C NMR (100 MHz, $CDCl_3$) δ 163.8, 147.7, 131.4, 126.3, 126.1, 121.0, 115.8 (t, J = 239.0 Hz), 111.0, 42.4, 36.9 (t, *J* = 21.0 Hz), 33.8, 30.7 (dd, *J* = 7.0, 5.0 Hz), 29.7; ^{19}F｛H｝NMR (376 MHz, $CDCl_3$) δ -115.1 (d, *J* = 285.8 Hz, 1F), -118.1 (d, *J* = 282.0 Hz, 1F); HRMS (ESI, m/z)[M + H^+]: calculated for $C_{12}H_{10}Cl_2F_2N_2$: 291.0262, found 292.0264.

6-(2,2-Difluoroethyl)-7,8,9,10-tetrahydro-6*H*-benzo[4,5]imidazo[1,2-*a*]azepine (3o)

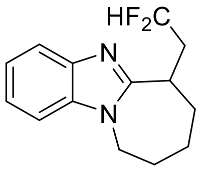

Colourless oil (23.5 mg, 47% yield); ^{1}H NMR (400 MHz, $CDCl_3$) δ 7.80-7.71 (m, 1H), 7.35-7.20 (m, 3H), 6.36 (tdd, *J* = 57.4, 6.3, 3.3 Hz, 1H), 4.49-4.35 (m, 1H), 4.08-3.96 (m, 1H), 3.38-3.25 (m, 1H), 3.05-2.87 (m, 1H), 2.32-1.97 (m, 4H), 1.96-1.82 (m, 1H), 1.67-1.51 (m, 2H); ^{13}C NMR (100 MHz, $CDCl_3$) δ 157.3, 141.2, 135.3, 122.6, 122.0, 119.2, 116.7 (t, *J* = 238.0 Hz), 108.9, 44.1, 37.4 (t, *J* = 21.0 Hz), 34.2 (dd, *J* = 7.0, 4.0 Hz), 32.6, 29.3, 28.1; ^{19}F｛H｝NMR (376 MHz, $CDCl_3$) δ -114.1 (d, *J* = 282.0 Hz, 1F), -118.4 (d, *J* = 285.8 Hz, 1F); HRMS (ESI, m/z): calculated for $C_{14}H_{17}F_2N_2$: 251.1355, found 251.1365. The spectral data were in accordance with the literature[1].

6-(2,2-Difluoroethyl)-1-methyl-7,8,9,10-tetrahydro-6*H*-benzo[4,5]imidazo[1,2-*a*]azepine (3p)

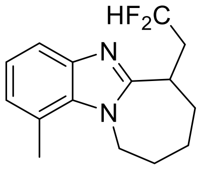

Colourless oil (23.3 mg, 44% yield); ^{1}H NMR (400 MHz, $CDCl_3$) δ 7.17-7.09 (m, 2H), 7.02 (d, *J* = 7.0 Hz, 1H), 6.41 (tdd, *J* = 57.6, 6.7, 3.0 Hz, 1H), 4.36-4.31 (m, 1H), 3.98-3.91 (m, 1H), 3.25-3.19 (m, 1H), 3.01 -2.84 (m, 1H), 2.64 (s, 3H), 2.23-2.12 (m, 2H), 2.04-1.92 (m, 2H), 1.89-1.78 (m, 1H), 1.63-1.49 (m, 2H); ^{13}C NMR (100 MHz, $CDCl_3$) δ 156.3, 135.0, 129.4, 122.5, 117.0 (t, *J* = 237.0 Hz), 106.3, 44.3, 37.4 (t, *J* = 22.0 Hz), 34.3 (t, *J* = 6.0 Hz), 32.5, 28.9, 28.2, 16.7; ^{19}F｛H｝NMR (376 MHz, $CDCl_3$) δ -113.9 (d, *J* = 282.0 Hz, 1F), -118.7 (d, *J* = 282.0 Hz, 1F); HRMS (ESI, m/z)[M + H^+]: calculated for $C_{15}H_{18}F_2N_2$: 265.1498, found 265.1497.

5-(2,2-Difluoroethyl)-5,6-dihydrobenzo[4,5]imidazo[2,1-*a*]isoquinoline (3q)

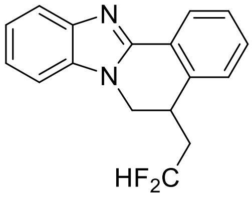

Colourless oil (39.8 mg, 70% yield); ^{1}H NMR (400 MHz, $CDCl_3$) δ 8.35-8.32 (m, 1H), 7.87-7.82 (m, 1H), 7.50-7.42 (m, 2H), 7.40-7.28 (m, 4H), 5.78 (tdd, *J* = 56.2, 3.5, 3.1 Hz, 1H), 4.46(dd, *J* = 12.8, 2.2 Hz, 1H), 4.32-4.27 (m, 1H), 3.60-3.55 (m, 1H), 2.22-1.96 (m, 2H); ^{13}C NMR (100 MHz, $CDCl_3$) δ 148.3, 143.9, 136.6, 134.9, 130.6, 128.6, 127.9, 126.3, 126.0, 123.1, 122.8, 119.9, 115.9 (t, *J* = 238.0 Hz), 109.1, 44.8, 37.8 (t, *J* = 21.0 Hz), 33.2 (dd, J = 6.0, 3.0 Hz); ^{19}F｛H｝NMR (376 MHz, $CDCl_3$) δ -115.7 (d, *J* = 285.8 Hz, 1F), -117.6 (d, *J* = 285.8 Hz, 1F); HRMS (ESI, m/z)[M + H^+]: calculated for $C_{17}H_{15}F_2N_2$:285.1200, found 285.1202.The spectral data were in accordance with the literature [1][3].

4-(2,2-Difluoro-2-phenylethyl)-1,2,3,4-tetrahydrobenzo[4,5]imidazo[1,2-*a*]pyridine (3r)

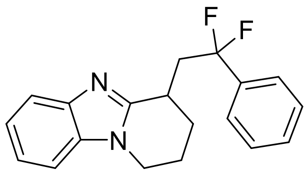

Colourless oil (31.2 mg, 50% yield); ^{1}H NMR (400 MHz, $CDCl_3$) δ 7.72-7.68 (m, 1H), 7.60-7.57 (m, 2H), 7.45-7.40 (m, 3H), 7.27-7.19 (m, 3H), 4.17-4.10 (m, 1H),3.95-3.88 (m, 1H), 3.51-3.32 (m, 2H), 2.53-2.18 (m, 3H), 2.03-1.97 (m, 1H), 1.77-1.72 (m, 1H); ^{13}C NMR (100 MHz, $CDCl_3$) δ 153.7, 142.5, 137.2 (t, *J* = 260.0 Hz), 134.9, 129.9, 128.6, 125.0 (t, *J* = 6.0 Hz), 123.1 (t, *J* = 242.0 Hz), 122.3, 119.1, 109.0, 42.5, 42.2 (t, *J* = 27.0 Hz), 32.1, 27.3, 21.7; ^{19}F ｛H｝ NMR (376 MHz, $CDCl_3$) δ -89.1 (d, *J* = 244.4 Hz, 1F), -98.5 (d, *J* = 244.4 Hz, 1F); HRMS (ESI, m/z)[M + H^+]: calculated for $C_{19}H_{18}F_2N_2$: 313.1512, found 313.1513.

6-Bromo-4-(2,2-difluoro-2-phenylethyl)-1,2,3,4-tetrahydrobenzo[4,5]imidazo[1,2-*a*]pyridine (3s)

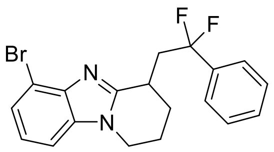

Colourless oil (31.3 mg, 40% yield); ^{1}H NMR (400 MHz, $CDCl_3$) δ 7.72-7.68 (m, 1H), 7.60-7.57 (m, 2H), 7.45-7.40 (m, 3H), 7.27-7.19 (m, 3H), 4.17-4.10 (m, 1H),3.95-3.88 (m, 1H), 3.51-3.32 (m, 2H), 2.53-2.18 (m, 3H), 2.03-1.97 (m, 1H), 1.77-1.72 (m, 1H); ^{13}C NMR (100 MHz, $CDCl_3$) δ 154.5, 141.3, 137.3 (t, *J* = 27.0 Hz), 135.4, 129.9, 129.9, 128.5, 125.3, 125.0(t, *J* = 6.0 Hz), 123.1 (t, *J* = 241.0 Hz), 122.9, 112.6, 108.3, 42.9, 42.0 (t, *J* = 26.0 Hz), 32.2, 26.9, 21.5; ^{19}F ｛H｝ NMR (376 MHz, $CDCl_3$) δ -89.1 (d, *J* = 244.4 Hz, 1F), -98.8 (d, *J* = 244.4 Hz, 1F); HRMS (ESI, m/z)[M + H^+]: calculated for $C_{19}H_{18}F_2N_2$: 392.0605, found 392.0600.

4-(2,2-Difluoro-2-phenylethyl)-9-methoxy-1,2,3,4-tetrahydrobenzo[4,5]imidazo[1,2-*a*]pyridine (3t)

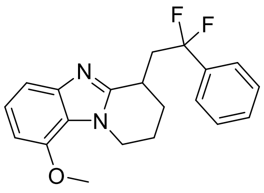

Colourless oil (22 mg, 32% yield); ^{1}H NMR (400 MHz, $CDCl_3$) δ 7.64-7.54 (m, 2H), 7.48-7.38 (m, 3H), 7.16 (t, J = 8.0 Hz, 1H), 6.9-6.86 (m, 1H), 6.67 (d, J = 7.9 Hz, 1H), 4.20-4.13 (m, 1H), 3.99 (s, 3H), 3.98-3.90 (m, 1H), 3.70-3.50 (m, 1H), 3.41-3.31 (m, 1H), 2.60-2.35 (m, 1H), 2.41-2.20 (m, 2H), 2.08-1.96 (m, 1H), 1.85-1.70 (m, 1H); ^{13}C NMR (100 MHz, $CDCl_3$) δ 152.3, 151.0, 137.4 (t, *J* = 27.0 Hz), 136.3, 132.6, 129.8, 128.5, 125.0(t, *J* = 6.0 Hz), 123.3 (t, *J* = 242.0 Hz), 122.8, 112.6, 102.6, 101.97, 55.6, 42.7, 42.1 (t, *J* = 27.0 Hz), 32.1, 27.2, 21.8; ^{19}F ｛H｝ NMR (376 MHz, $CDCl_3$) δ -88.8 (d, *J* = 244.4 Hz, 1F), -99.1 (d, *J* = 244.4 Hz, 1F); HRMS (ESI, m/z)[M + H^+]: calculated for $C_{19}H_{18}F_2N_2$: 343.1684, found 313.1688.

4-(2,2-Difluoro-2-phenylethyl)-7,8-difluoro-1,2,3,4-tetrahydrobenzo[4,5]imidazo[1,2-*a*]pyridine (3u)

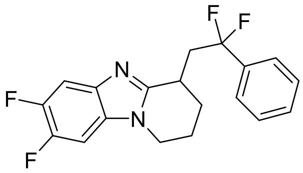

White solid (36.5 mg, 52% yield); m.p. 112.6-113.6 °C; ^{1}H NMR (400 MHz, $CDCl_3$) δ 7.64-7.54 (m, 2H), 7.48-7.38 (m, 3H), 7.16 (t, J = 8.0 Hz, 1H), 6.9-6.86 (m, 1H), 6.67 (d, J = 7.9 Hz, 1H), 4.20-4.13 (m, 1H), 3.99 (s, 3H), 3.98-3.90 (m, 1H), 3.70-3.50 (m, 1H), 3.41-3.31 (m, 1H), 2.60-2.35 (m, 1H), 2.41-2.20 (m, 2H), 2.08-1.96 (m, 1H), 1.85-1.70 (m, 1H); ^{13}C NMR (100 MHz, $CDCl_3$) δ 155.2 (d, *J* = 3.0 Hz), 149.8 (dd, *J* = 240.0, 4.0 Hz), 147.6 (dd, *J* = 240.0, 4.0 Hz) 137.6 (dd, *J* = 11.0, 2.0 Hz), 137.08 (t, *J* = 26.0 Hz) , ,130.1, 130.0, 128.3, 128.6, 124.9 (t, *J* = 6.0 Hz), 123.3 (t, *J* = 242.0 Hz), 122.8, 123.03 (t, *J* = 241.0 Hz), 106.4 (d, *J* = 19.0 Hz), 97.04 (d, *J* =22.0 Hz), 42.7, 42.1 (t, *J* = 26.0 Hz), 32.2, 27.1, 21.6; ^{19}F｛H｝NMR (376 MHz, $CDCl_3$) δ -88.8 (d, *J* = 244.4 Hz, 1F), -99.1 (d, *J* = 244.4 Hz, 1F), -142.8 (d, J = 18.8 Hz, 1F), -143.9 (d, J = 22.5 Hz, 1F);; HRMS (ESI, m/z)[M + H^+]: calculated for $C_{19}H_{18}F_2N_2$: 349.1300, found 349.1302.

7,8-Dichloro-4-(2,2-difluoro-2-phenylethyl)-1,2,3,4-tetrahydrobenzo[4,5]imidazo[1,2-*a*]pyridine (3v)

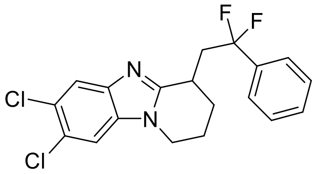

White solid (34.3 mg, 45% yield); m.p. 128.3-129.3 °C; ^{1}H NMR (400 MHz, $CDCl_3$) δ 7.75 (s, 1H), 7.61-7.53 (m, 2H), 7.49-7.41 (m, 3H), 7.35 (s, 1H), 4.18-1.06(m, 1H), 3.97-3.81 (m, 1H), 3.50-3.30 (m, 2H), 2.59-2.48 (m, 1H), 2.45-2.22 (m, 2H), 2.11-1.97 (m, 1H), 1.85-1.69 (m, 1H); ^{13}C NMR (100 MHz, $CDCl_3$) δ 155.9, 141.9, 137.0 (t, *J* = 26.0 Hz), 134.2, 130.0, 128.6, 128.5, 126.0, 125.4, 124.9 (t, *J* = 6.0 Hz), 123.0 (t, *J* = 242.0 Hz), 110.4, 42.7, 42.0 (t, *J* = 27.0 Hz), 32.2, 27.0, 21.6; ^{19}F｛H｝NMR (376 MHz, $CDCl_3$) δ -89.5 (d, *J* = 244.4 Hz, 1F), -98.5 (d, *J* = 244.4 Hz, 1F); HRMS (ESI, m/z)[M + H^+]: calculated for $C_{19}H_{18}F_2N_2$: 381.0859, found 381.0859.

4-(2,2-Difluoro-2-phenylethyl)-7,8-dimethyl-1,2,3,4-tetrahydrobenzo[4,5]imidazo[1,2-*a*]pyridine (3w)

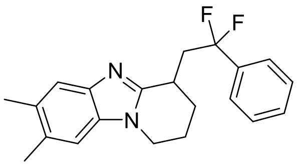

White solid (32.1 mg, 47% yield); m.p. 137.4-138.4 °C; ^{1}H NMR (400 MHz, $CDCl_3$) δ 7.62-7.55 (m, 2H), 7.48-7.36 (m, 4H), 7.04 (s, 1H), 4.18-4.08 (m, 1H), 3.95-3.85 (m, 1H), 3.54-3.28 (m, 2H), 2.55-2.46 (m, 1H), 2.36 (d, J = 7.4 Hz, 6H), 2.28 – 2.19 (m, 1H), 2.10-1.93 (m, 2H), 1.80-1.68 (m, 1H); ^{13}C NMR (100 MHz, $CDCl_3$) δ 152.8, 141.1,

137.3 (t, *J* = 26.0 Hz), 133.4, 131.0, 130.9, 129.9, 128.5, 125.0(t, *J* = 6.0 Hz), 123.2 (t, *J* = 243.0 Hz), 119.2, 109.3, 42.4, 42.2 (t, *J* = 27.0 Hz), 32.1, 27.3, 27.8, 20.5, 20.4; ^{19}F {H} NMR (376 MHz, $CDCl_3$) δ -88.9 (d, *J* = 244.4 Hz, 1F), -98.6 (d, *J* = 244.4 Hz, 1F); HRMS (ESI, m/z)[M + H^+]: calculated for $C_{19}H_{18}F_2N_2$: 341.1955, found 341.1956.

Dimethyl 8-(2,2-difluoro-2-phenylethyl)-5,6,7,8-tetrahydroimidazo[1,2-*a*]pyridine-2,3-dicarboxylate (3w)

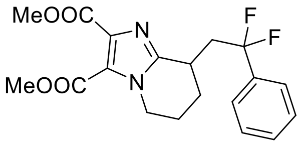

Colourless oil (28.1 mg, 37% yield); ^1H NMR (400 MHz, $CDCl_3$) δ 7.56-7.50 (m, 2H), 7.46-7.38 (m, 3H), 4.38-4.28 (m, 1H), 4.08-3.98 (m, 1H), 3.89 (d, J = 2.1 Hz, 6H), 3.40-3.20 (m, 2H), 2.48-2.39 (m, 1H), 2.36-2.11 (m, 2H), 1.98-1.84 (m, 1H), 1.78-1.66 (m, 1H); ^{13}C NMR (100 MHz, $CDCl_3$) δ 163.3, 160.5, 149.9, 137.1 (t, *J* = 26.0), 136.0, 130.0, 129.9, 128.5, 128.4, 124.9(t, *J* = 6.0), 124.3, 122.9 (t, *J* = 242.0 Hz), 52.4, 52.3, 45.6, 42.1 (t, *J* = 27.0 Hz), 31.7, 26.2, 21.6; ^{19}F {H} NMR (376 MHz, $CDCl_3$) δ -89.7 (d, *J* = 244.4 Hz, 1F), -98.9 (d, *J* = 244.4 Hz, 1F); HRMS (ESI, m/z): calculated for $C_{19}H_{18}F_2N_2$: 379.1434, found 379.1440.

Copies of ^{1}H, ^{13}C, and ^{19}F NMR spectra

4-(2,2-Difluoroethyl)-1,2,3,4-tetrahydrobenzo[4,5]imidazo[1,2-*a*]pyridine (3a)

^{1}H NMR (400 MHz, $CDCl_3$)

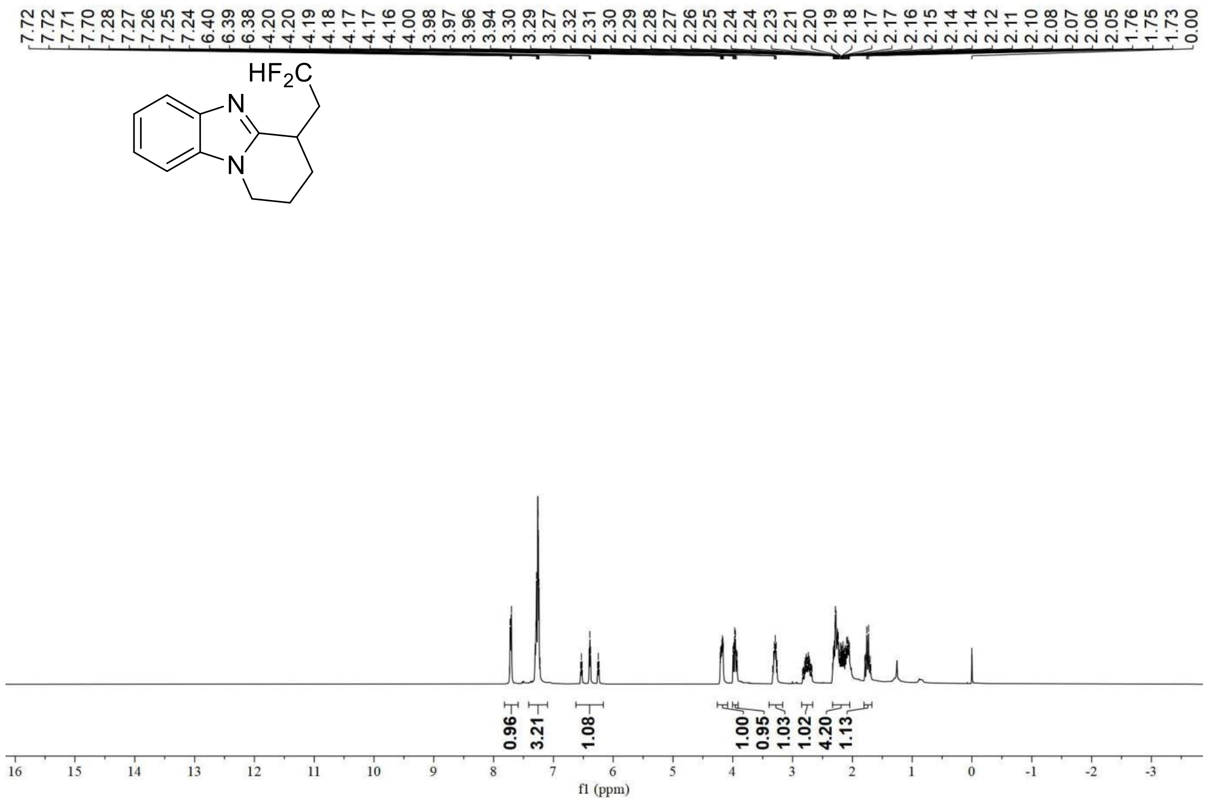

^{13}C NMR (100 MHz, $CDCl_3$)

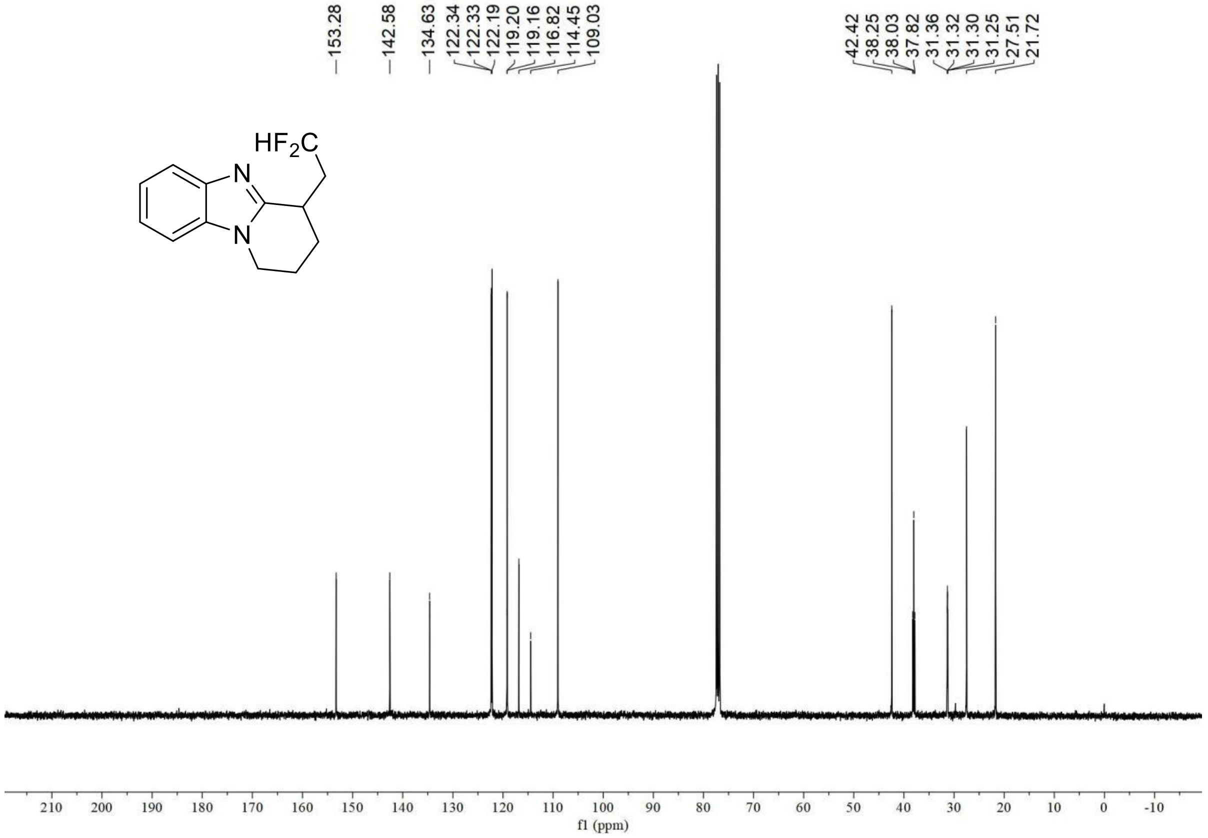

$^{19}F\{H\}$ NMR (376 MHz, $CDCl_3$)

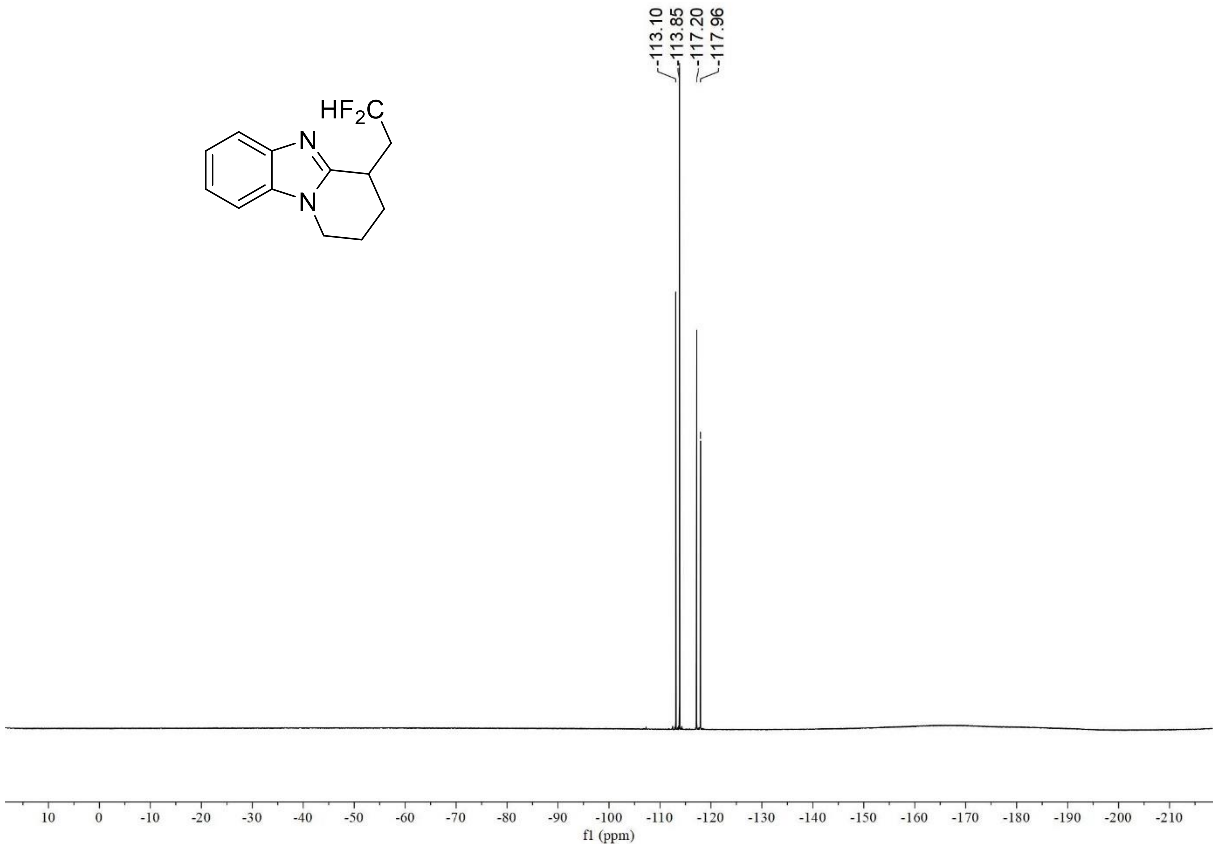

6-Bromo-4-(2,2-difluoroethyl)-1,2,3,4-tetrahydrobenzo[4,5]imidazo[1,2-*a*]pyridine (3b)

^{1}H NMR (400 MHz, $CDCl_3$)

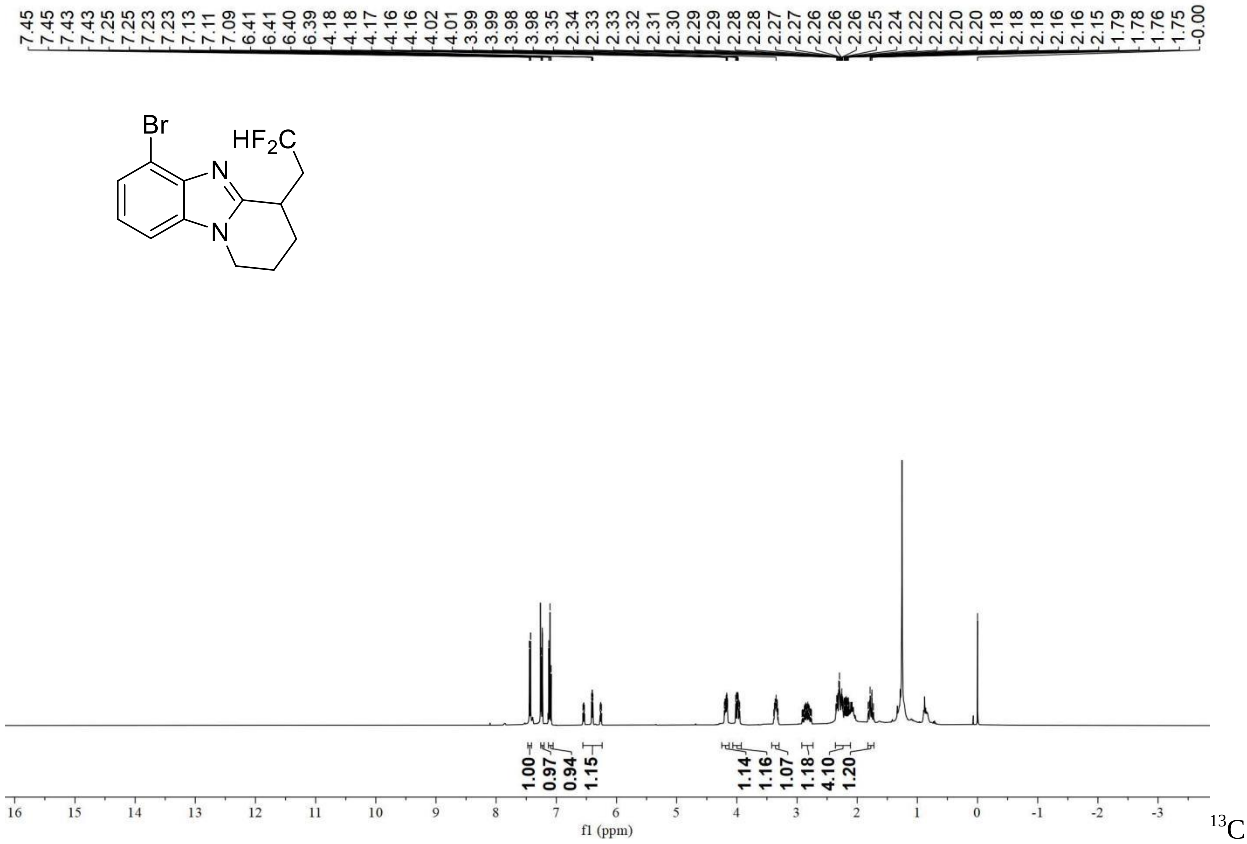

^{13}C

NMR (100 MHz, $CDCl_3$)

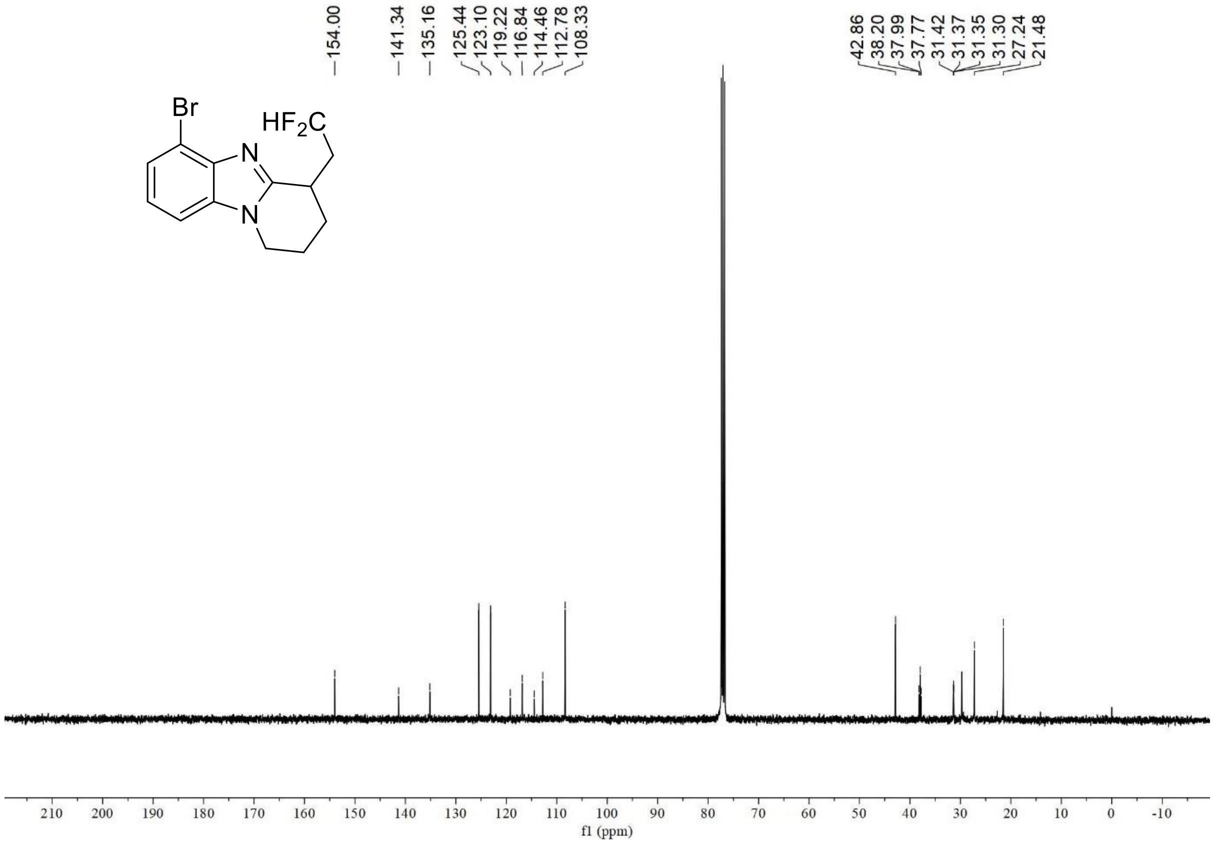

^{19}F｛H｝NMR (376 MHz, $CDCl_3$)

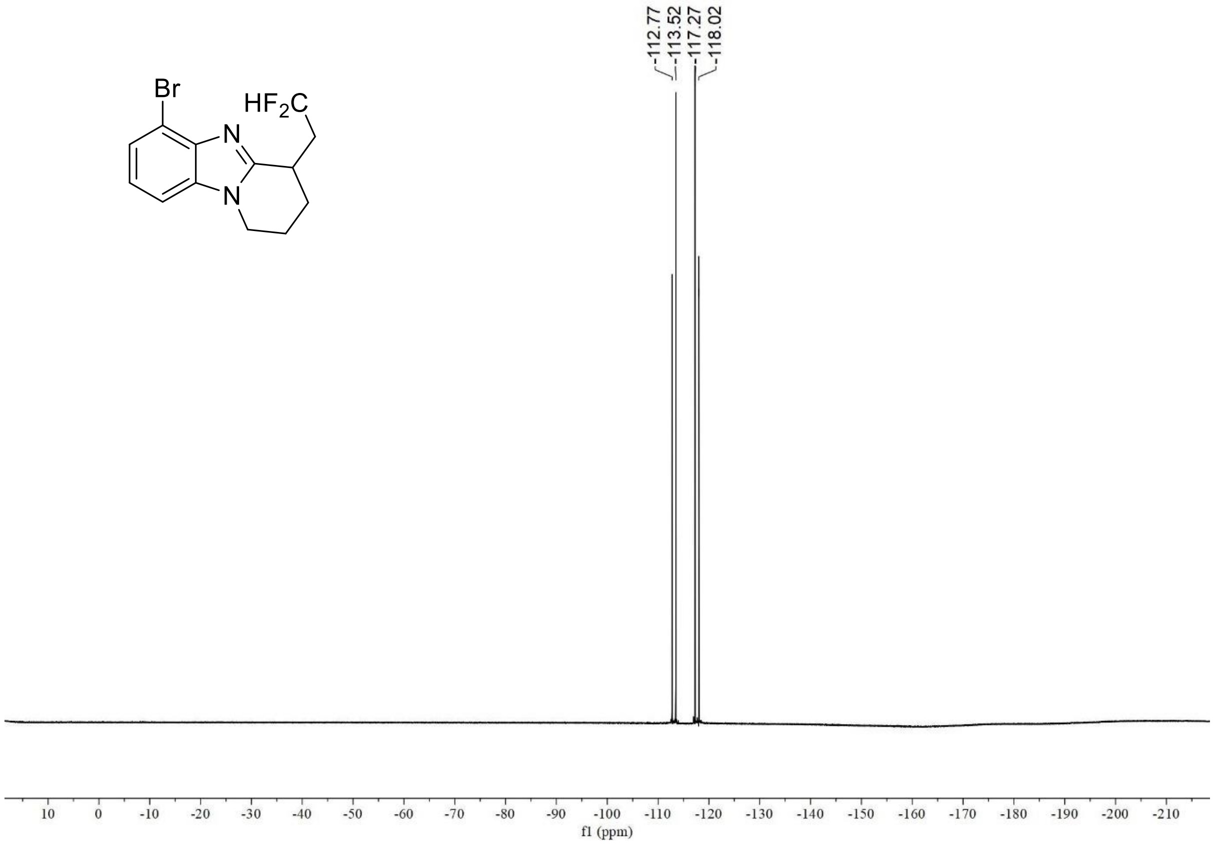

4-(2,2-Difluoroethyl)-9-methyl-1,2,3,4-tetrahydrobenzo[4,5]imidazo[1,2-*a*]pyridine (3c)

^{1}H NMR (400 MHz, $CDCl_3$)

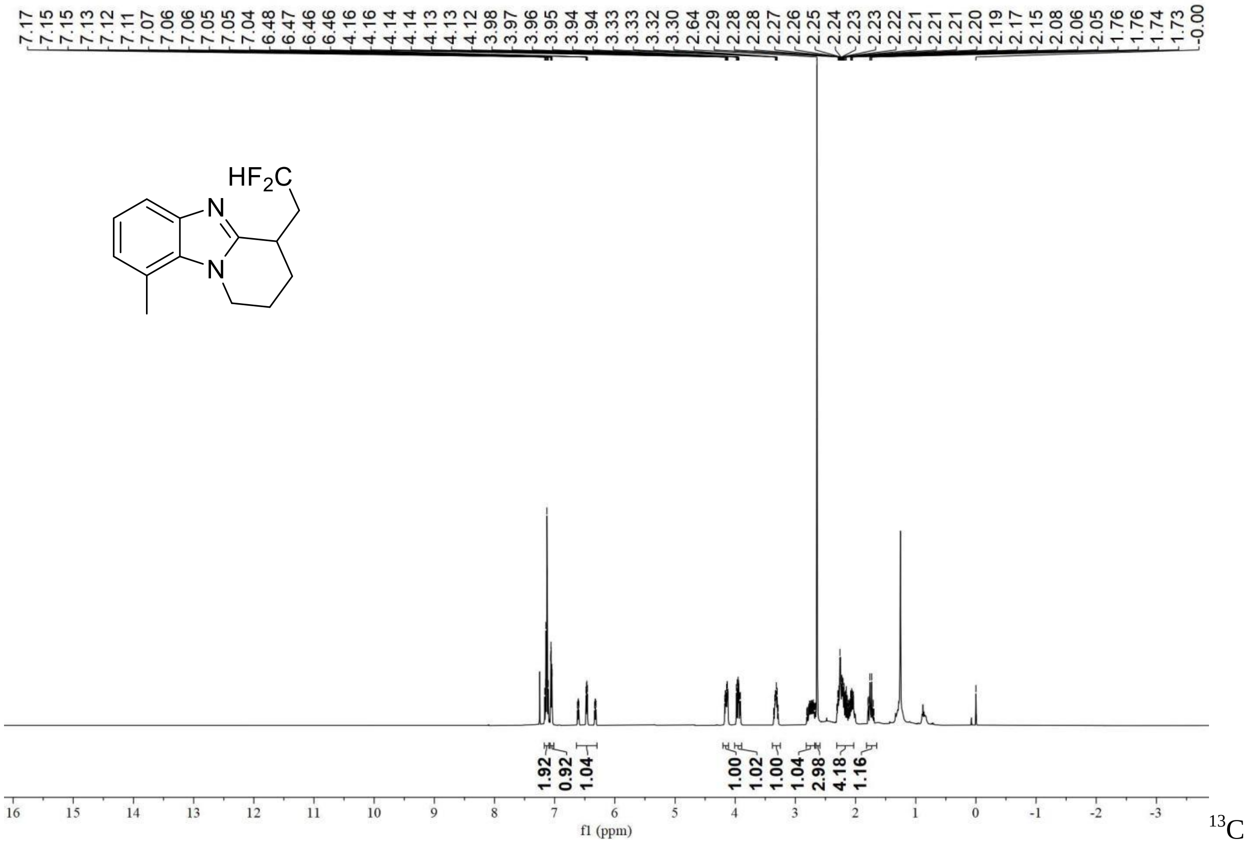

^{13}C NMR (100 MHz, $CDCl_3$)

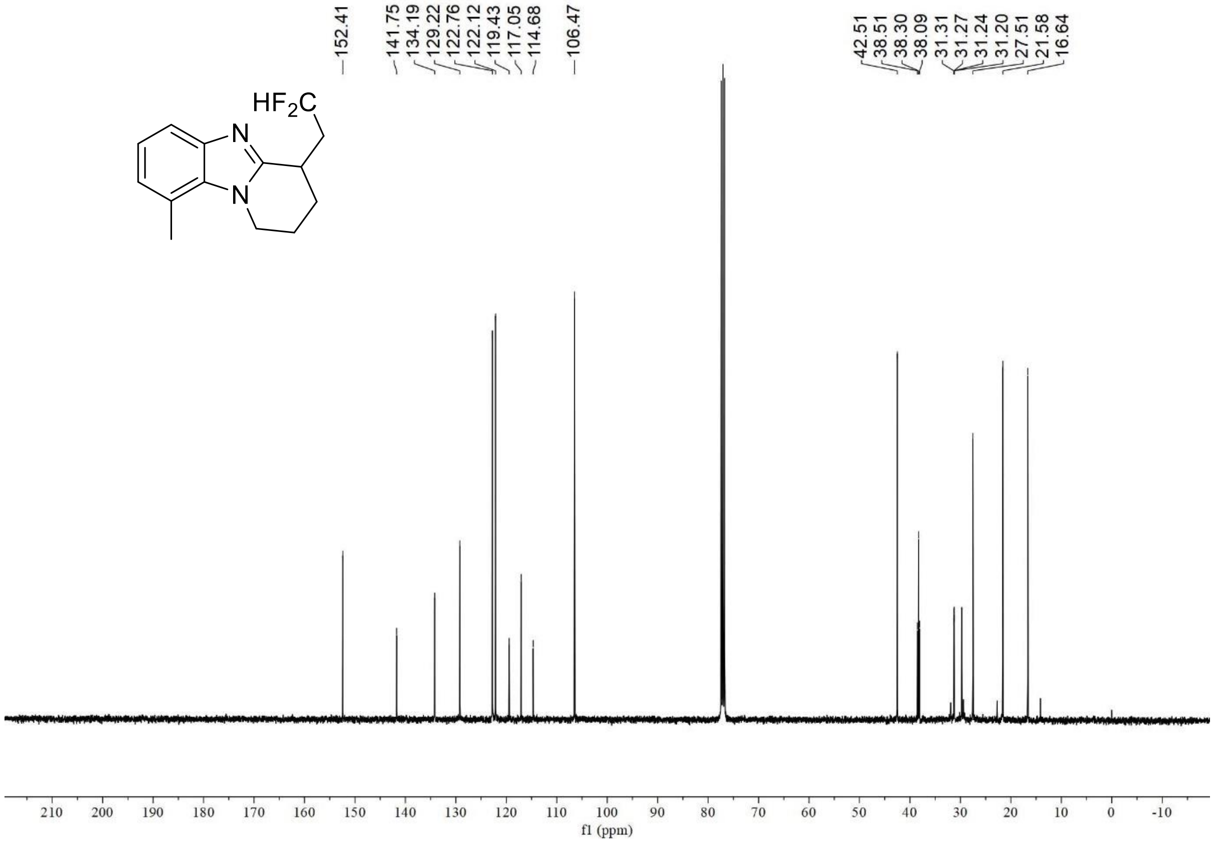

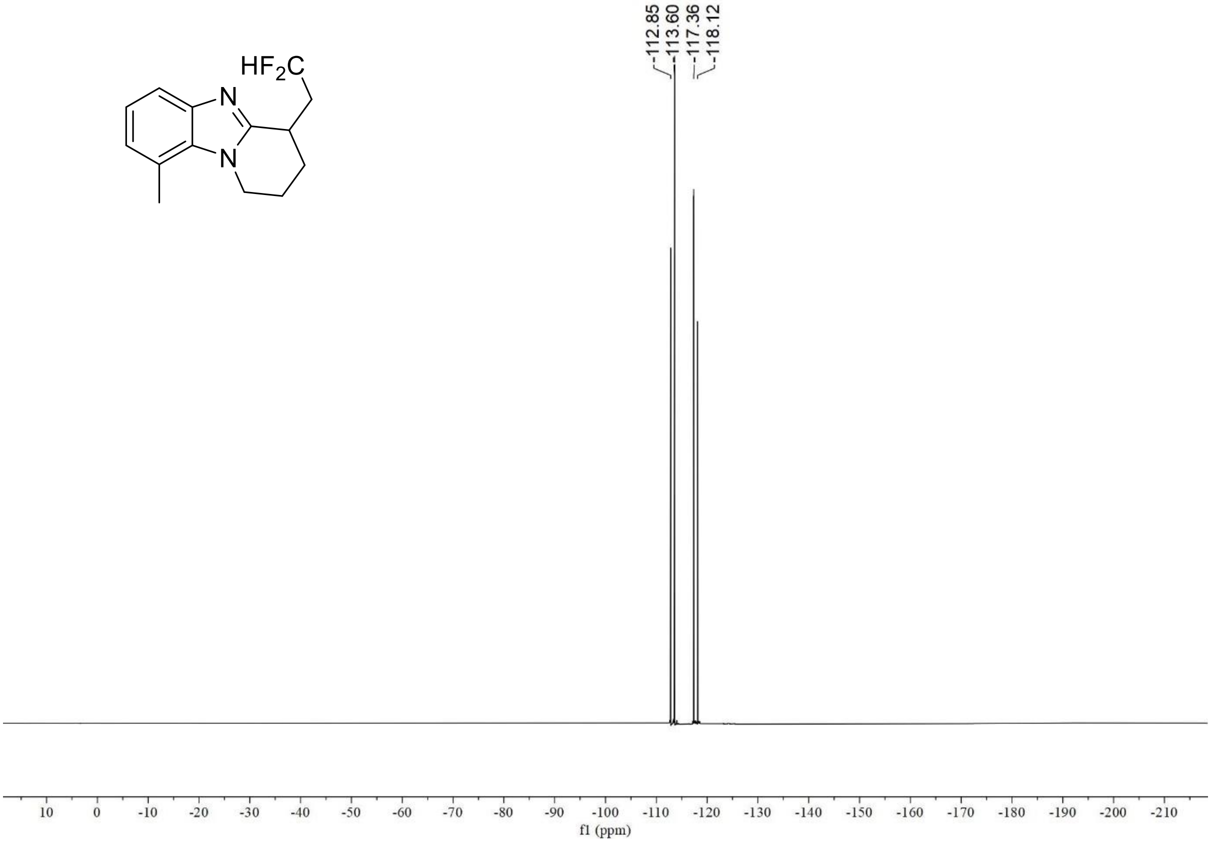
HF2C
N
N
-112.85
-113.60
-117.36
-118.12
10 0 -10 -20 -30 -40 -50 -60 -70 -80 -90 -100 -110 -120 -130 -140 -150 -160 -170 -180 -190 -200 -210
f1 (ppm)

4-(2,2-Difluoroethyl)-9-methoxy-1,2,3,4-tetrahydrobenzo[4,5]imidazo[1,2-*a*]pyridine (3d)

^{1}H NMR (400 MHz, $CDCl_3$)

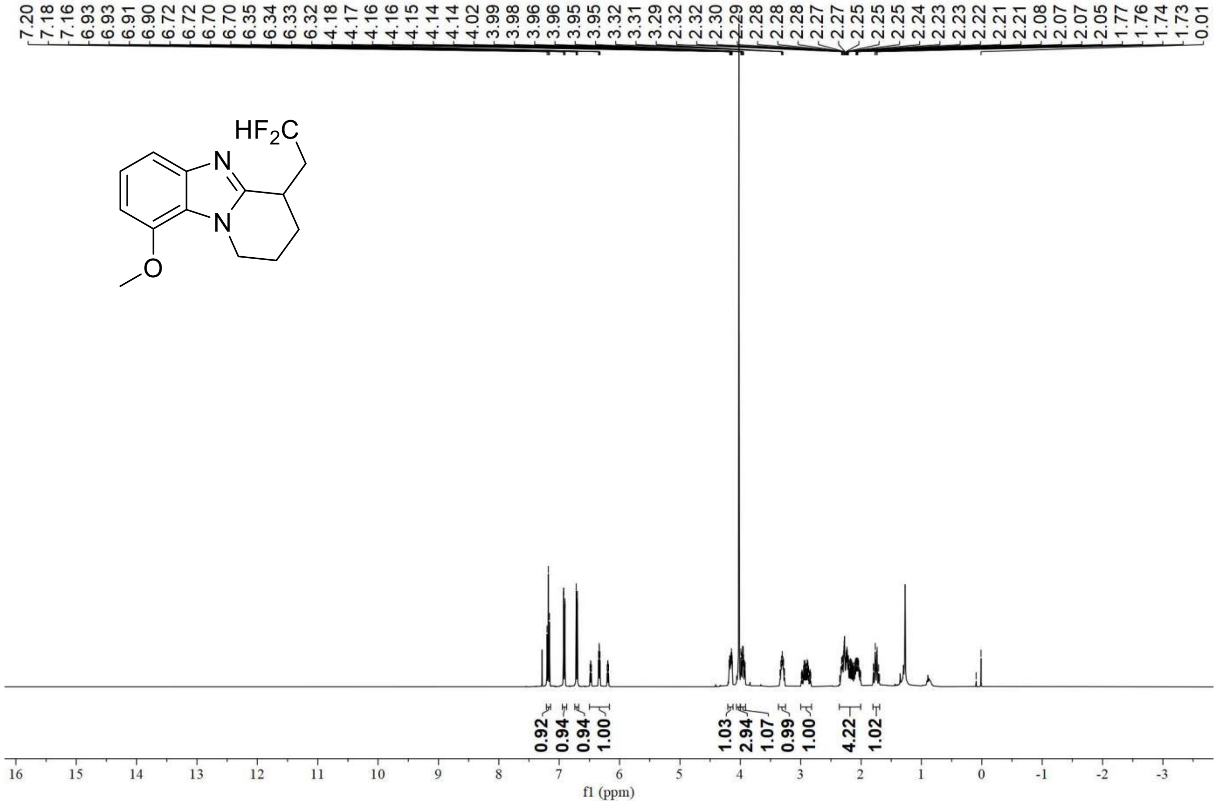

^{13}C NMR (100 MHz, $CDCl_3$)

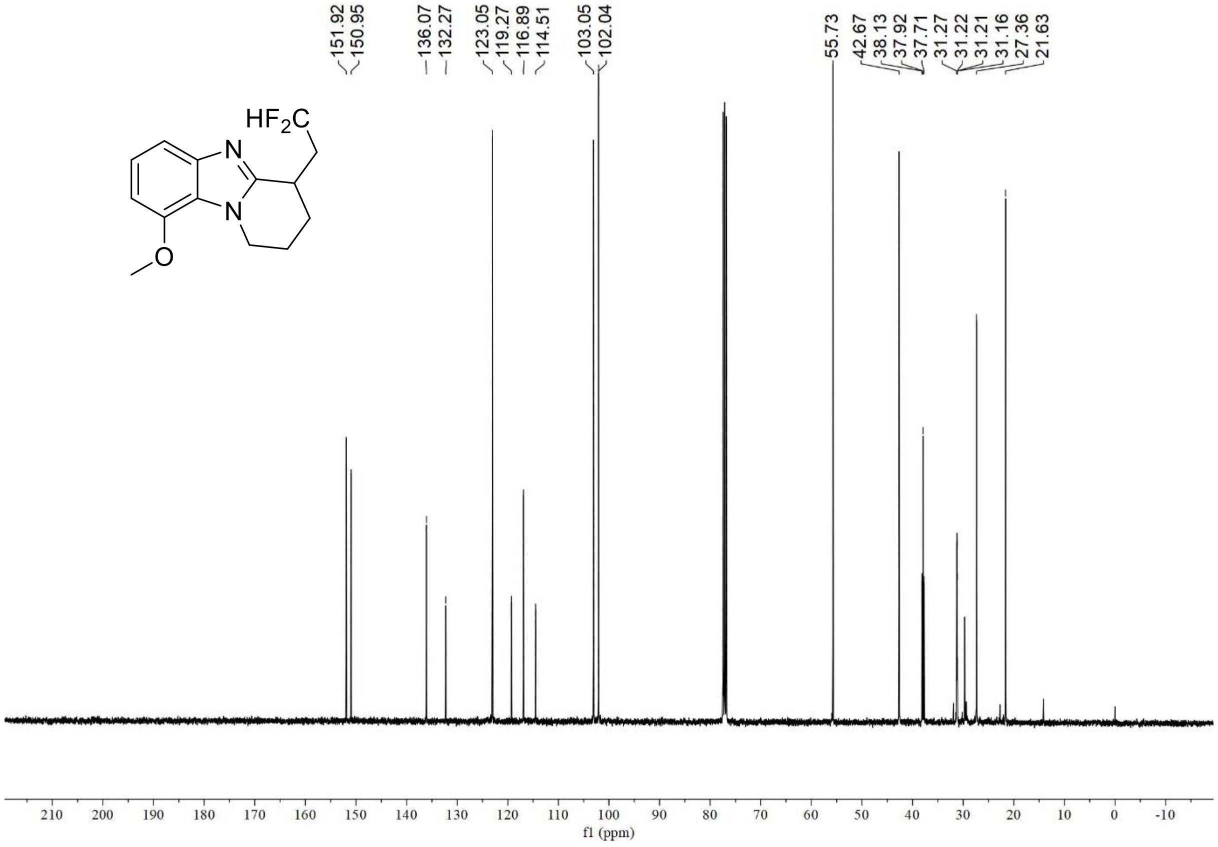

^{19}F｛H｝NMR (376 MHz, $CDCl_3$)

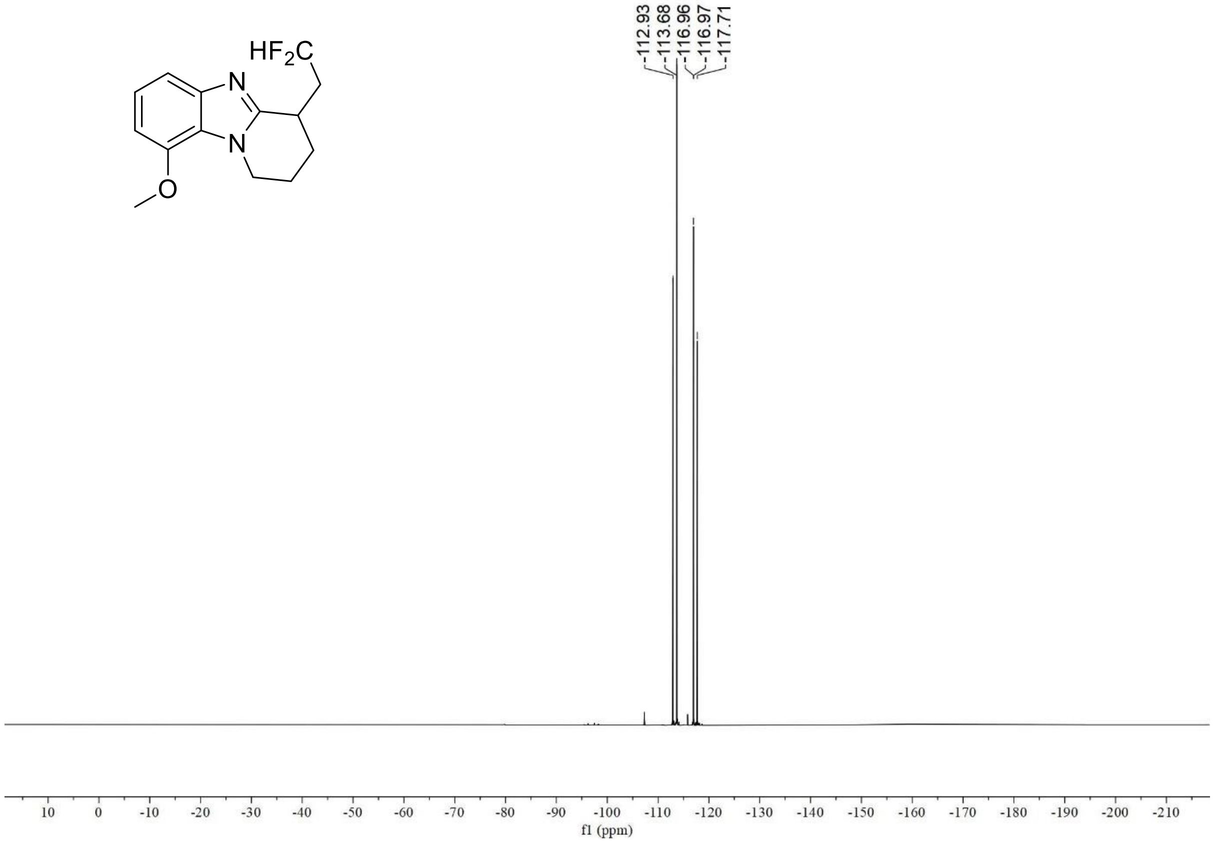

4-(2,2-Difluoroethyl)-7,8-difluoro-1,2,3,4-tetrahydrobenzo[4,5]imidazo[1,2-*a*]pyridine (3e)

^{1}H NMR (400 MHz, $CDCl_3$)

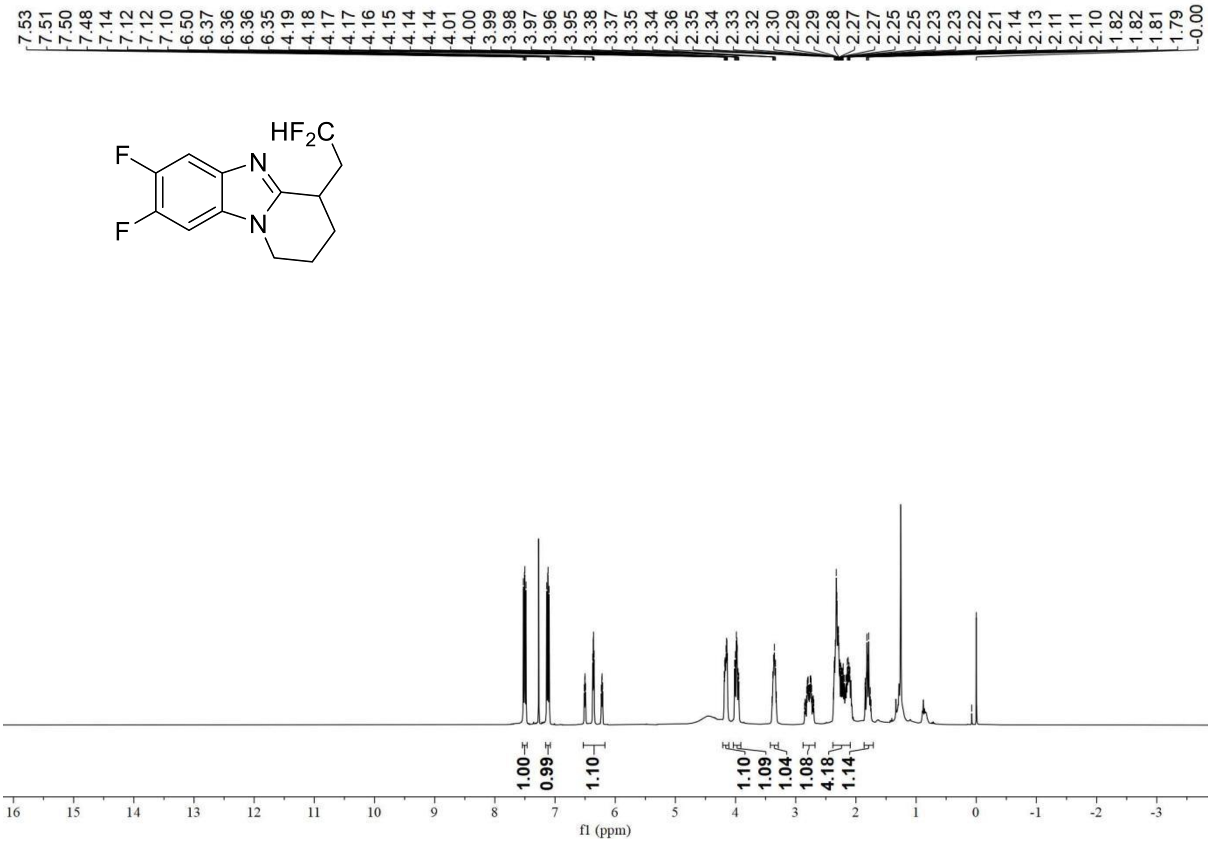

^{13}C NMR (100 MHz, $CDCl_3$)

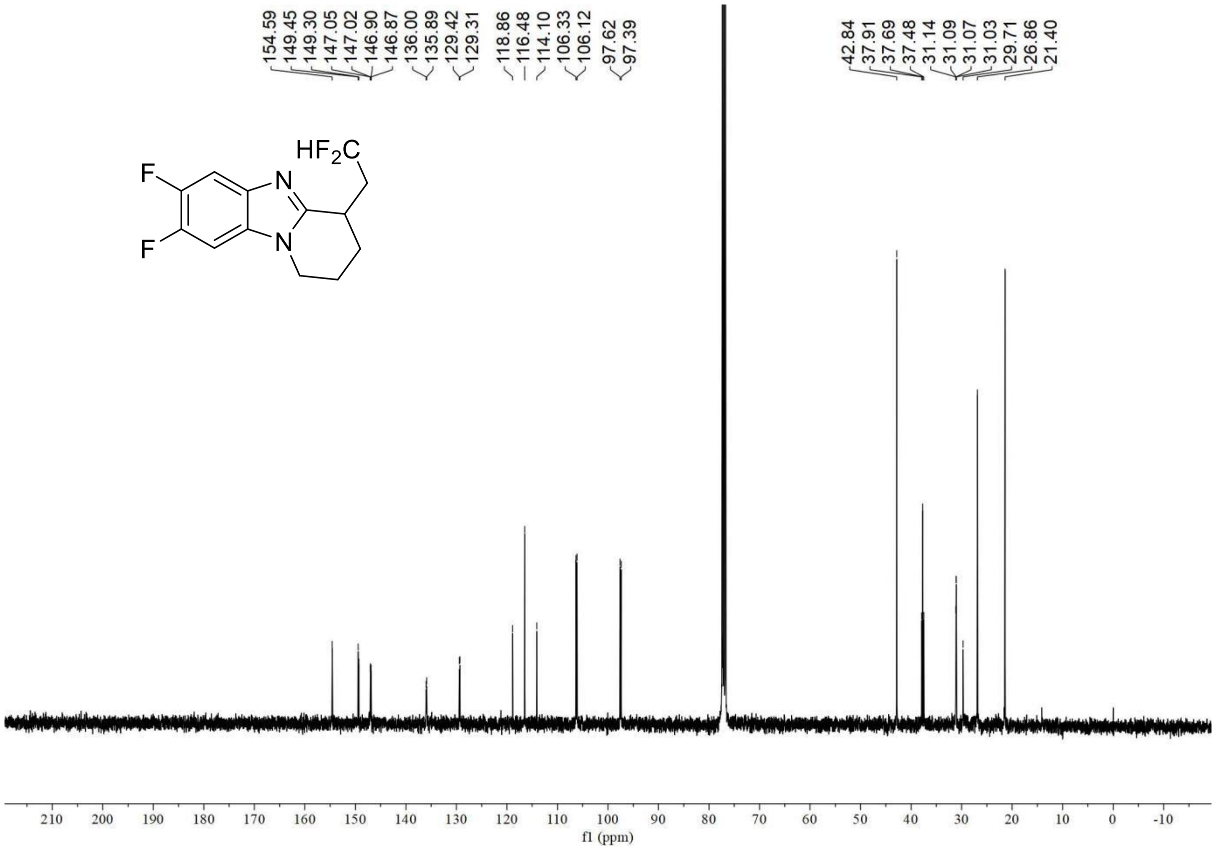

$^{19}F\{H\}$ NMR (376 MHz, $CDCl_3$)

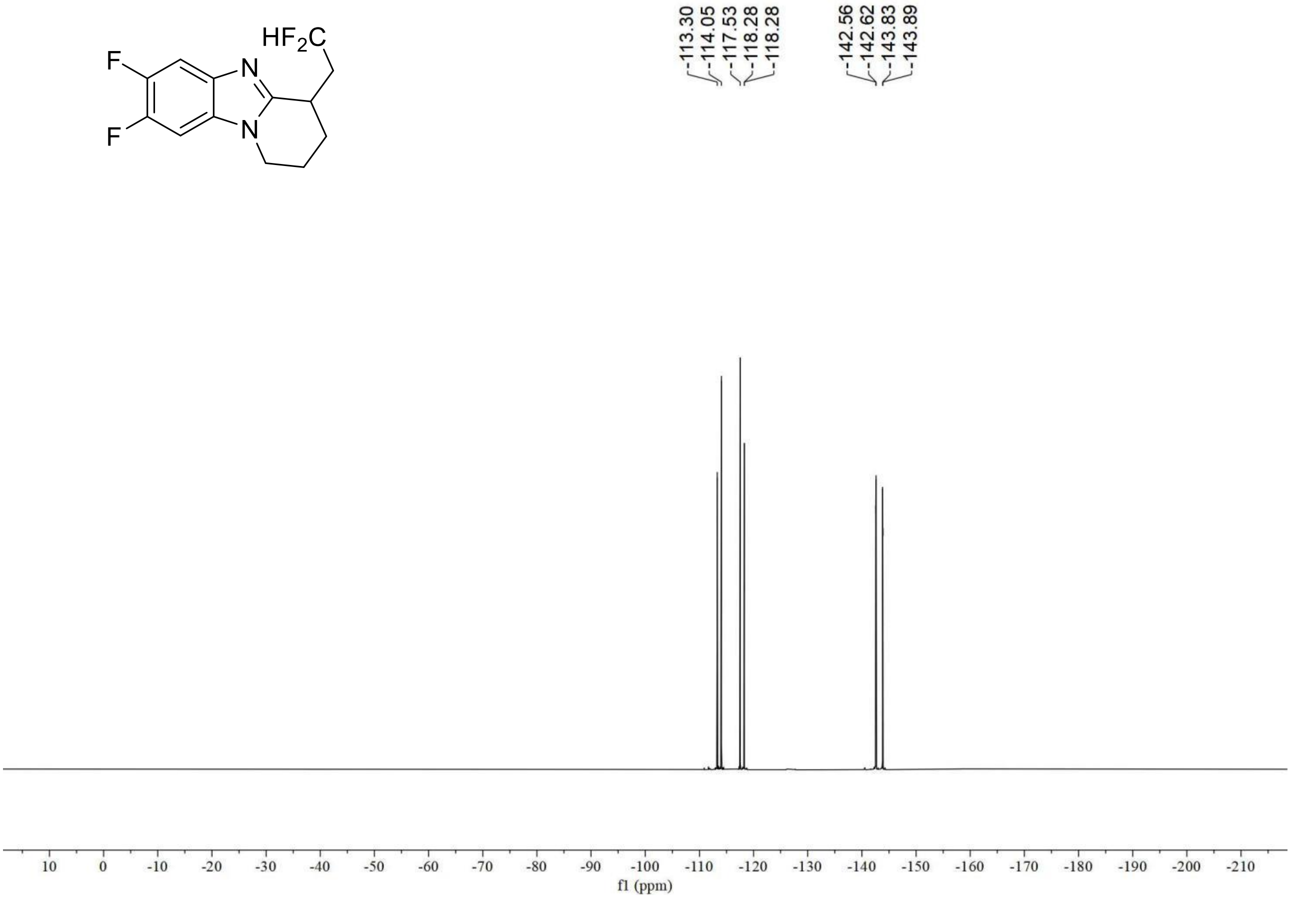

7,8-Dichloro-4-(2,2-difluoroethyl)-1,2,3,4-tetrahydrobenzo[4,5]imidazo[1,2-*a*]pyridine (3f)

^{1}H NMR (400 MHz, $CDCl_3$)

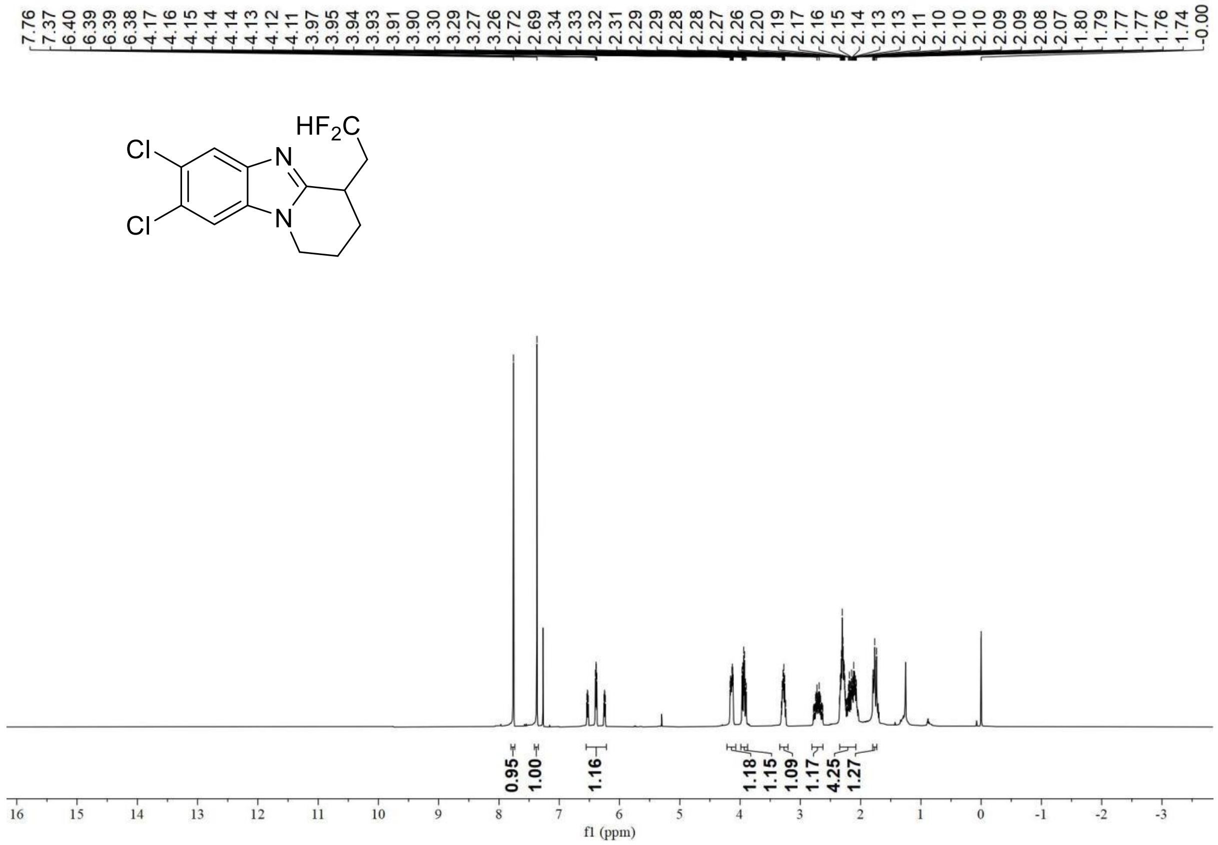

^{13}C NMR (100 MHz, $CDCl_3$)

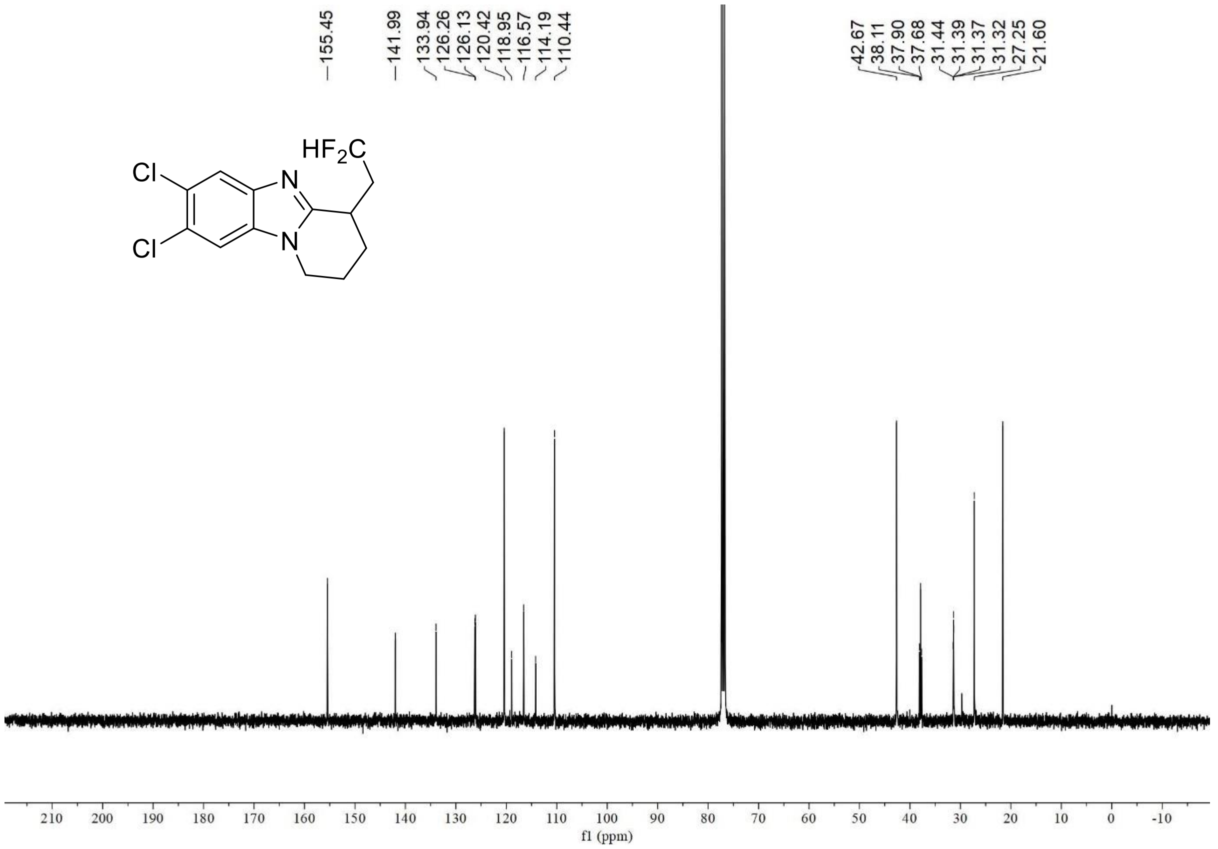

$^{19}F\{H\}$ NMR (376 MHz, $CDCl_3$)

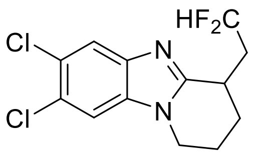

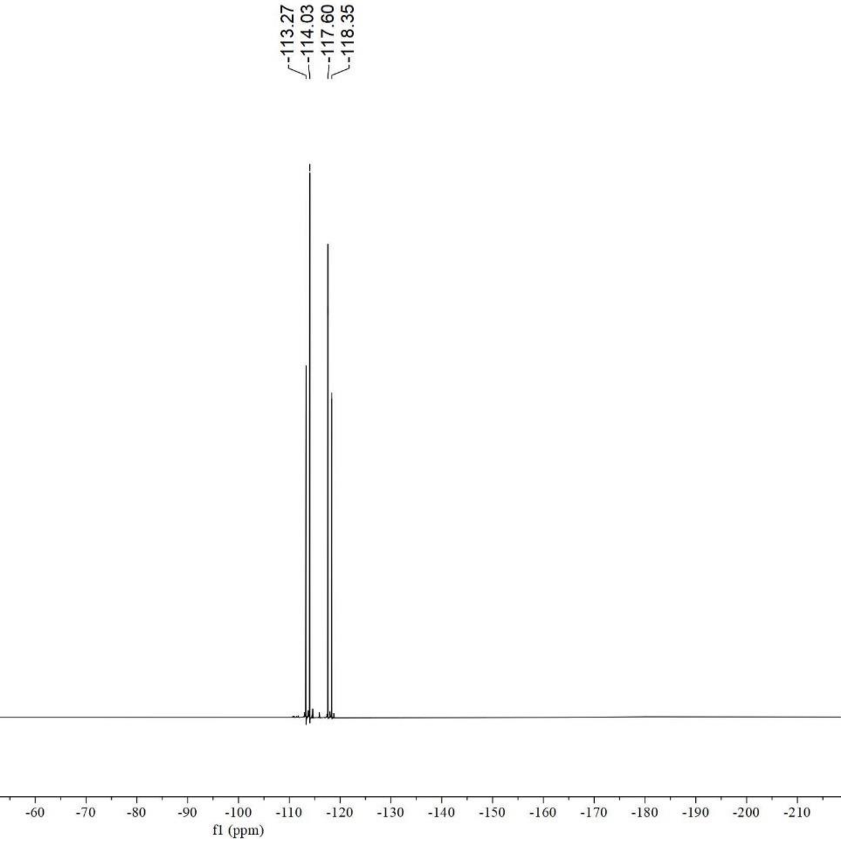

7,8-Dibromo-4-(2,2-difluoroethyl)-1,2,3,4-tetrahydrobenzo[4,5]imidazo[1,2-*a*]pyridine (3g)

^{1}H NMR (400 MHz, $CDCl_3$)

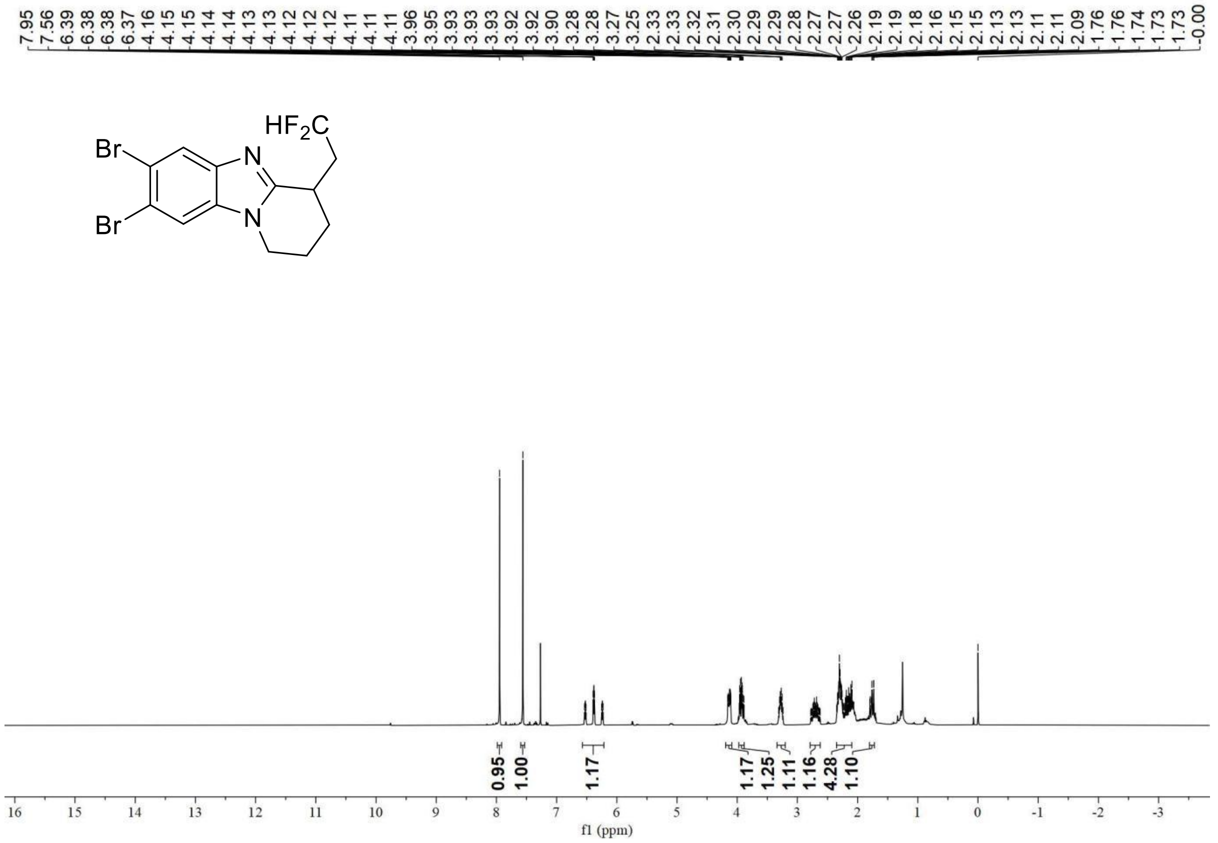

^{13}C NMR (100 MHz, $CDCl_3$)

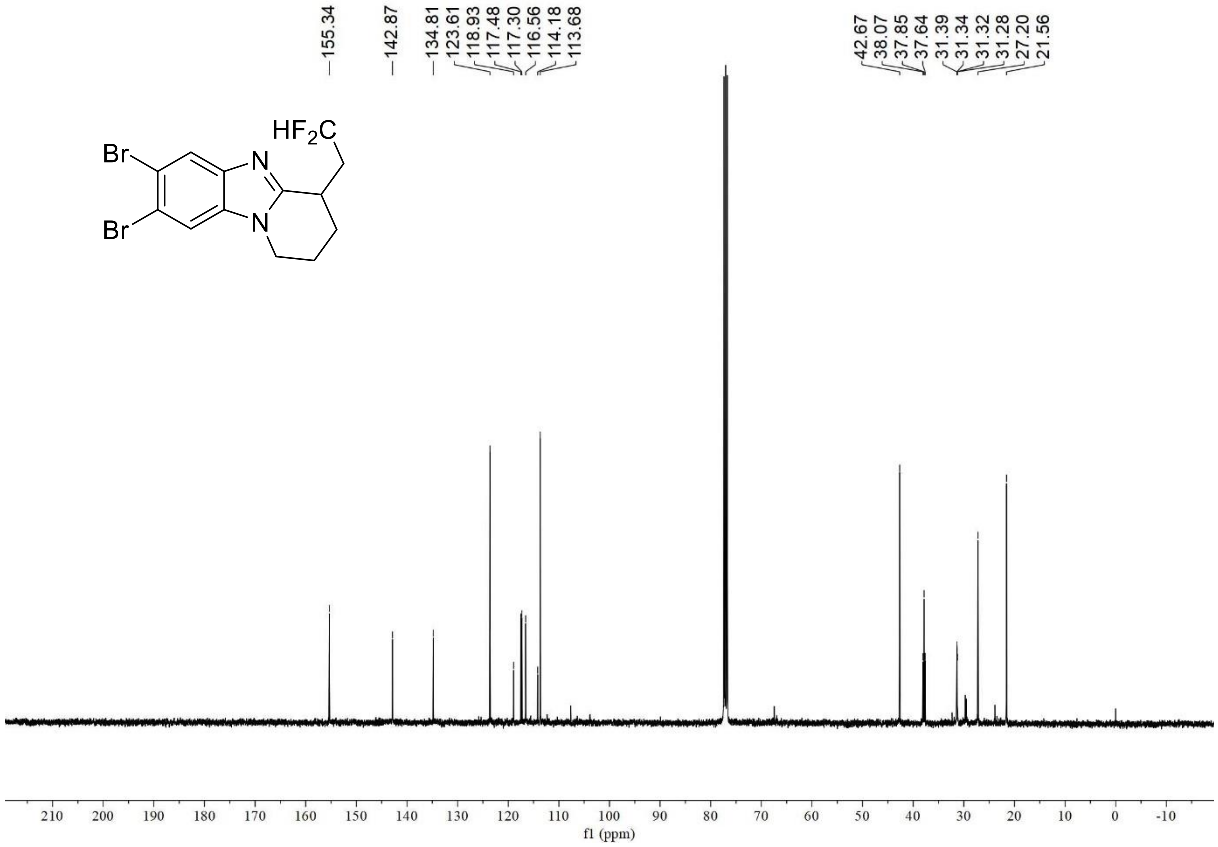

$^{19}F\{H\}$ NMR (376 MHz, $CDCl_3$)

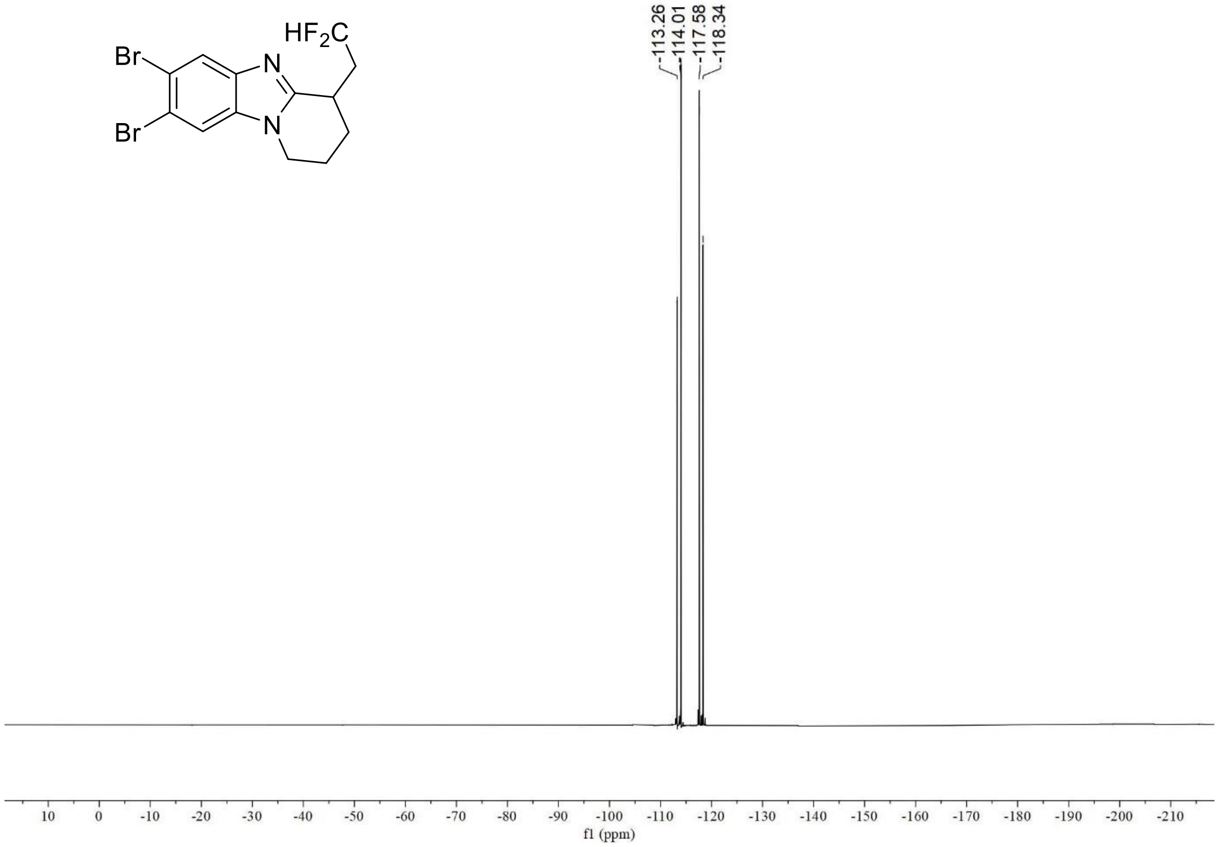

4-(2,2-Difluoroethyl)-7,8-dimethyl-1,2,3,4-tetrahydrobenzo[4,5]imidazo[1,2-*a*]pyridine (3h)

^{1}H NMR (400 MHz, $CDCl_3$)

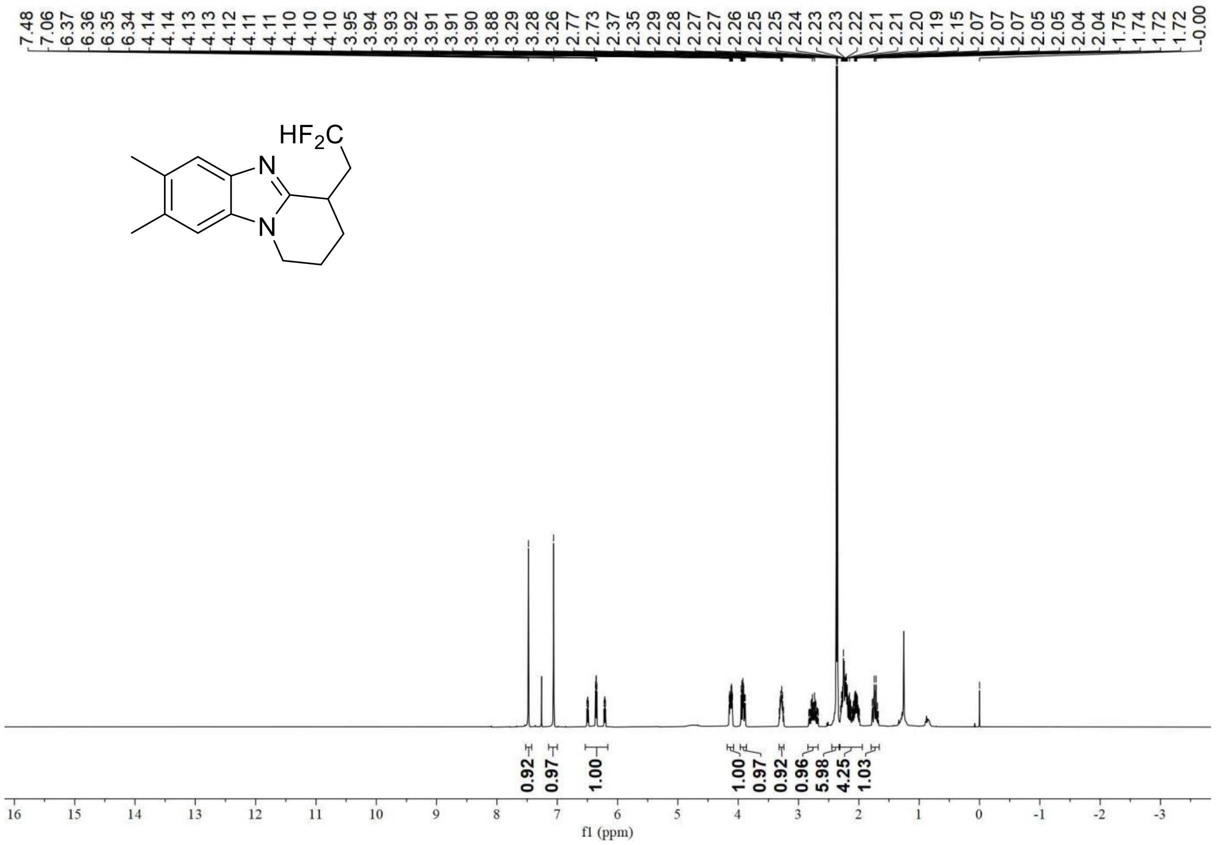

^{13}C NMR (100 MHz, $CDCl_3$)

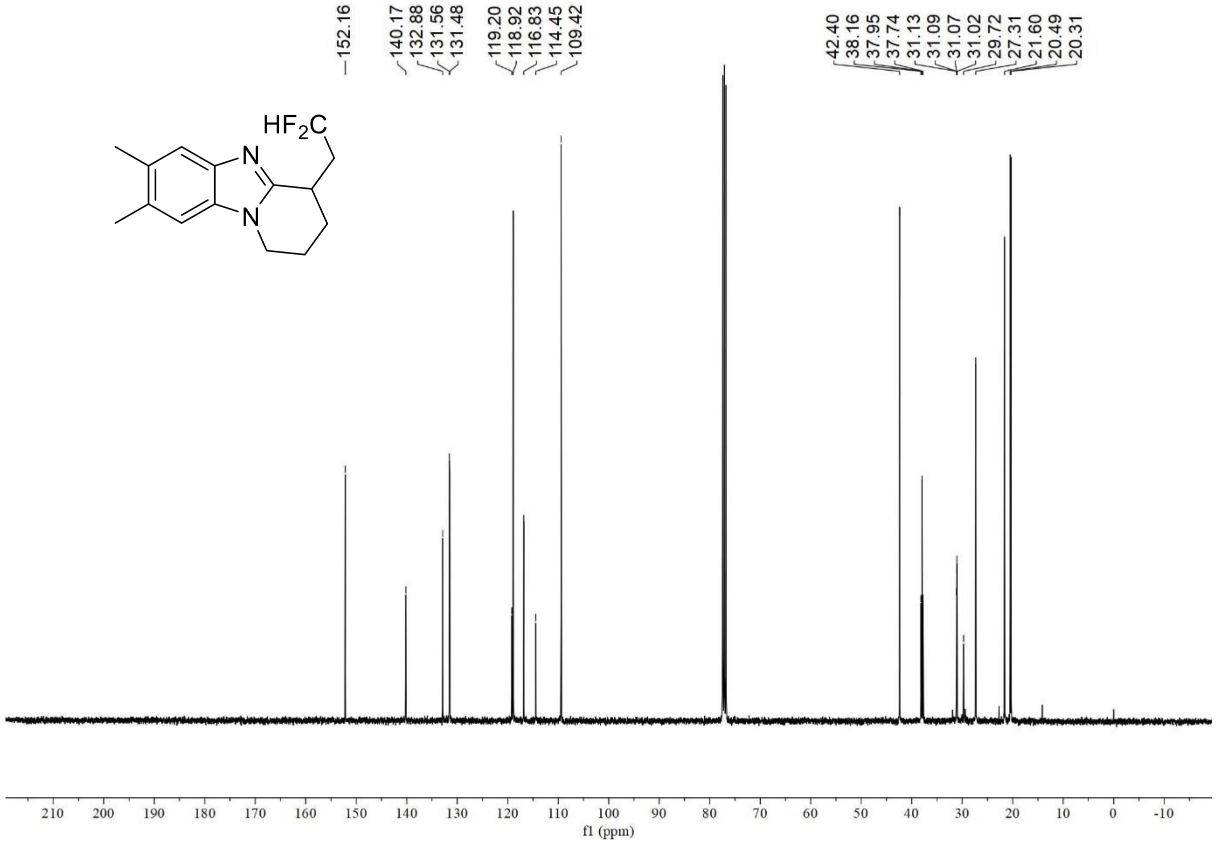

^{19}F｛H｝NMR (376 MHz, $CDCl_3$)

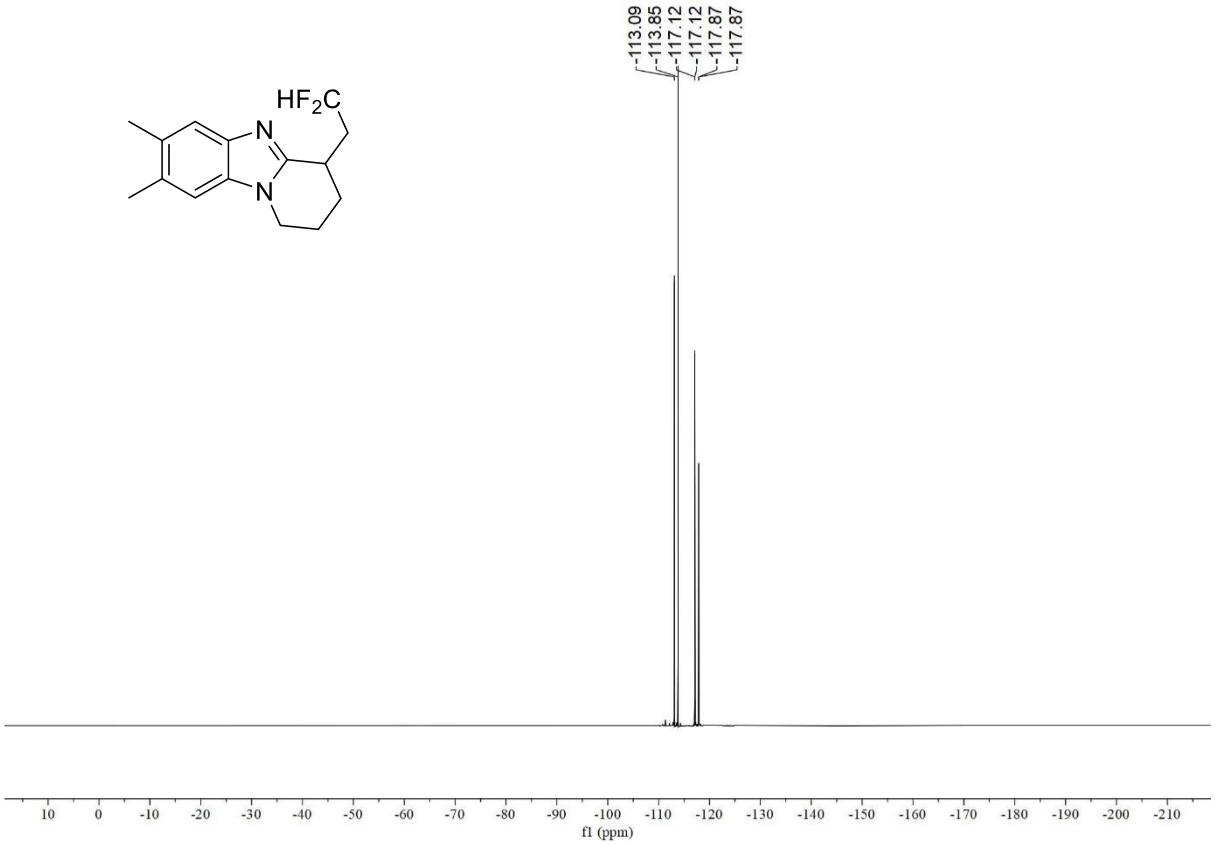

8-(2,2-Difluoroethyl)-2,3-diphenyl-5,6,7,8-tetrahydroimidazo[1,2-*a*]pyridine (3i)

^{1}H NMR (400 MHz, $CDCl_3$)

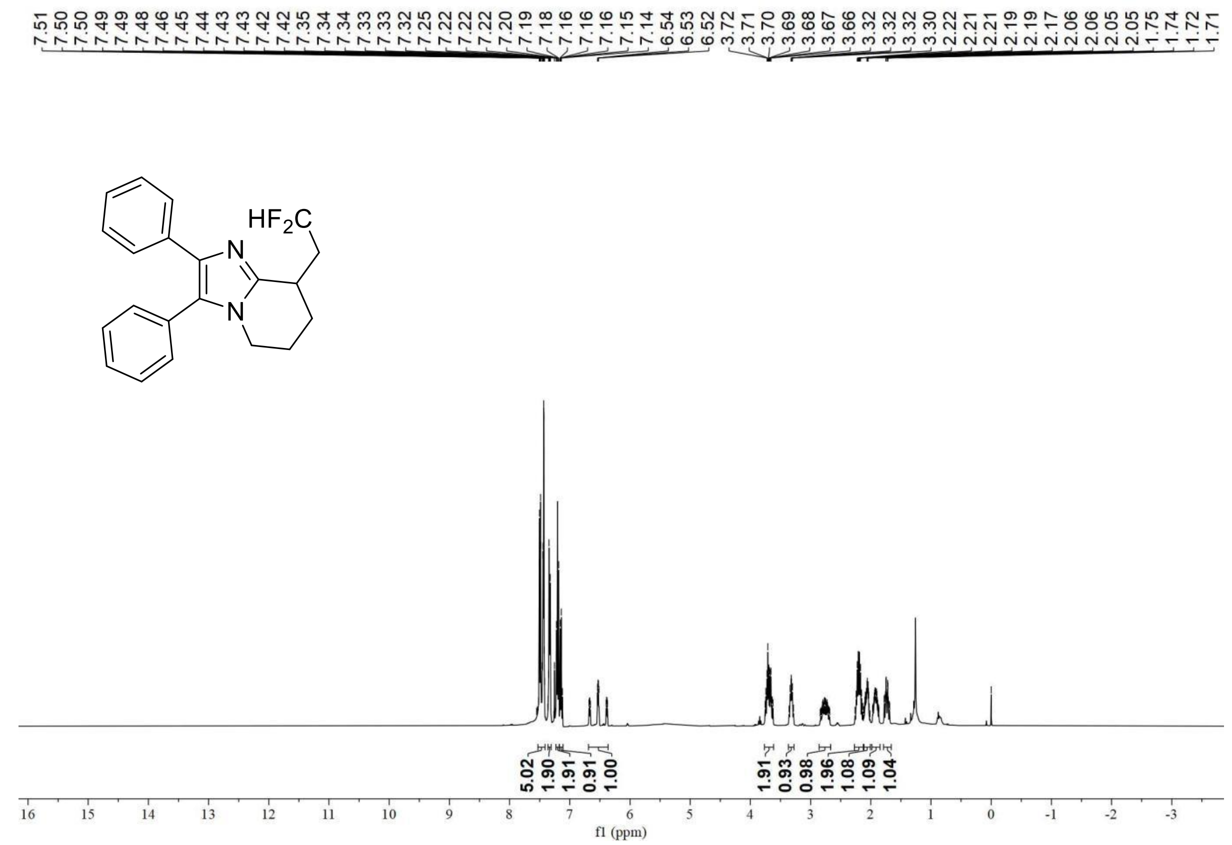

^{13}C NMR (100 MHz, $CDCl_3$)

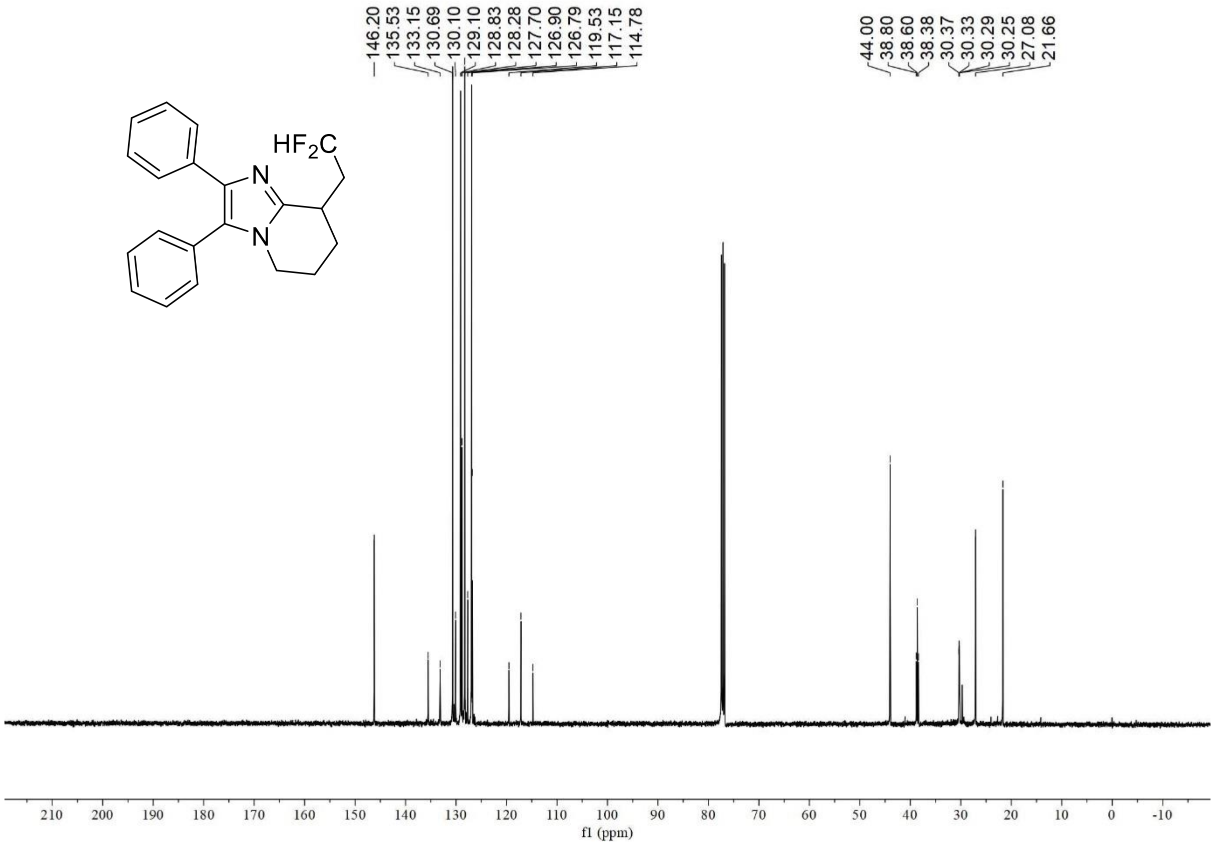

^{19}F {H} NMR (376 MHz, $CDCl_3$)

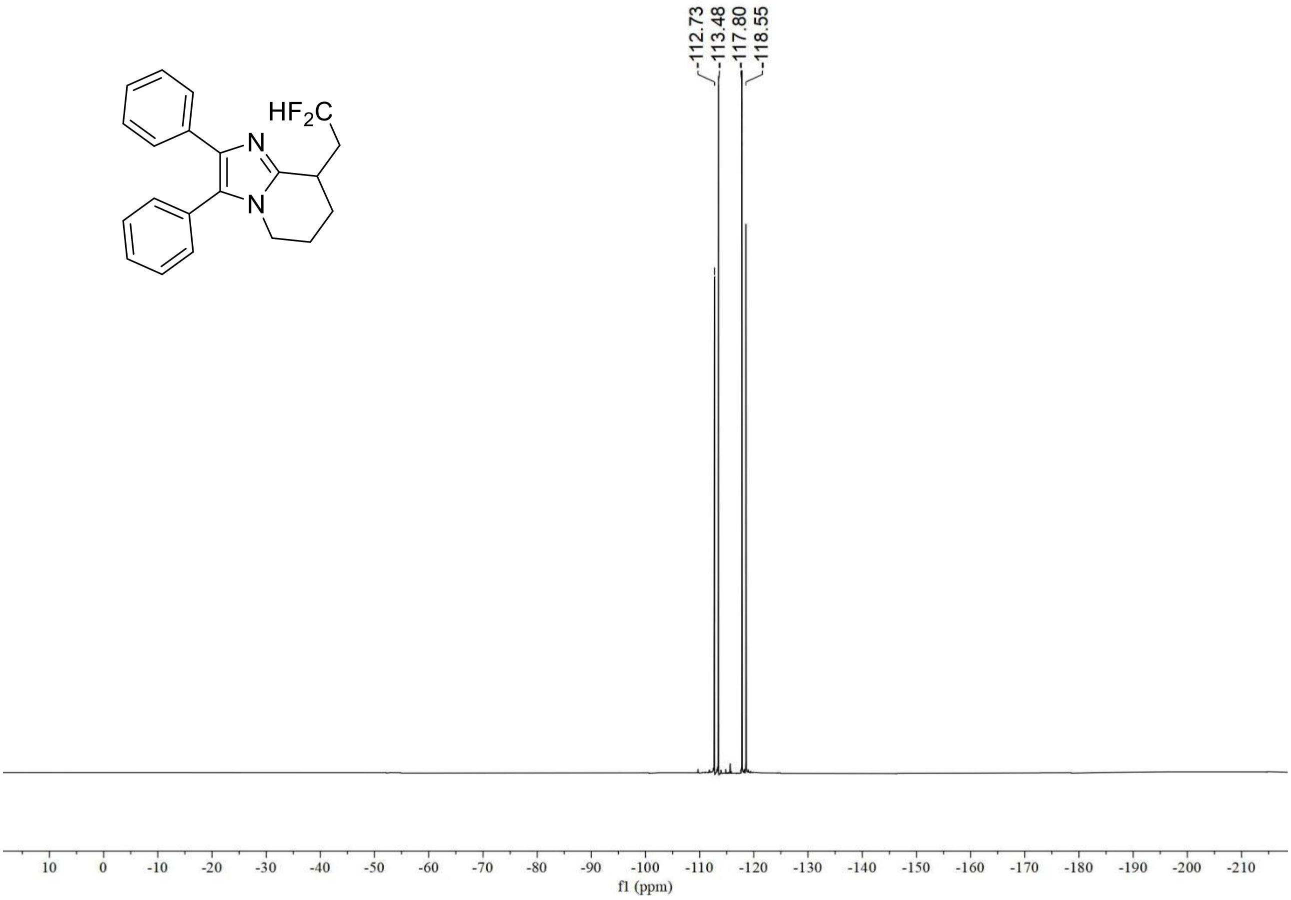

Dimethyl 8-(2,2-difluoroethyl)-5,6,7,8-tetrahydroimidazo[1,2-*a*]pyridine-2,3-dicarboxylate (3j)

^{1}H NMR (400 MHz, $CDCl_3$)

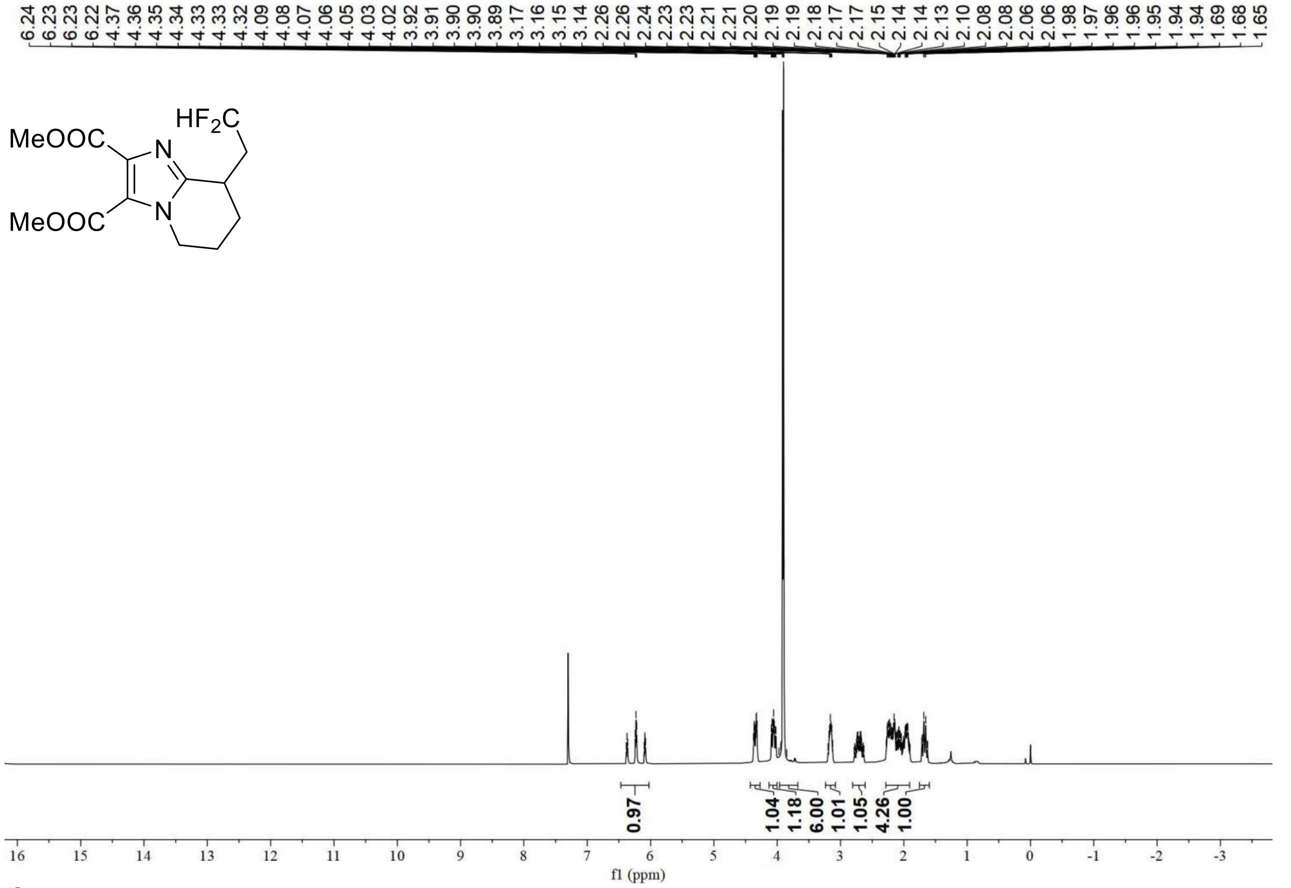

^{13}C NMR (100 MHz, $CDCl_3$)

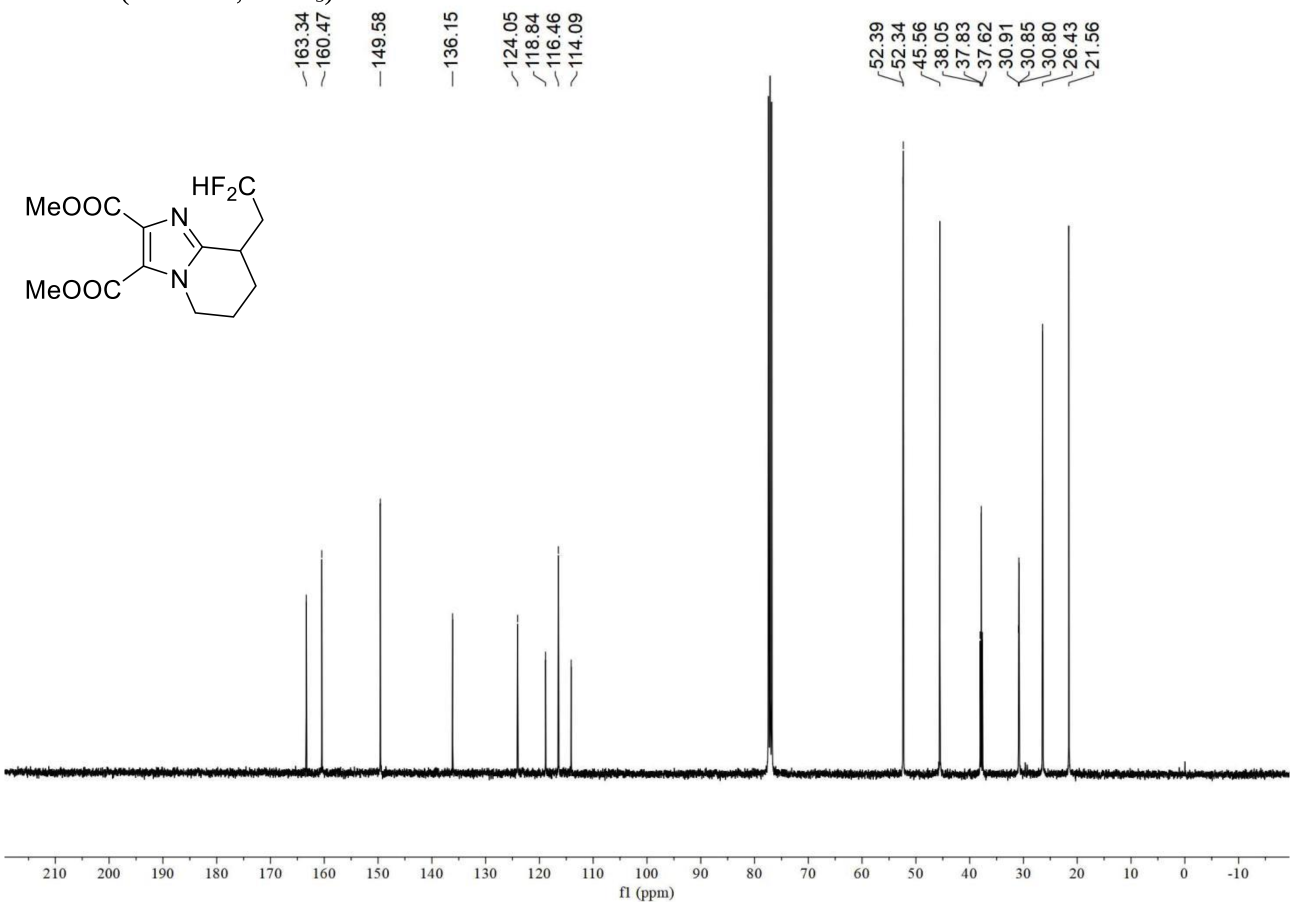

^{19}F {H} NMR (376 MHz, $CDCl_3$)

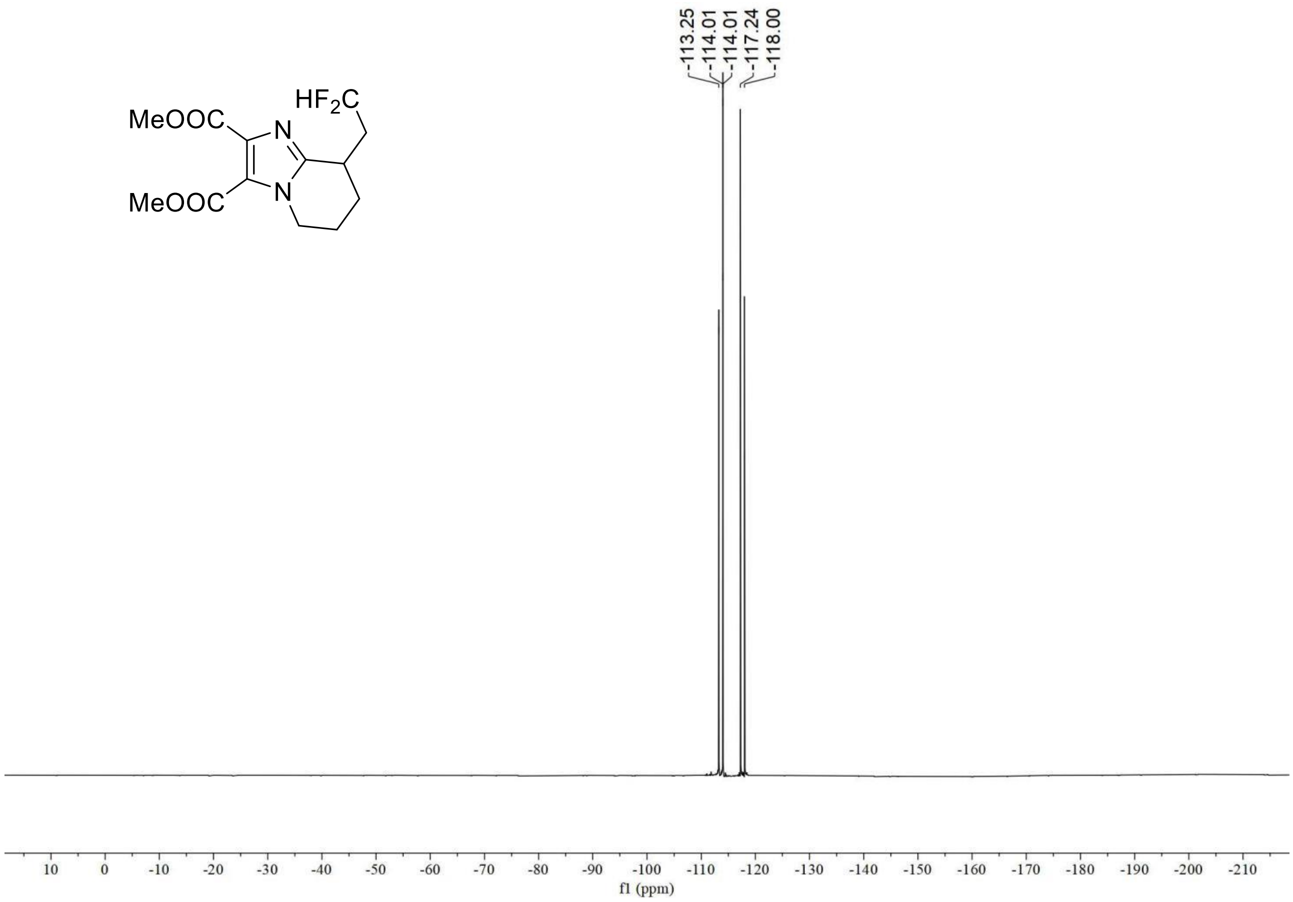

Methyl 8-(2,2-difluoroethyl)-5,6,7,8-tetrahydroimidazo[1,2-*a*]pyridine-3-carboxylate (3k)

^{1}H NMR (400 MHz, $CDCl_3$)

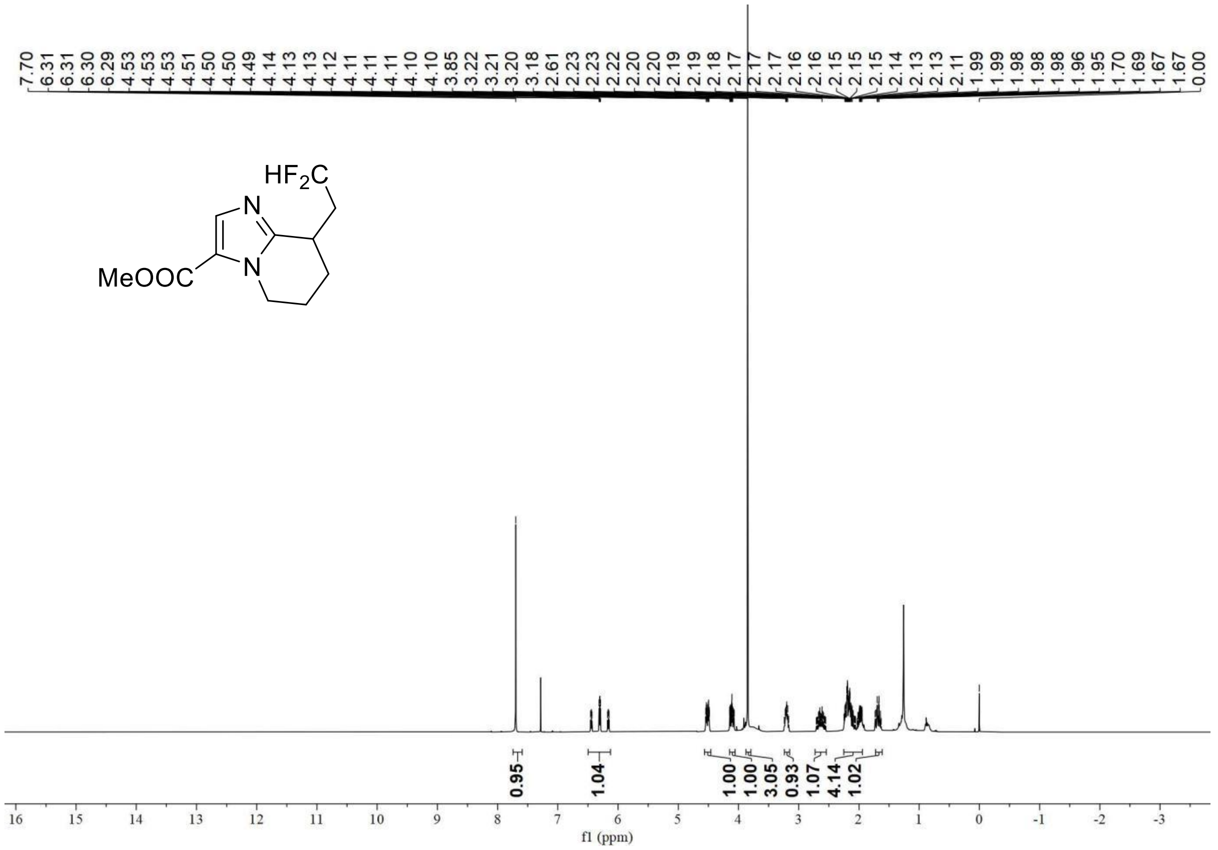

^{13}C NMR (100 MHz, $CDCl_3$)

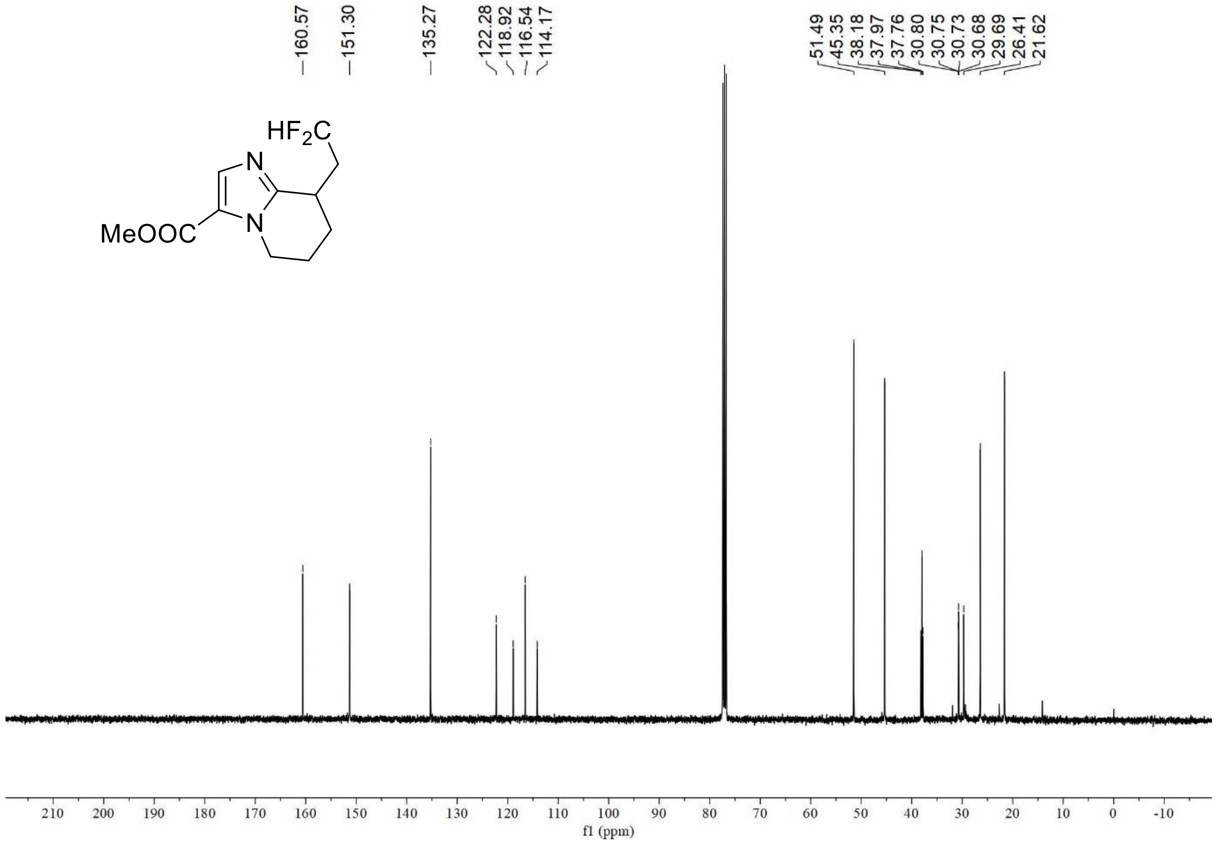

$^{19}F\{H\}$ NMR (376 MHz, $CDCl_3$)

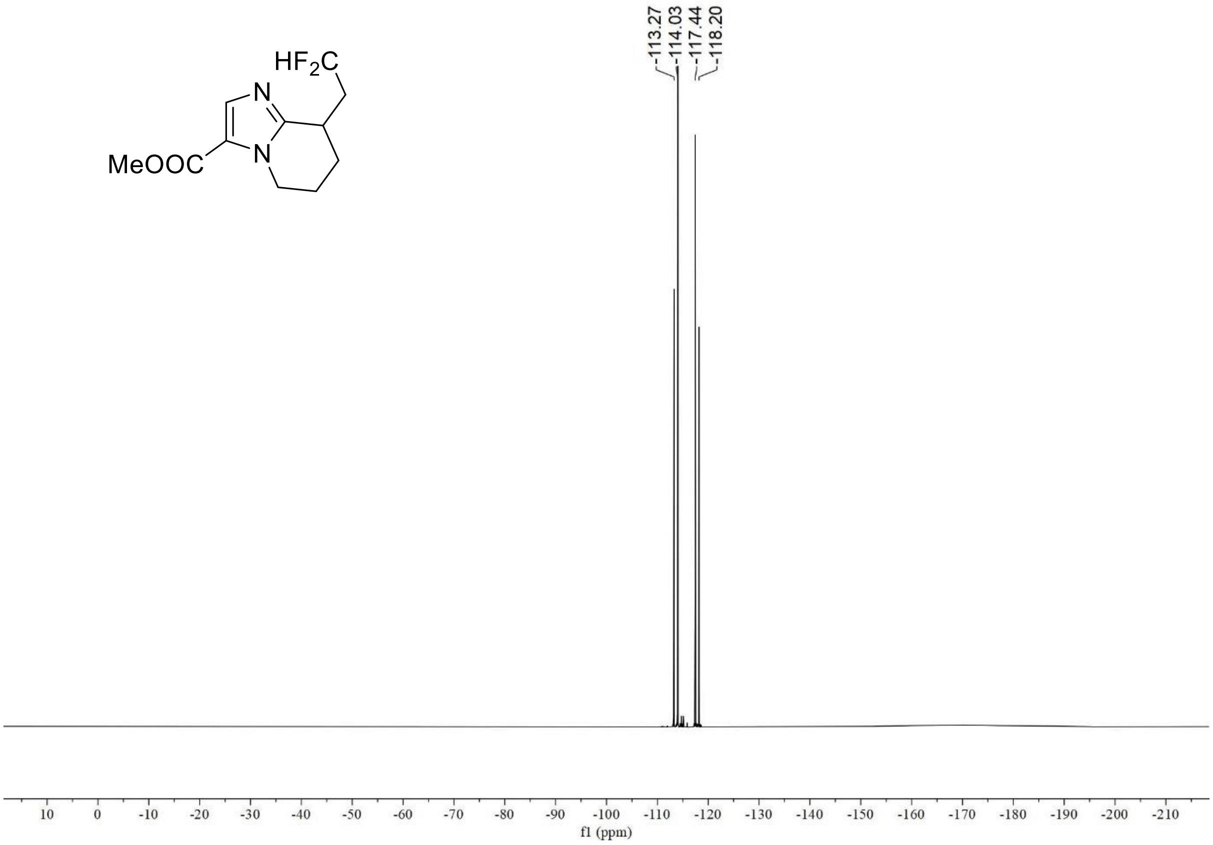

3-(2,2-Difluoroethyl)-2,3-dihydro-1*H*-benzo[*d*]pyrrolo[1,2-*a*]imidazole (3l)

^{1}H NMR (400 MHz, $CDCl_3$)

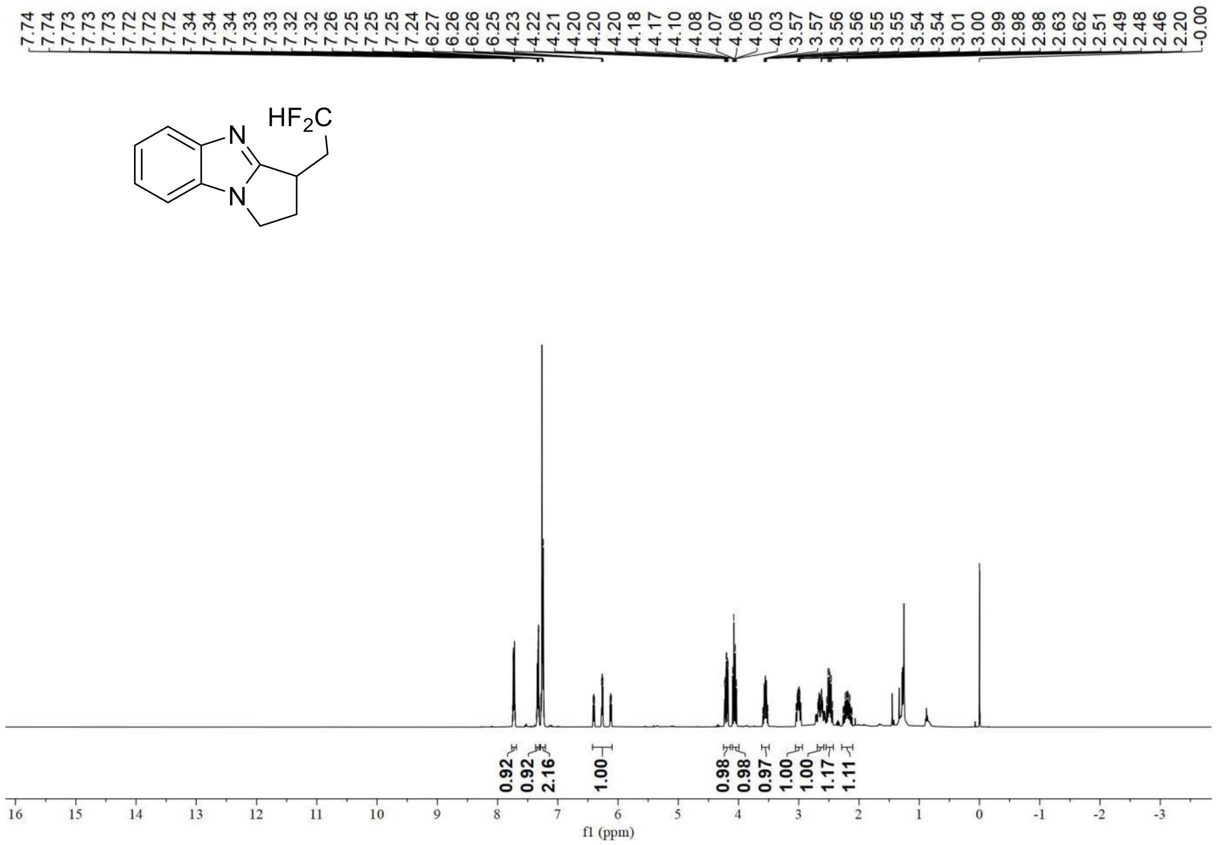

^{13}C NMR (100 MHz, $CDCl_3$)

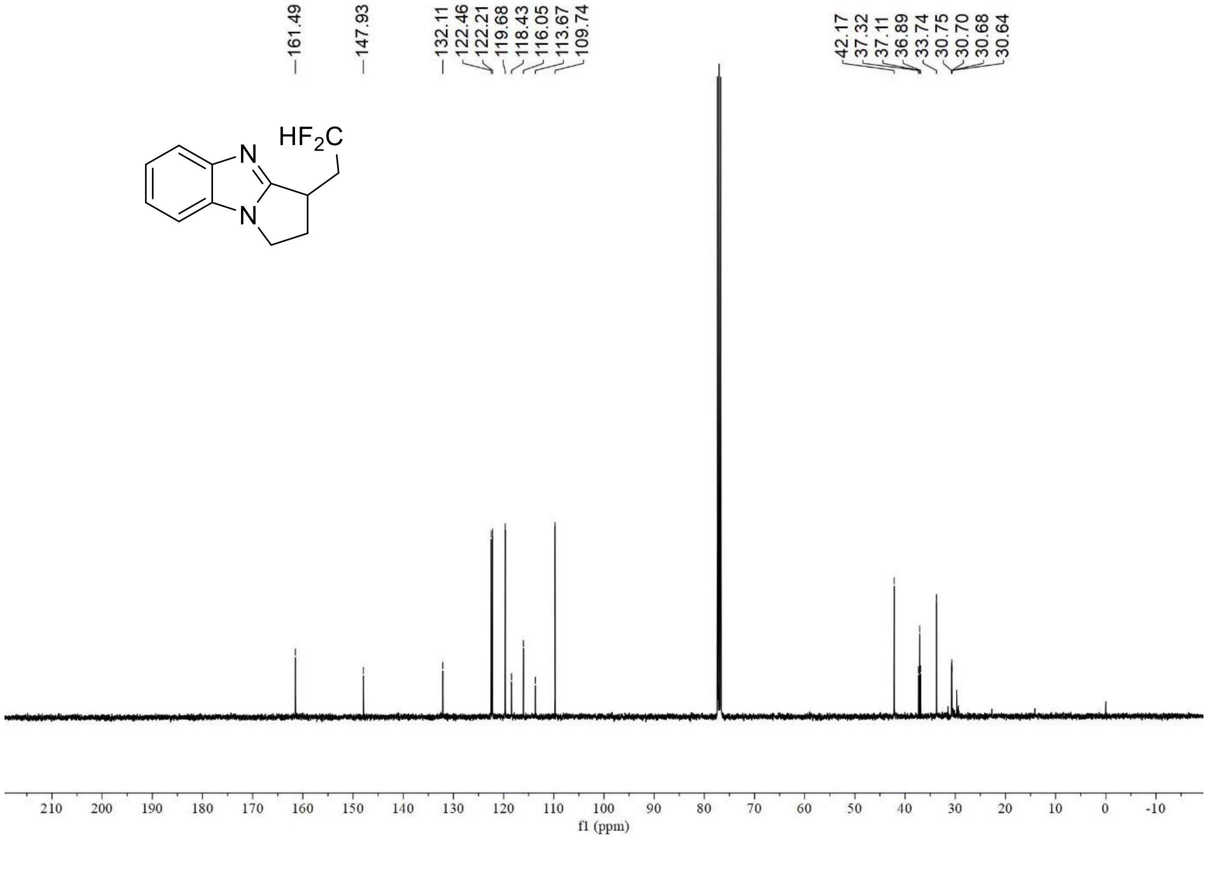

$^{19}F\{H\}$ NMR (376 MHz, $CDCl_3$)

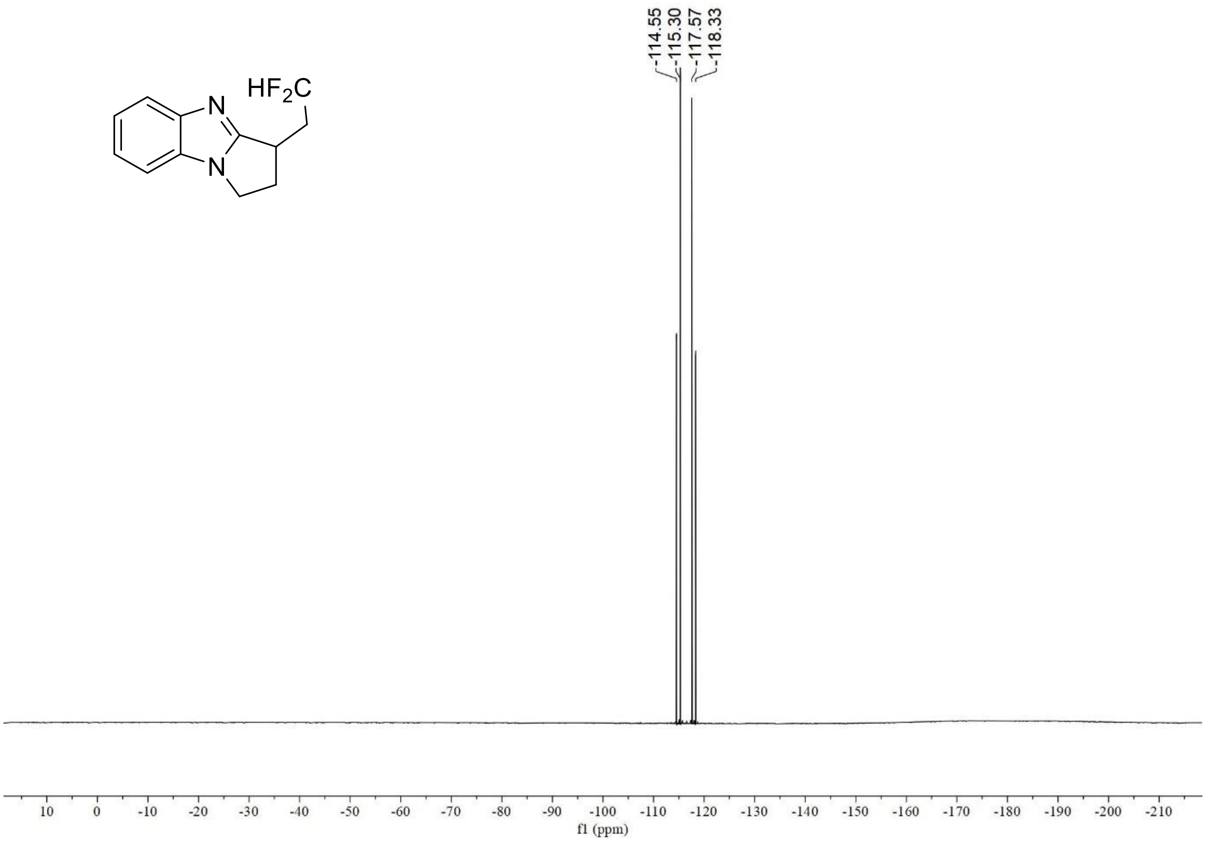

3-(2,2-Difluoroethyl)-8-methyl-2,3-dihydro-1*H*-benzo[*d*]pyrrolo[1,2-*a*]imidazole (3m)

^{1}H NMR (400 MHz, $CDCl_3$)

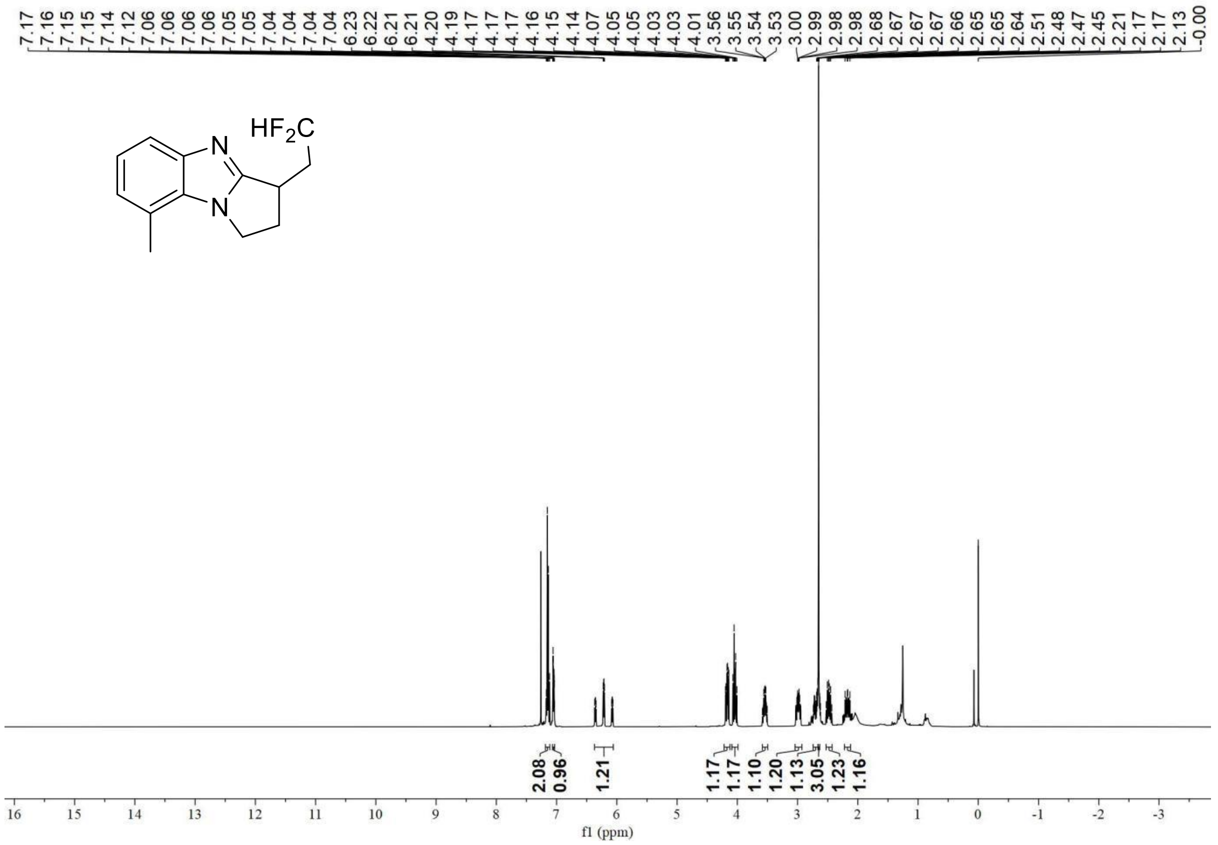

^{13}C NMR (100 MHz, $CDCl_3$)

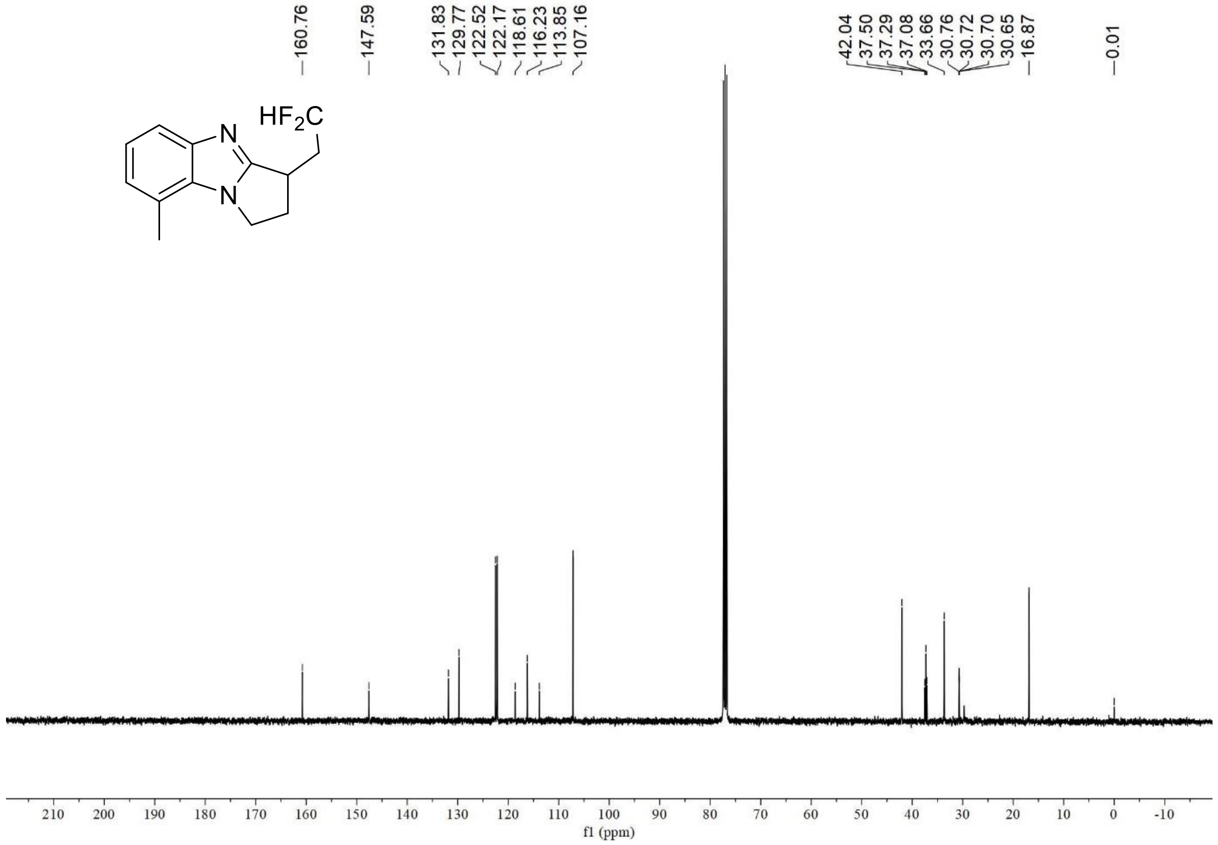

$^{19}F\{H\}$ NMR (376 MHz, $CDCl_3$)

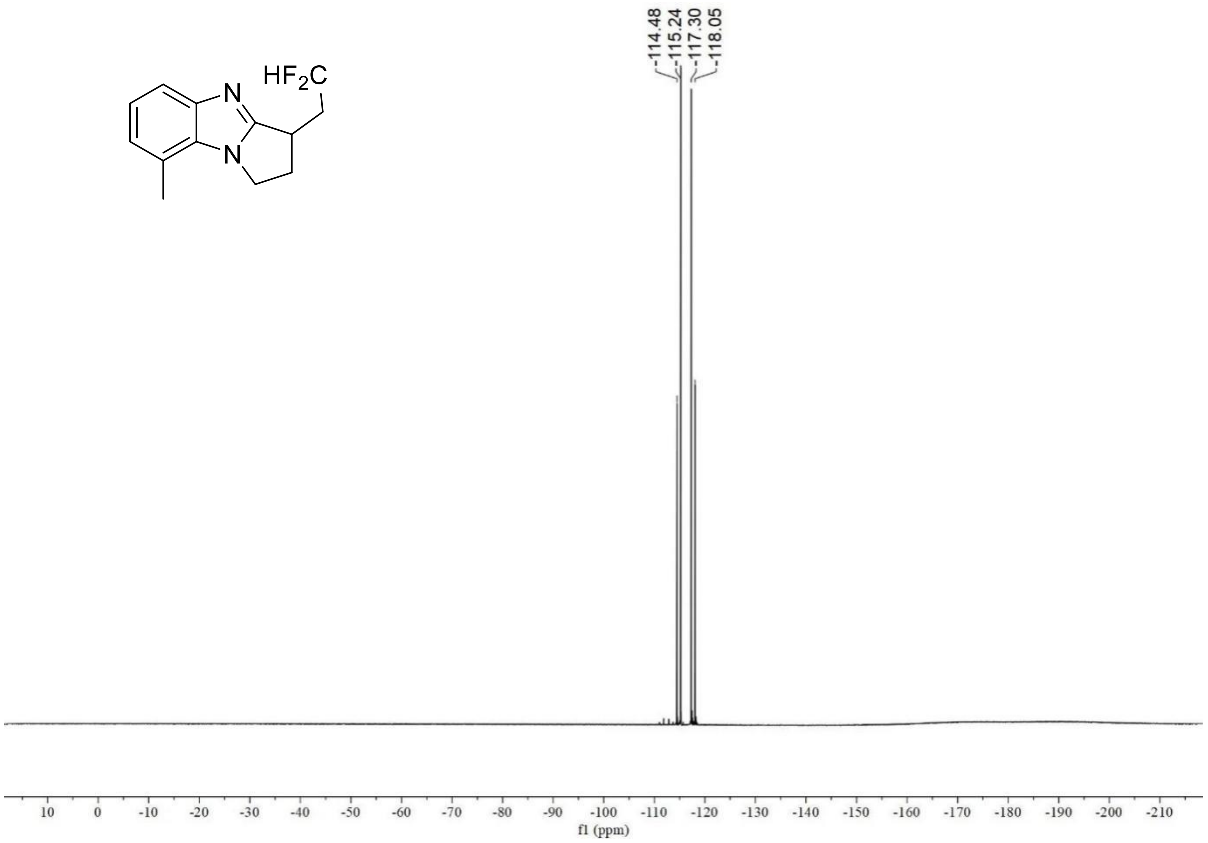

6,7-Dichloro-3-(2,2-difluoroethyl)-2,3-dihydro-1*H*-benzo[*d*]pyrrolo[1,2-*a*]imidazole (3n)

^{1}H NMR (400 MHz, $CDCl_3$)

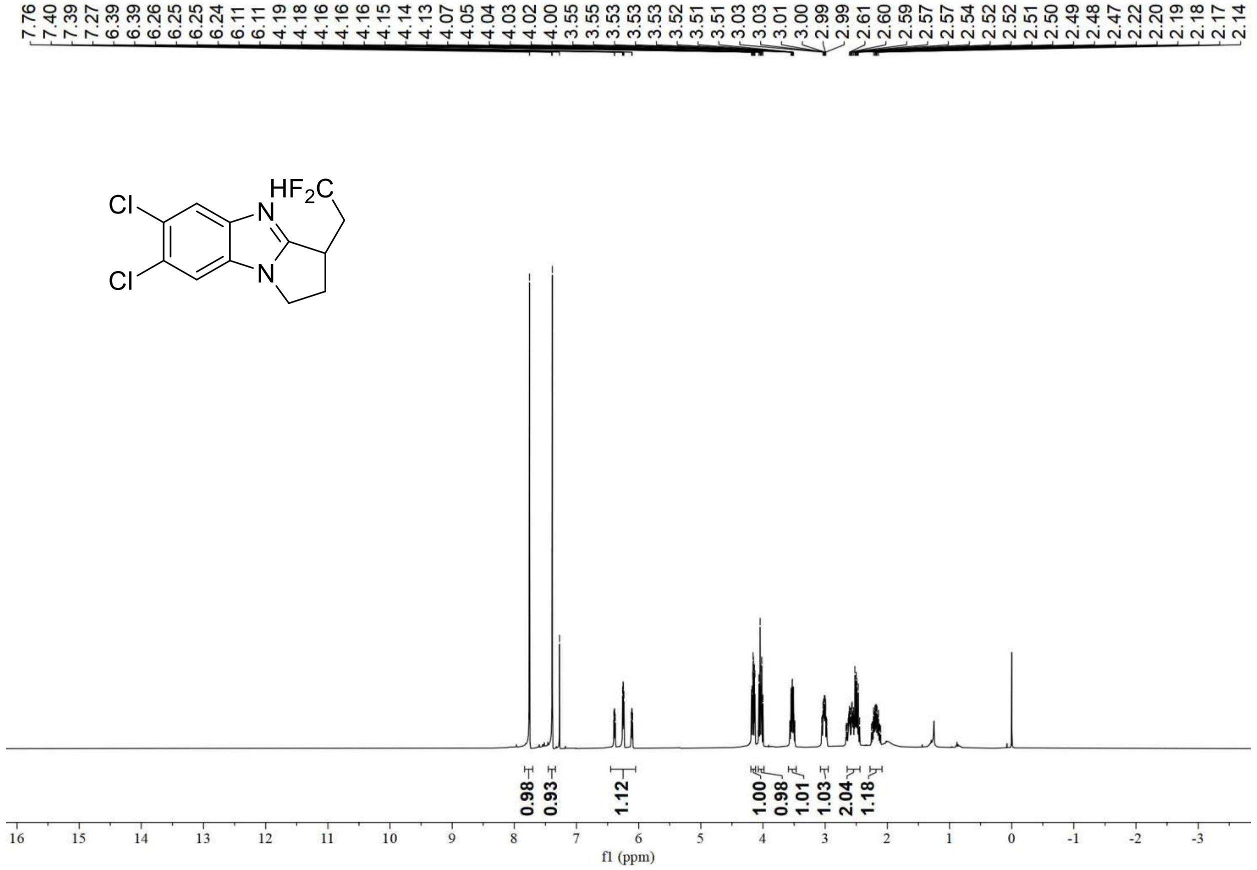

^{13}C NMR (100 MHz, $CDCl_3$)

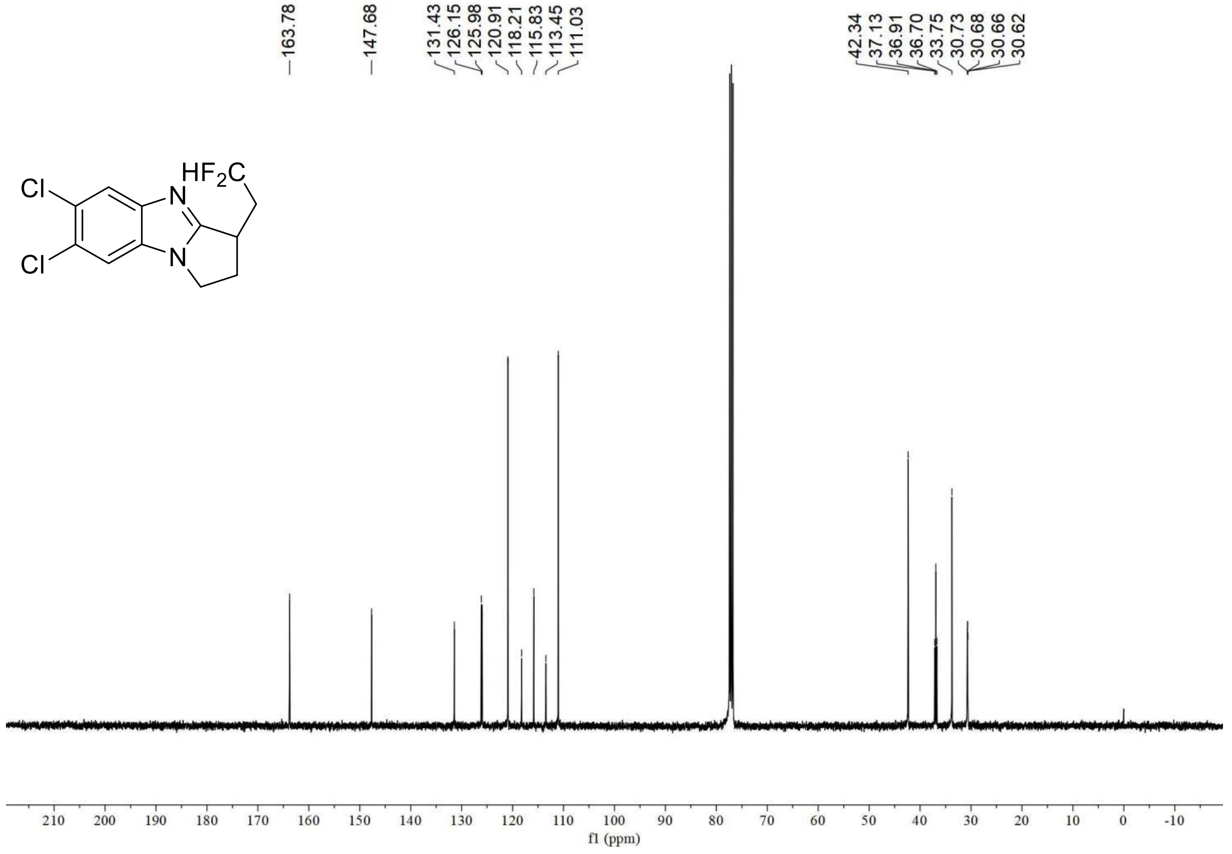

^{19}F｛H｝NMR (376 MHz, $CDCl_3$)

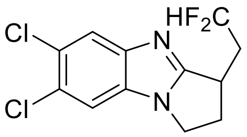

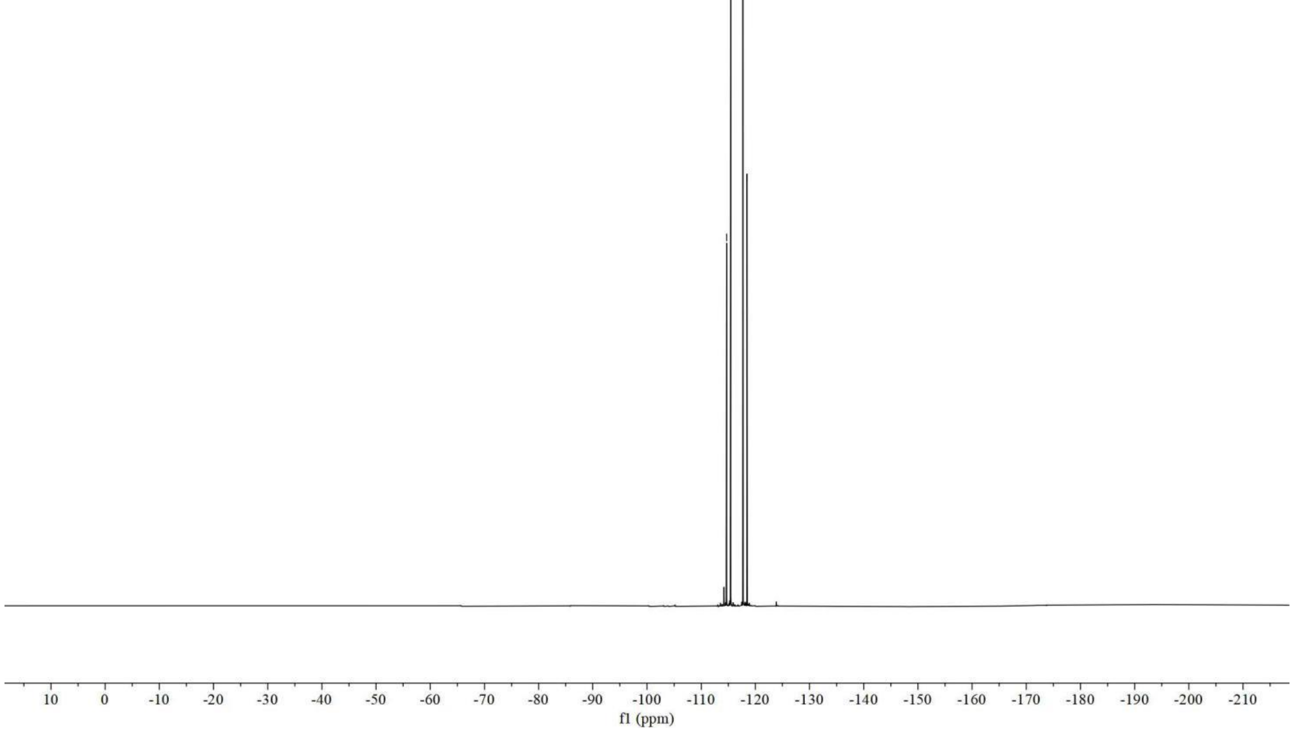

6-(2,2-Difluoroethyl)-7,8,9,10-tetrahydro-6*H*-benzo[4,5]imidazo[1,2-*a*]azepine (3o)

^{1}H NMR (400 MHz, $CDCl_3$)

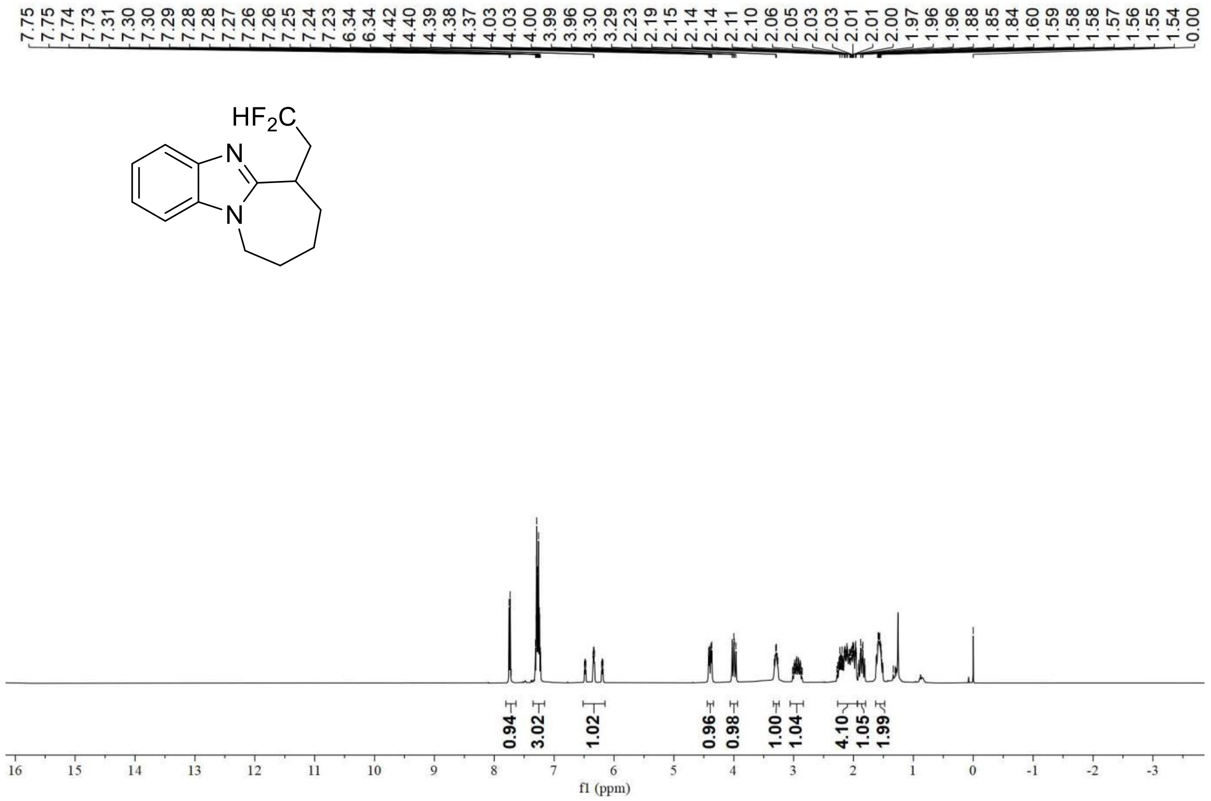

^{13}C NMR (100 MHz, $CDCl_3$)

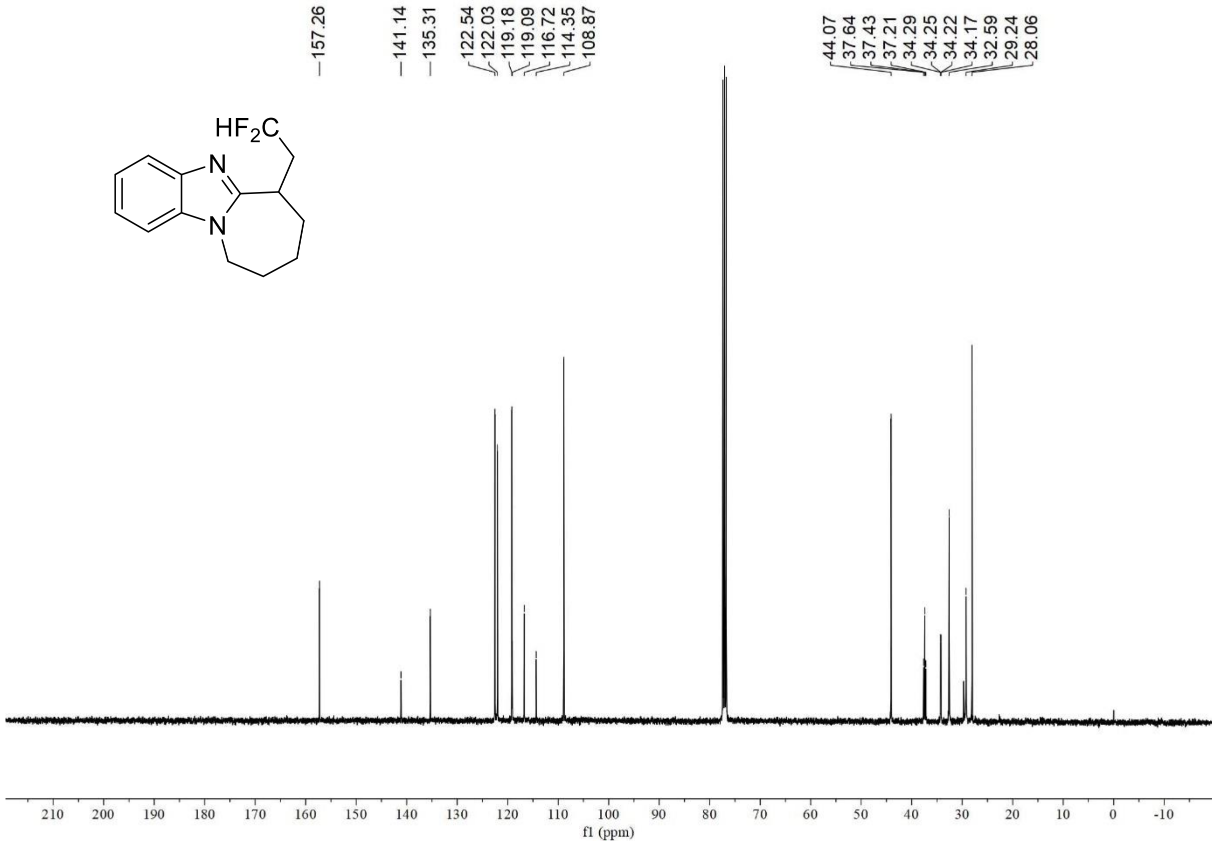

$^{19}F\{H\}$ NMR (376 MHz, $CDCl_3$)

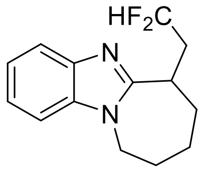

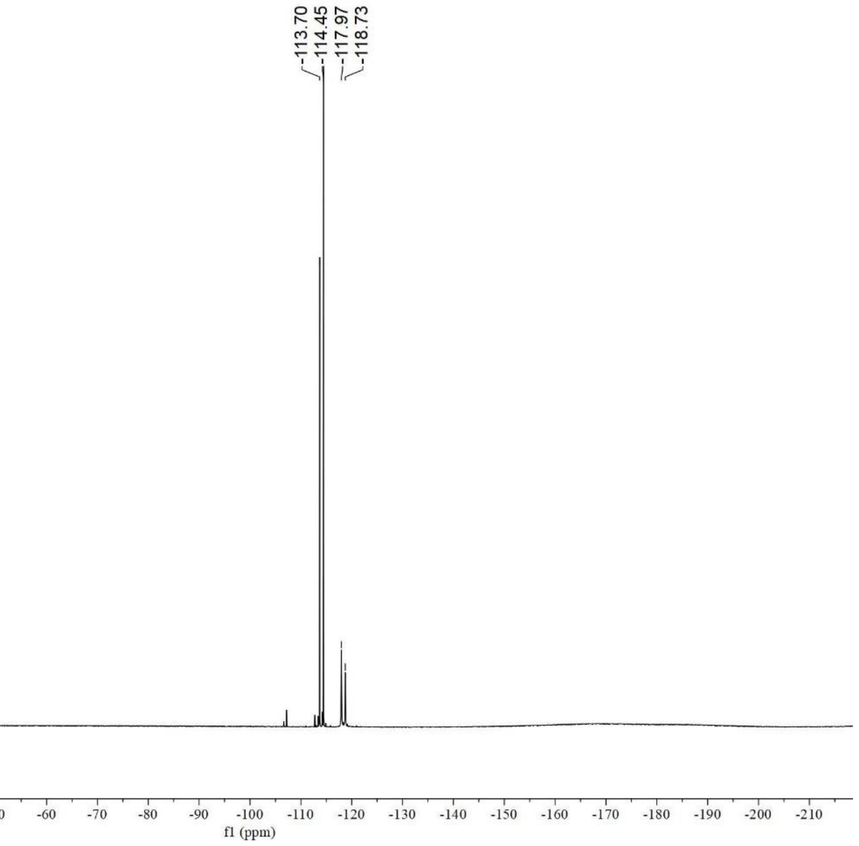

6-(2,2-Difluoroethyl)-1-methyl-7,8,9,10-tetrahydro-6*H*-benzo[4,5]imidazo[1,2-*a*]azepine (3p)

^{1}H NMR (400 MHz, $CDCl_3$)

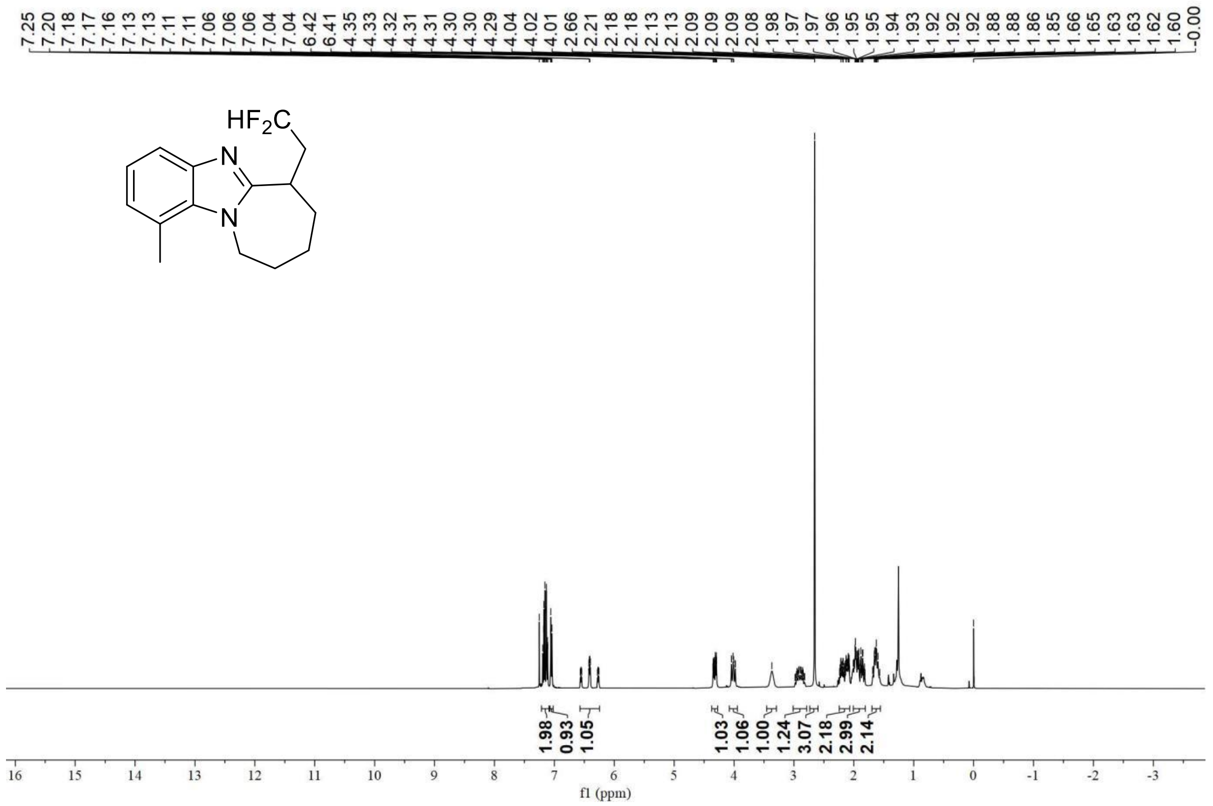

^{13}C NMR (100 MHz, $CDCl_3$)

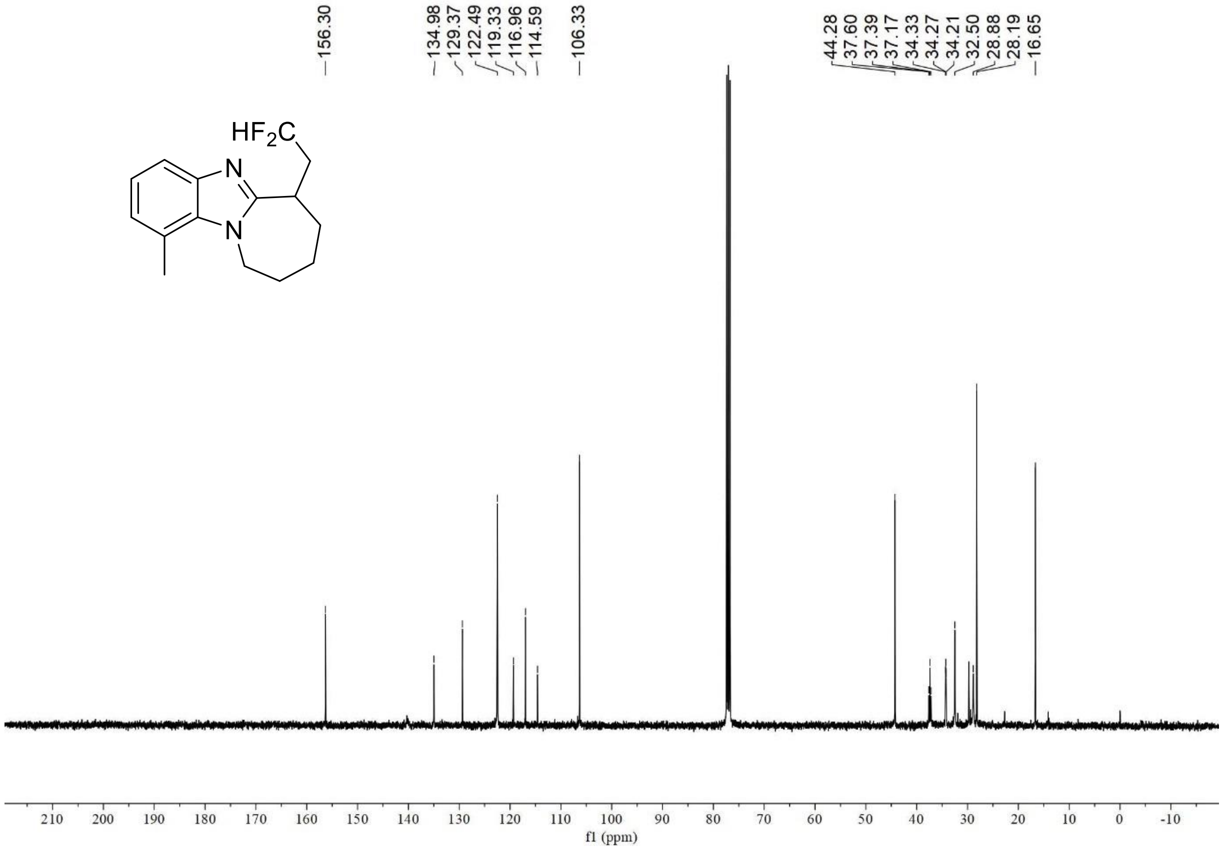

$^{19}F\{H\}$ NMR (376 MHz, $CDCl_3$)

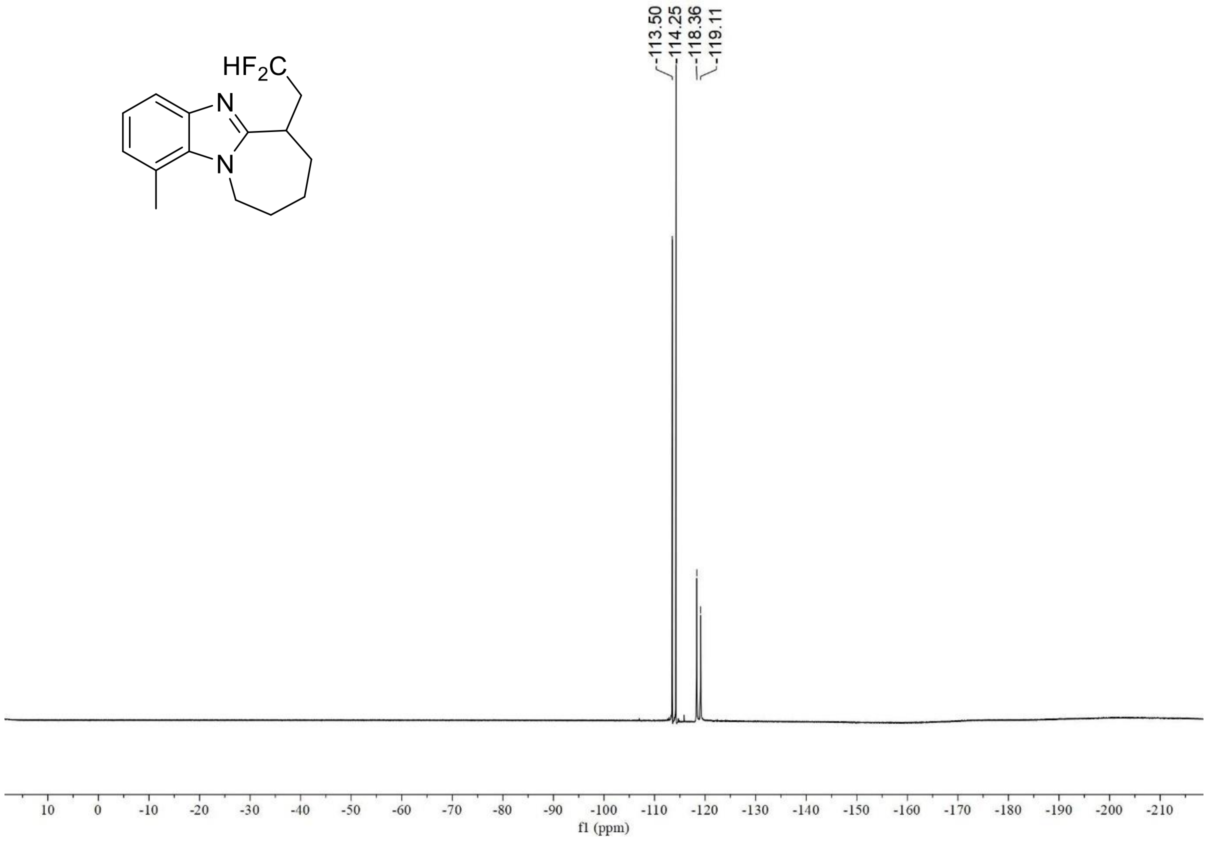

5-(2,2-Difluoroethyl)-5,6-dihydrobenzo[4,5]imidazo[2,1-*a*]isoquinoline (3q)

^{1}H NMR (400 MHz, $CDCl_3$)

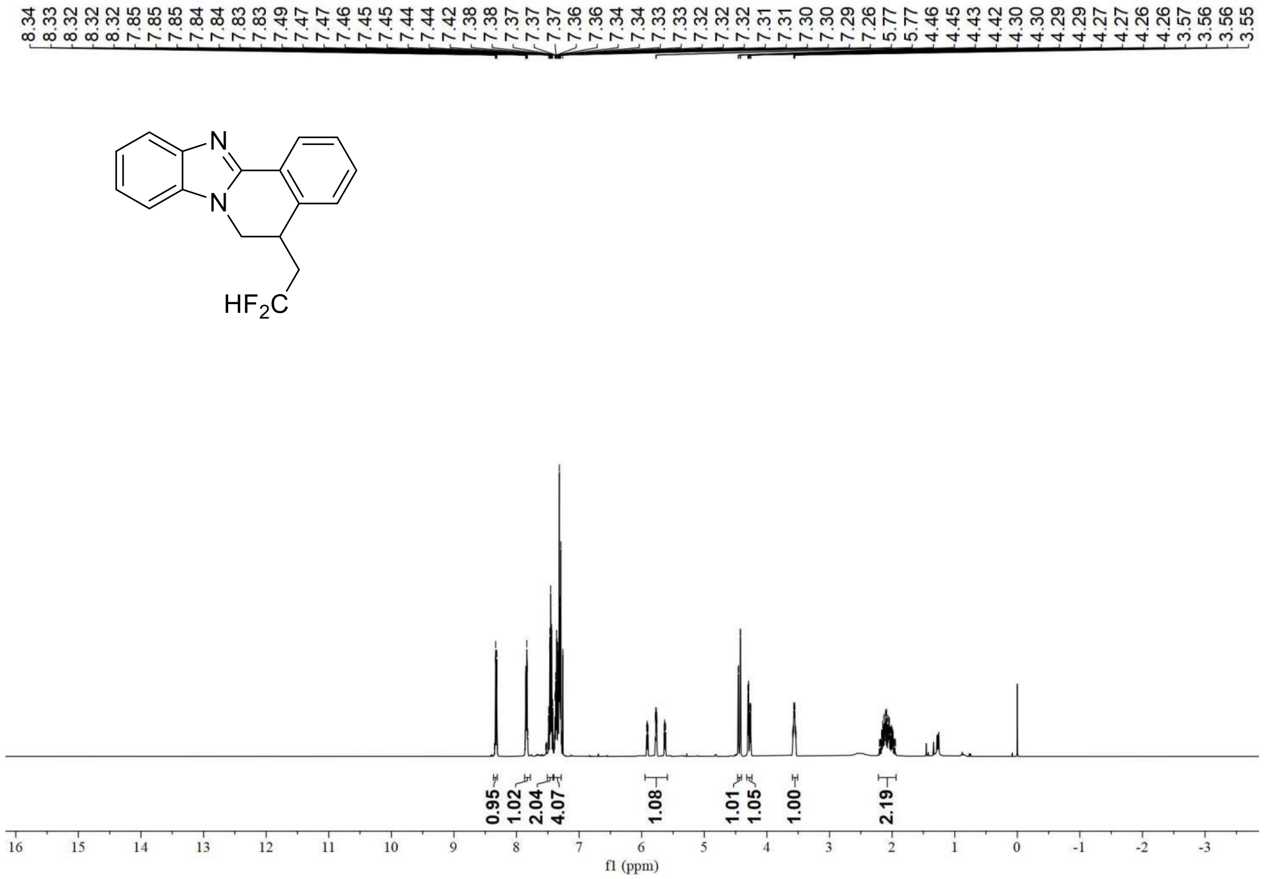

^{13}C NMR (100 MHz, $CDCl_3$)

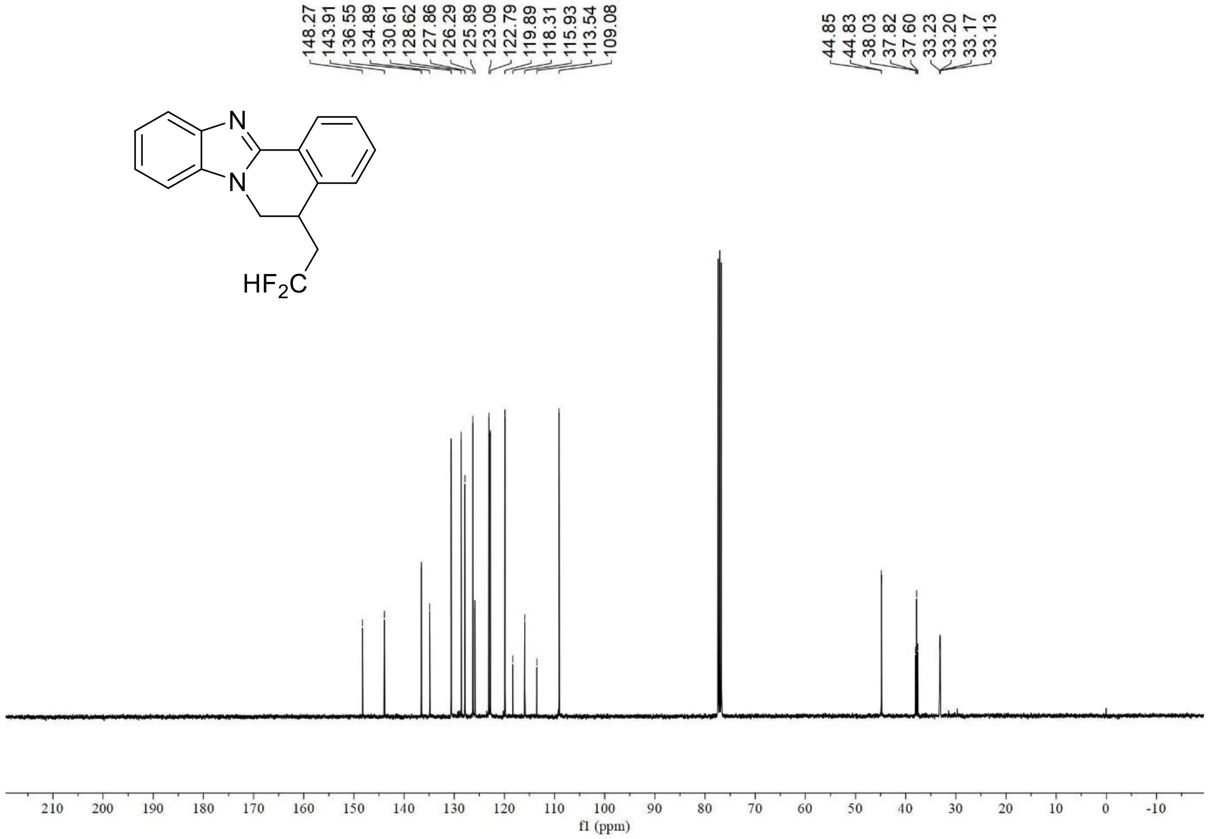

^{19}F {H} NMR (376 MHz, $CDCl_3$)

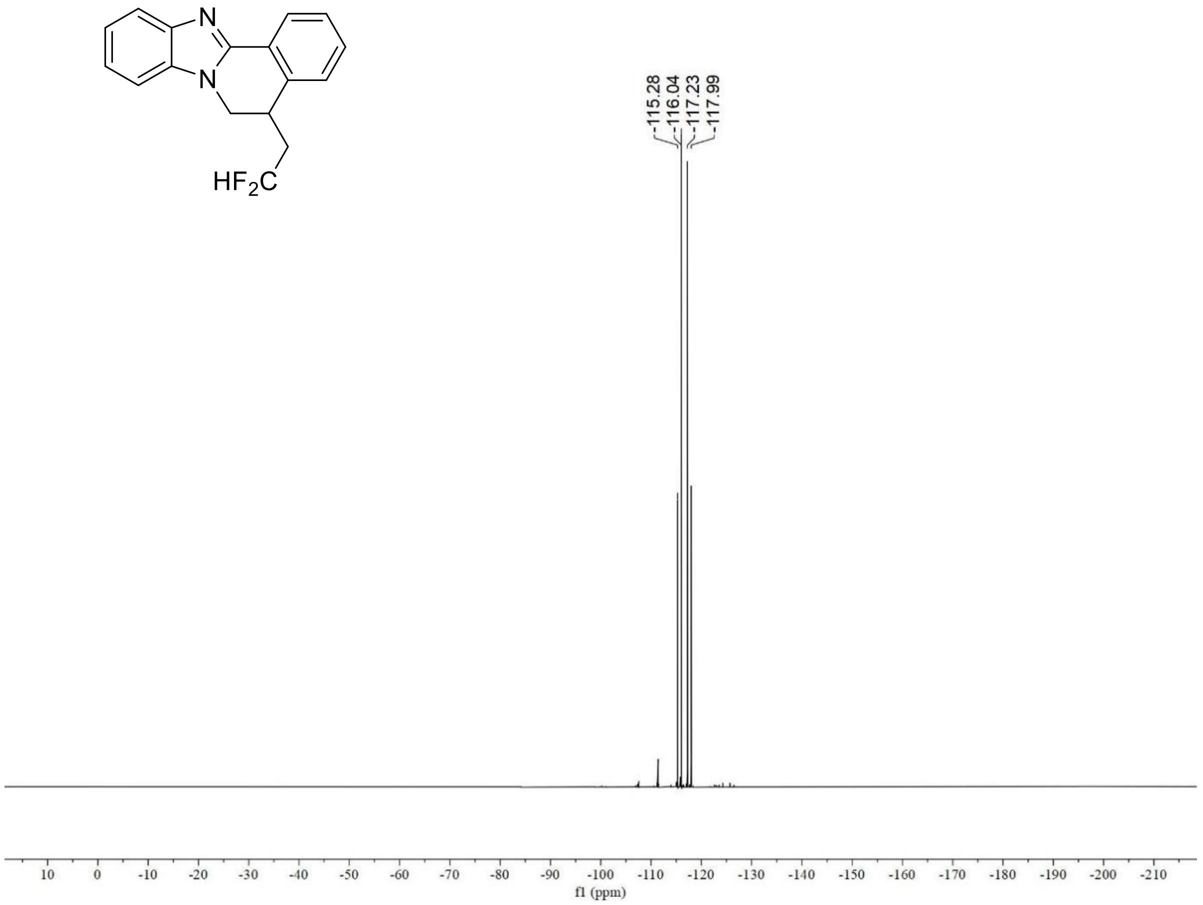

4-(2,2-Difluoro-2-phenylethyl)-1,2,3,4-tetrahydrobenzo[4,5]imidazo[1,2-*a*]pyridine (3r)

^{1}H NMR (400 MHz, $CDCl_3$)

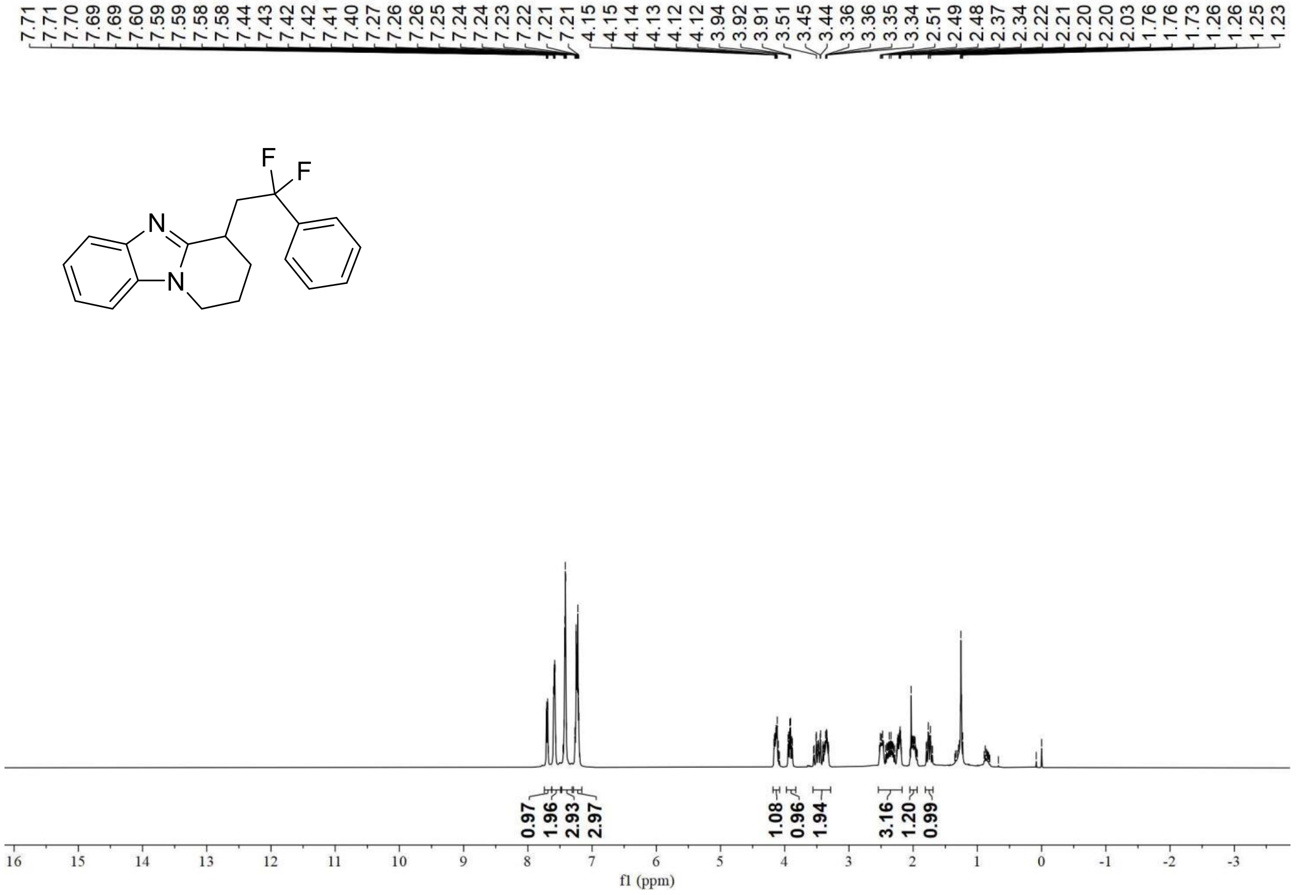

^{13}C NMR (100 MHz, $CDCl_3$)

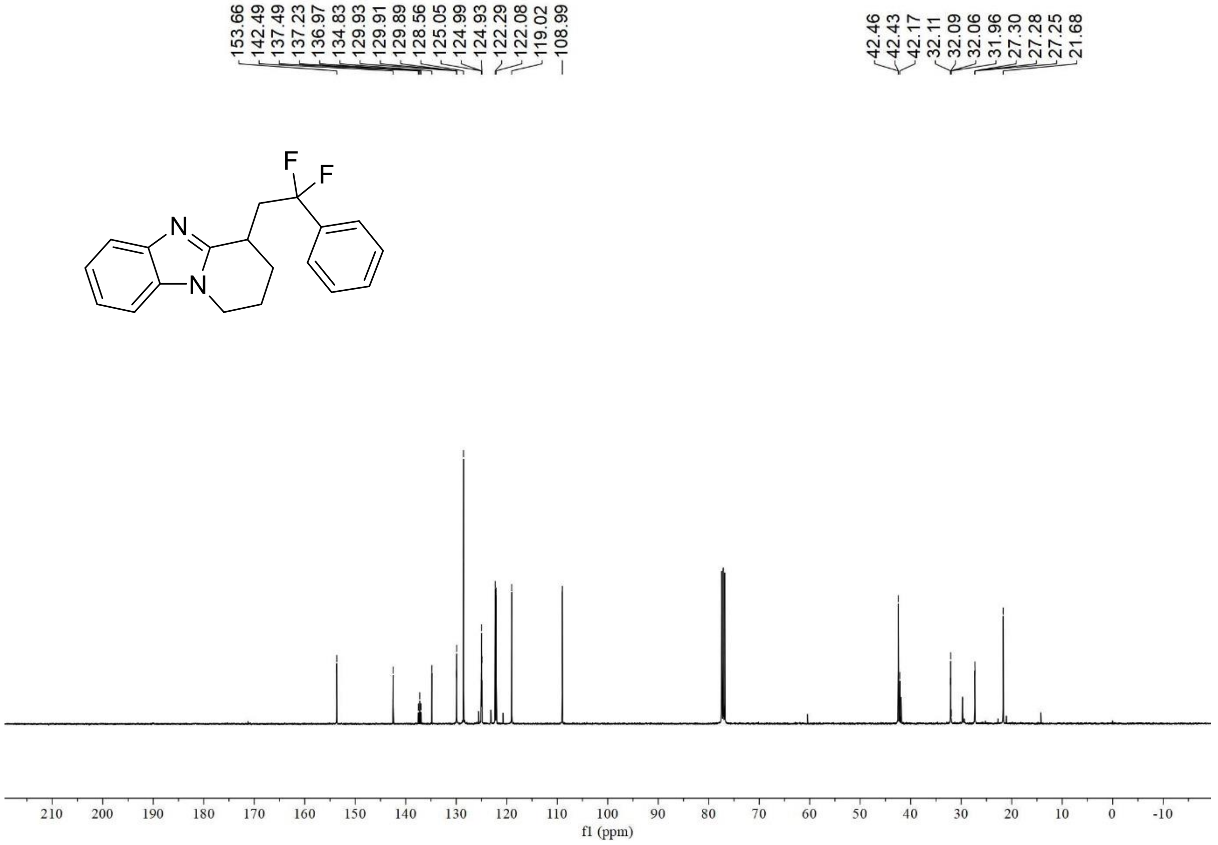

^{19}F {H} NMR (376 MHz, $CDCl_3$)

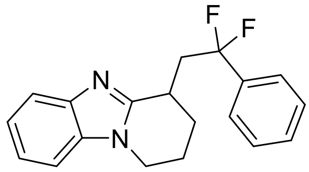

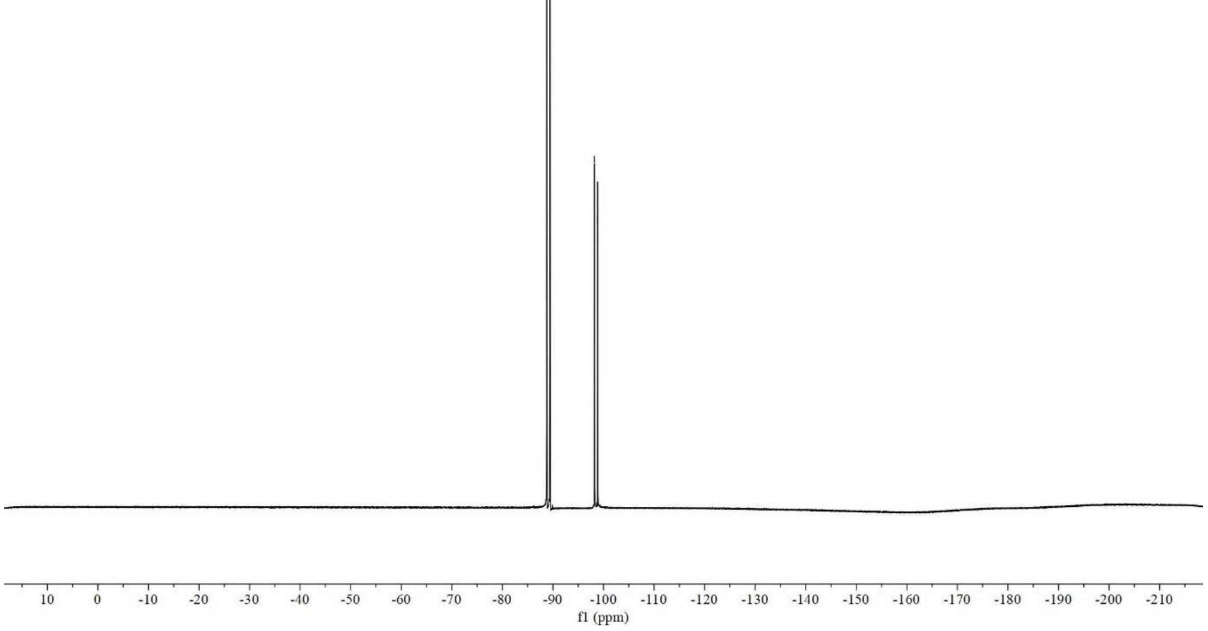

6-Bromo-4-(2,2-difluoro-2-phenylethyl)-1,2,3,4-tetrahydrobenzo[4,5]imidazo[1,2-*a*]pyridine (3s)

^{1}H NMR (400 MHz, $CDCl_3$)

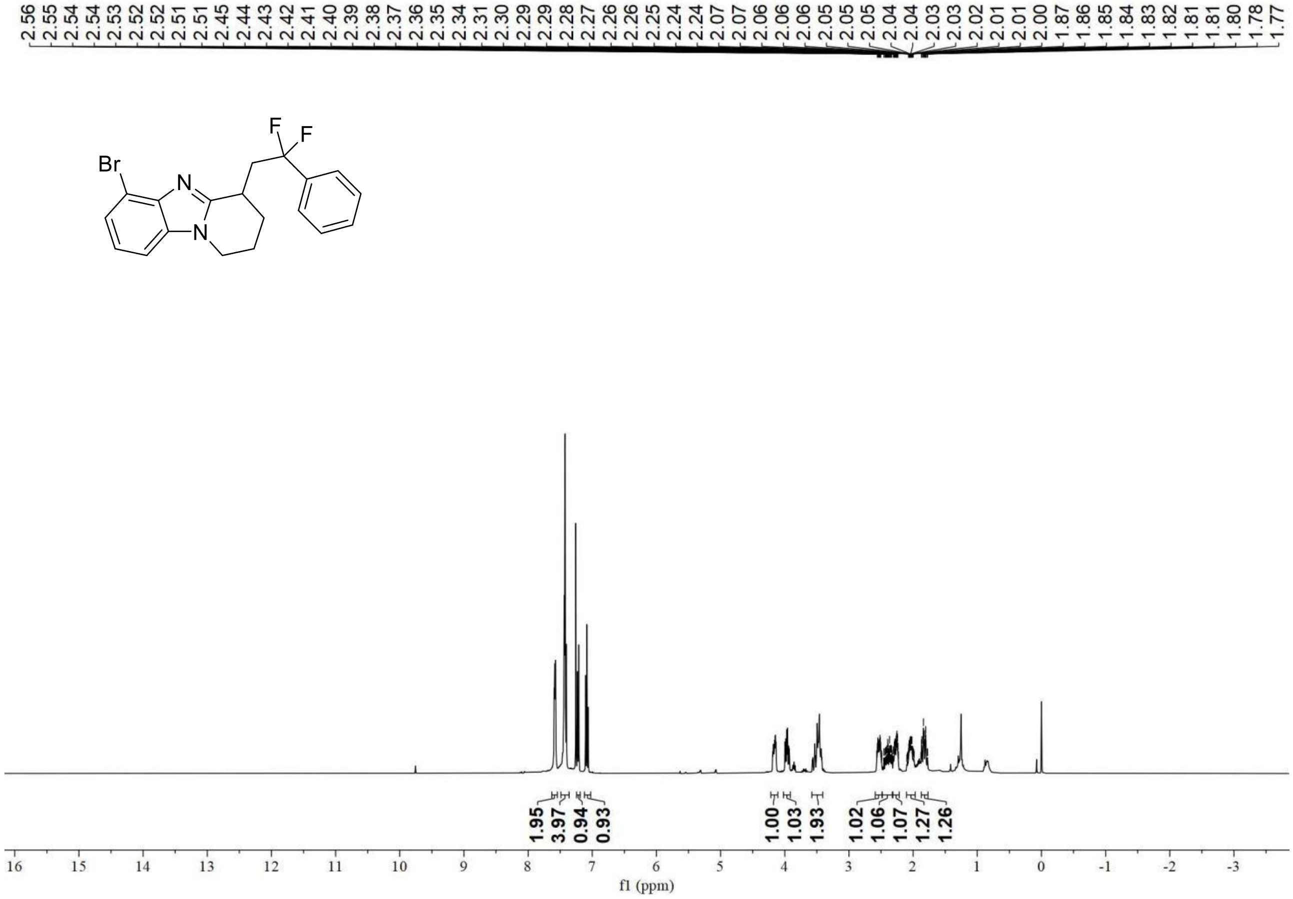

^{13}C NMR (100 MHz, $CDCl_3$)

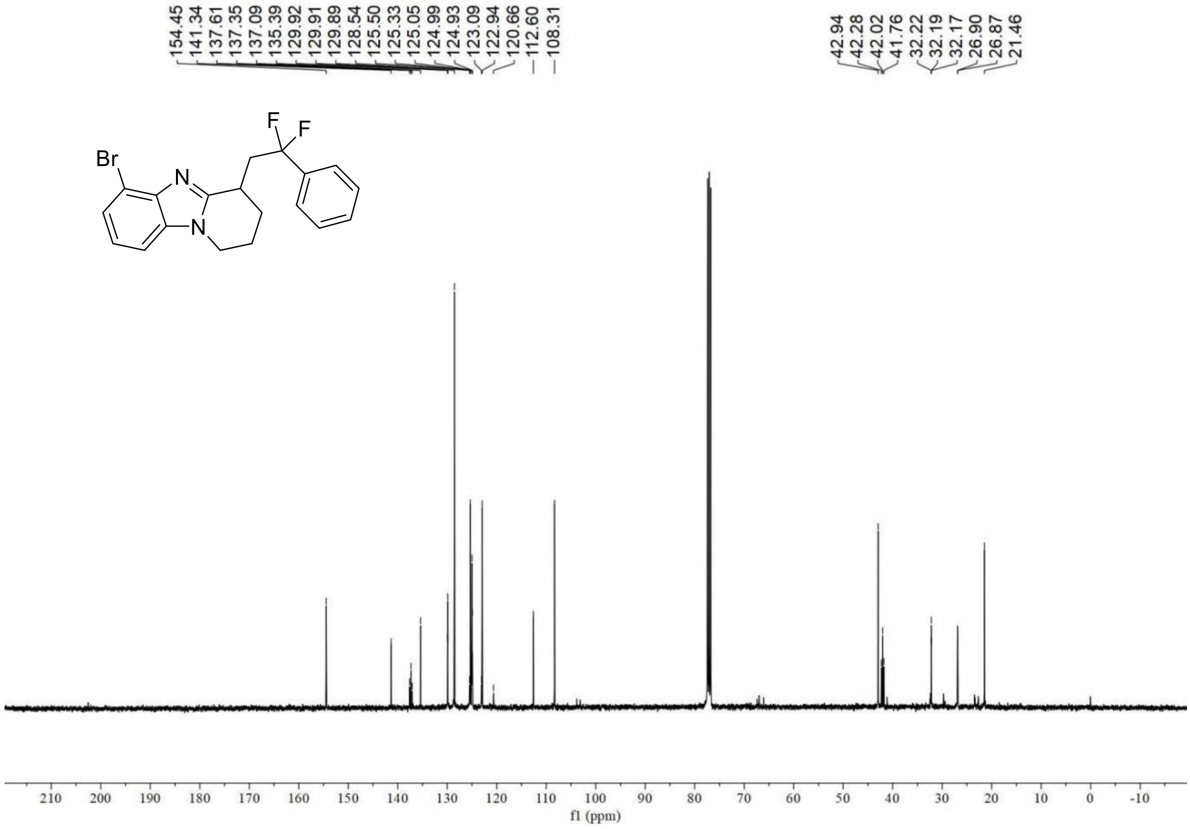

$^{19}F\{H\}$ NMR (376 MHz, $CDCl_3$)

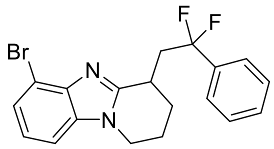

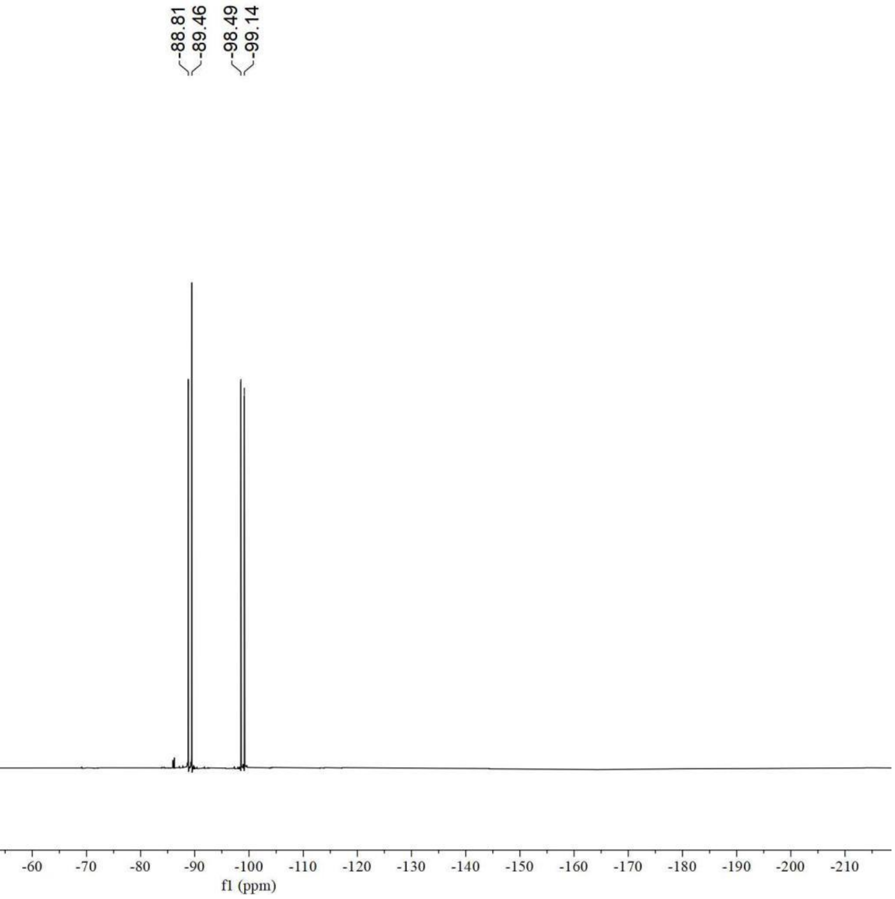

4-(2,2-Difluoro-2-phenylethyl)-9-methoxy-1,2,3,4-tetrahydrobenzo[4,5]imidazo[1,2-*a*]pyridine (3t)

^{1}H NMR (400 MHz, $CDCl_3$)

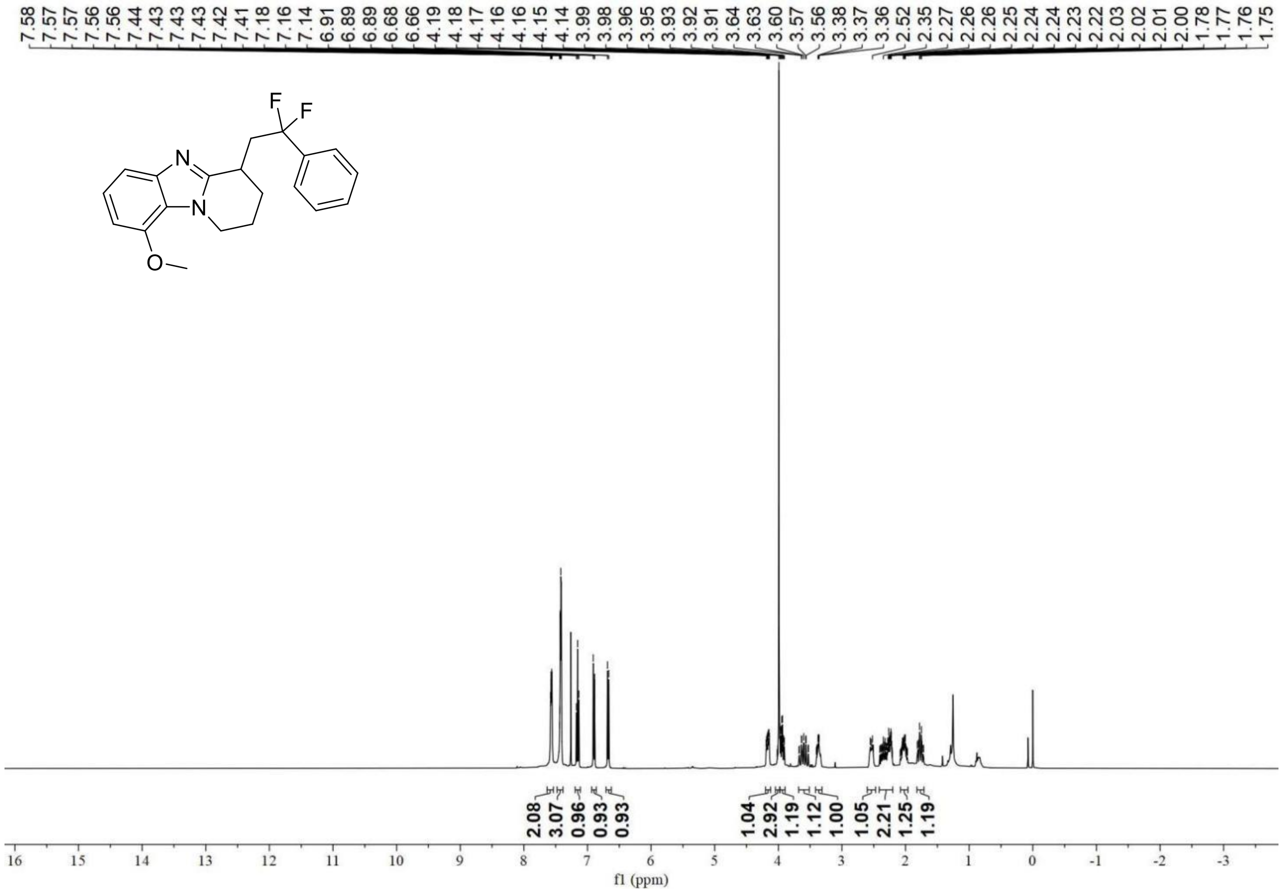

^{13}C NMR (100 MHz, $CDCl_3$)

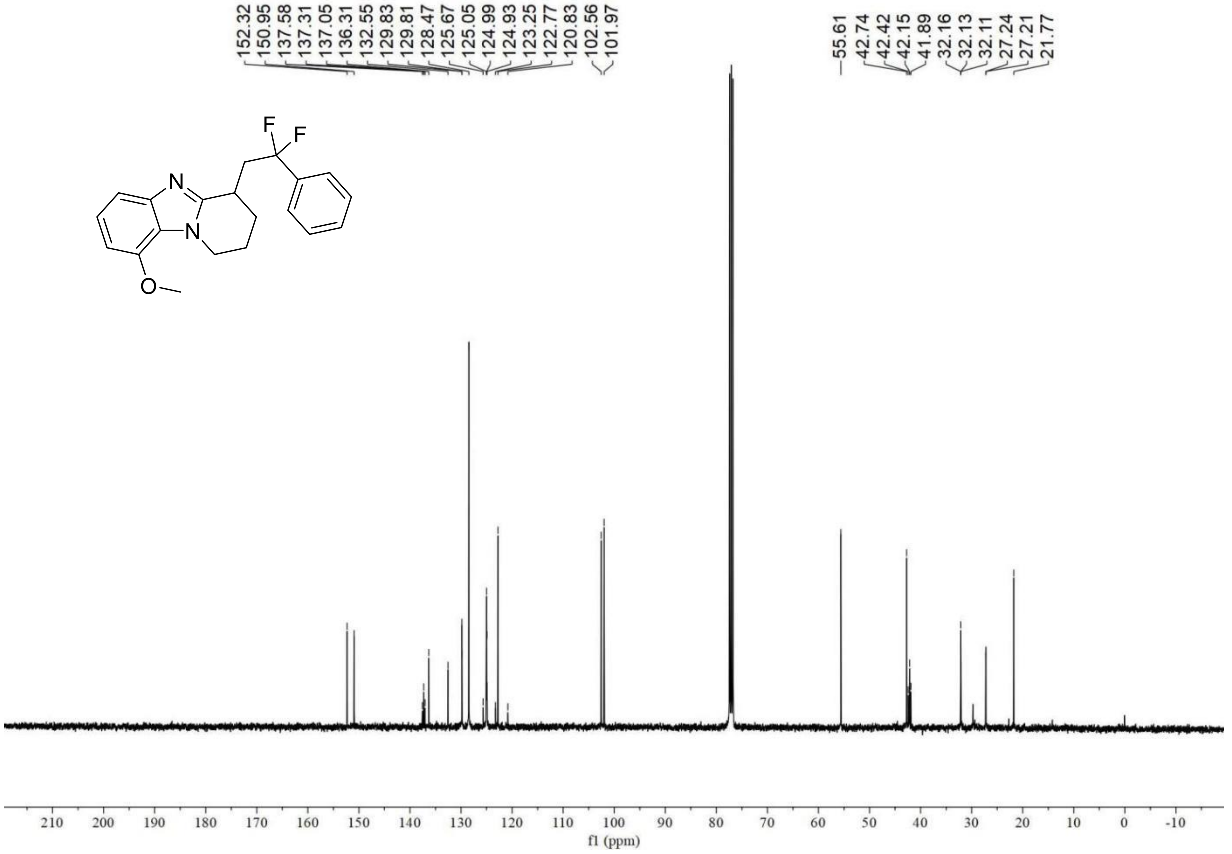

$^{19}F\{H\}$ NMR (376 MHz, $CDCl_3$)

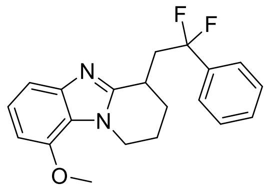

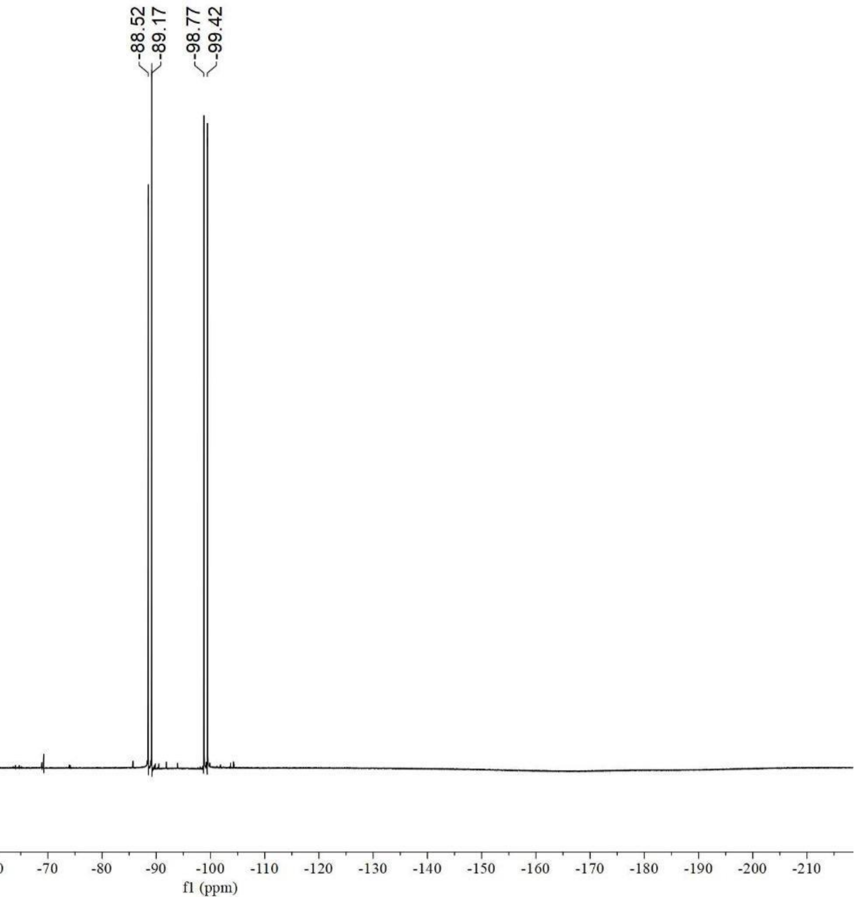

4-(2,2-difluoro-2-phenylethyl)-7,8-difluoro-1,2,3,4-tetrahydrobenzo[4,5]imidazo[1,2-*a*]pyridine (3u)

^{1}H NMR (400 MHz, $CDCl_3$)

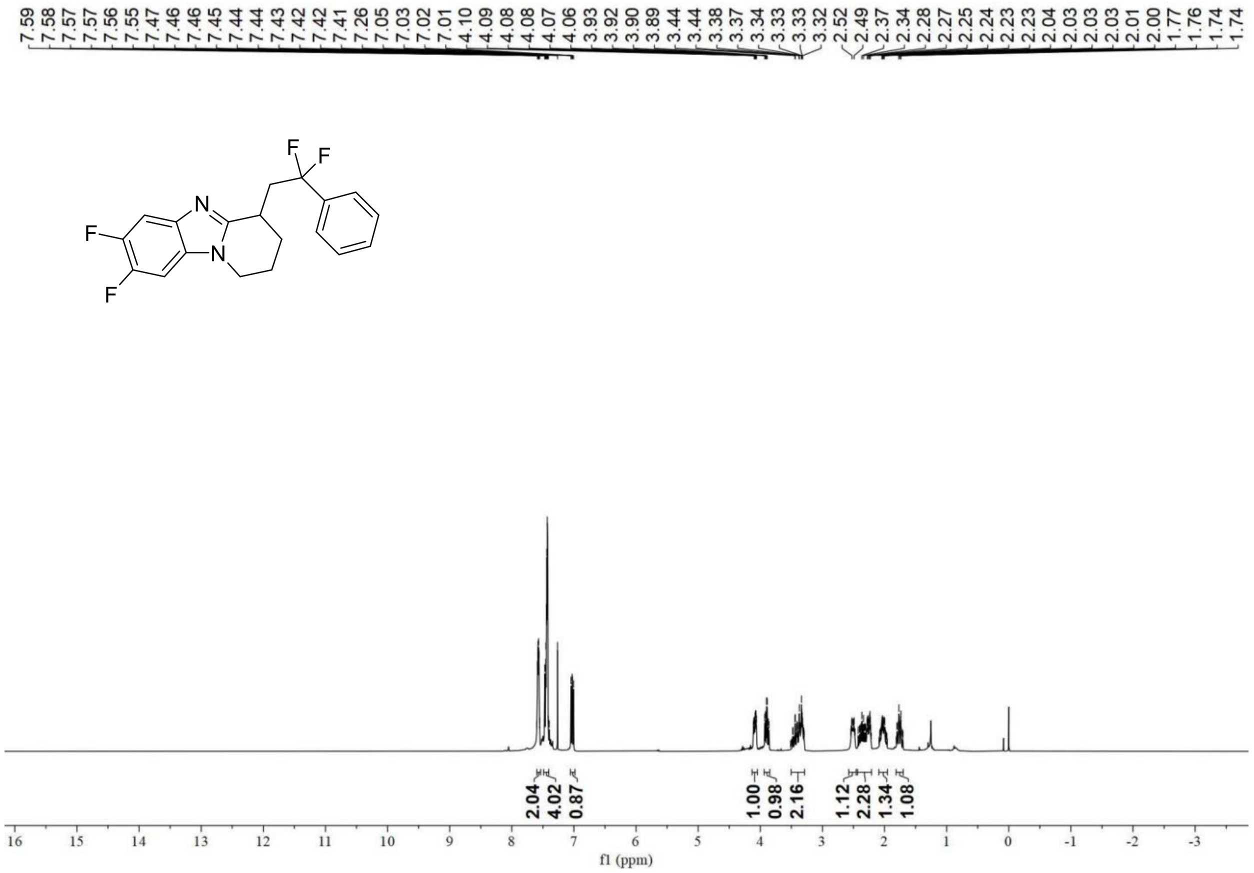

^{13}C NMR (100 MHz, $CDCl_3$)

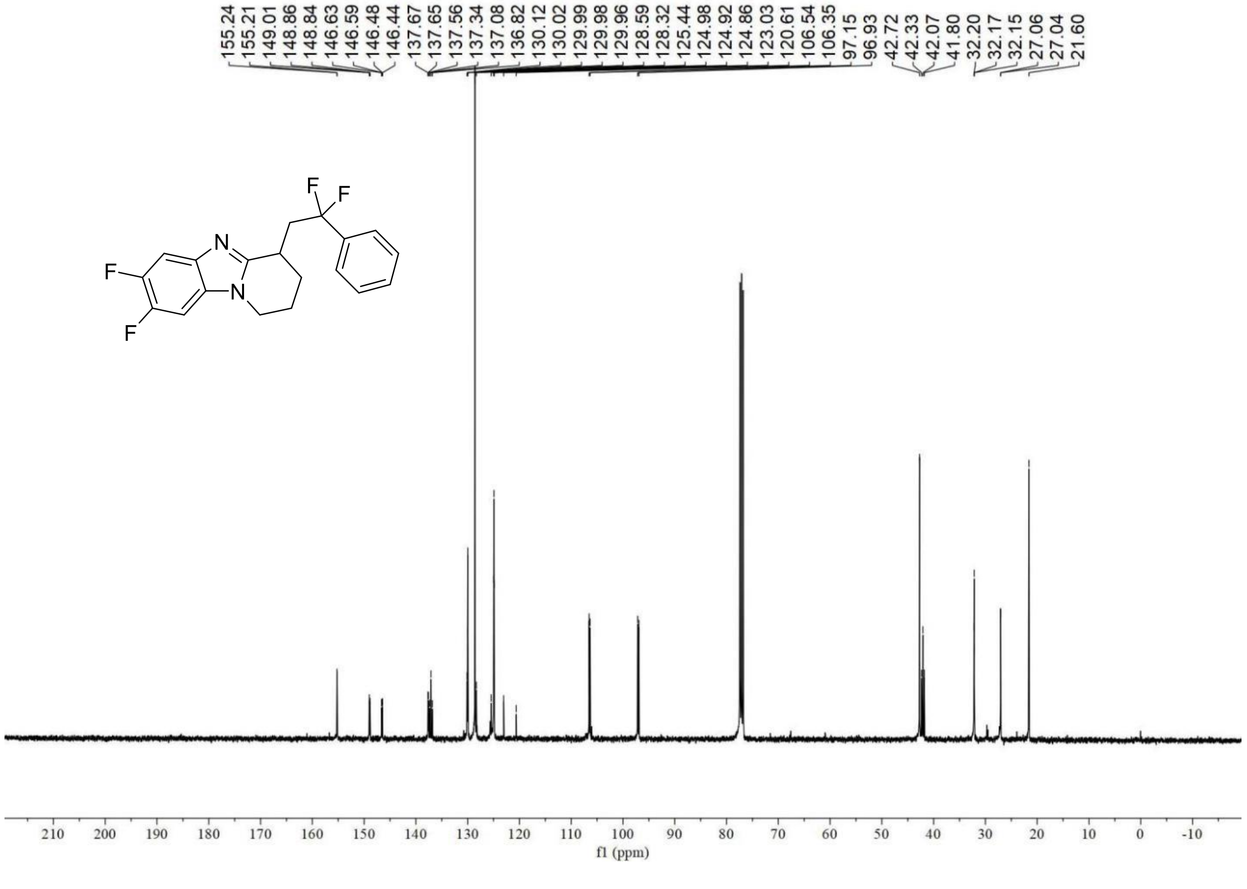

^{19}F｛H｝NMR (376 MHz, $CDCl_3$)

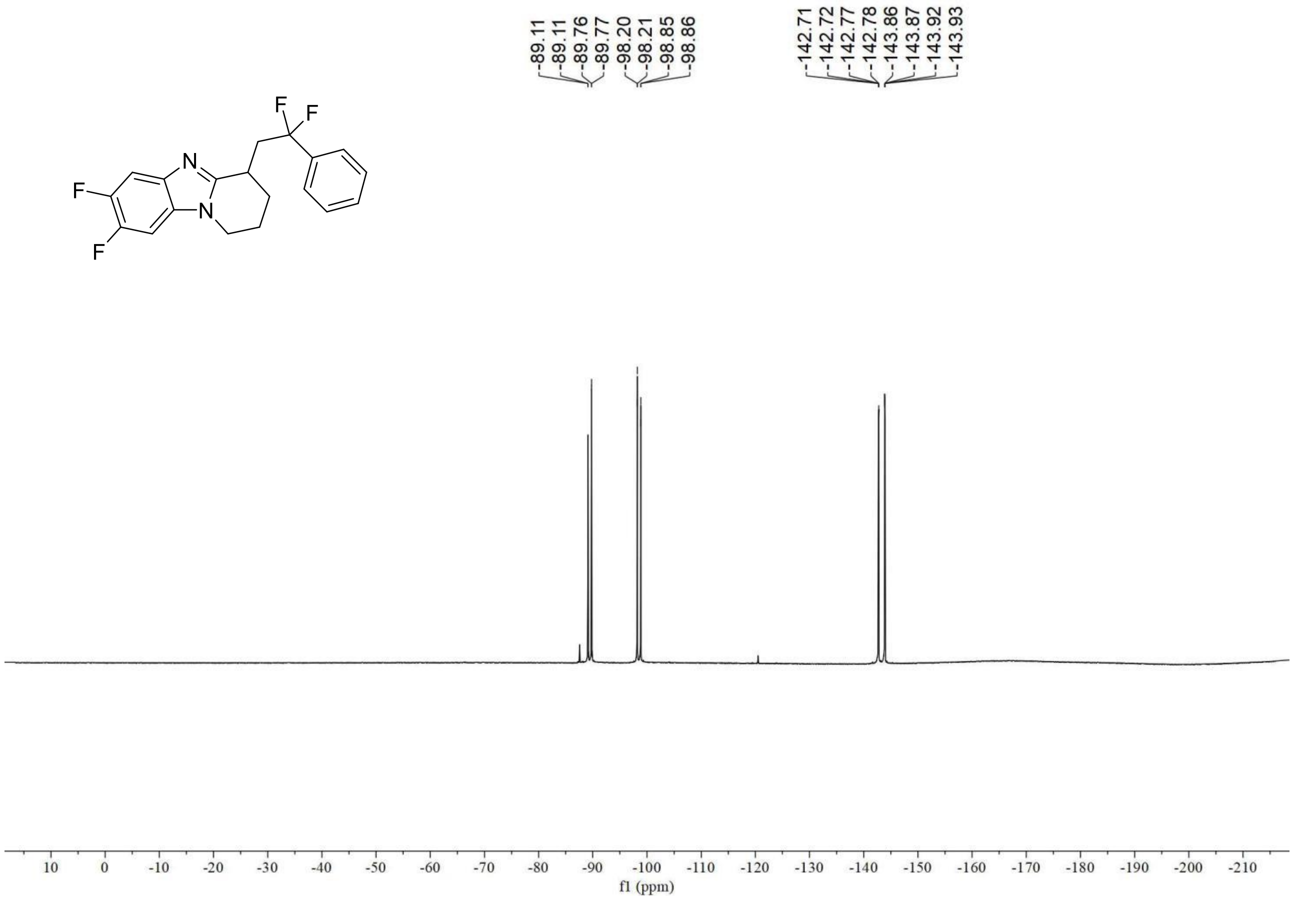

7,8-Dichloro-4-(2,2-difluoro-2-phenylethyl)-1,2,3,4-tetrahydrobenzo[4,5]imidazo[1,2-*a*]pyridine (3v)

^{1}H NMR (400 MHz, $CDCl_3$)

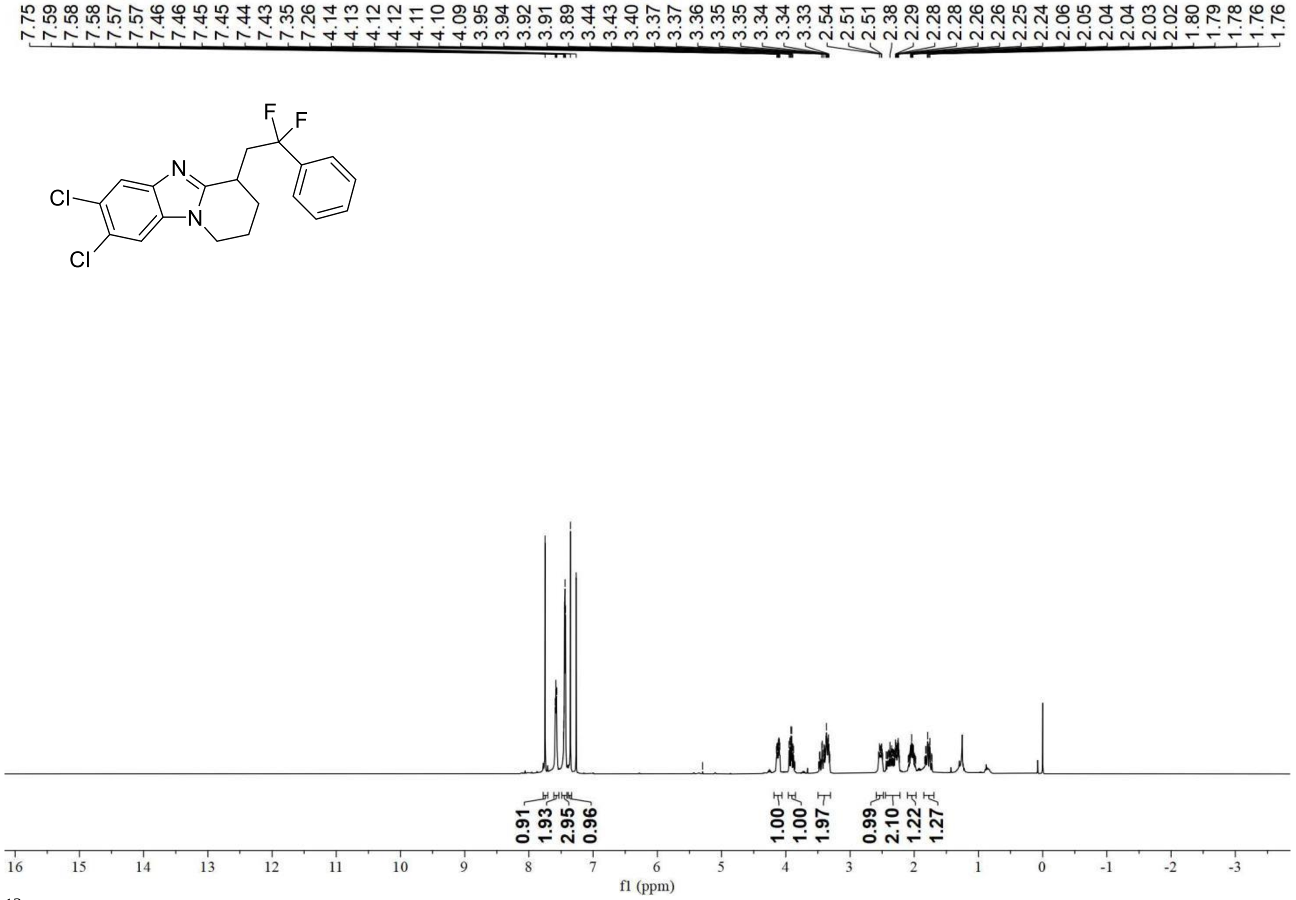

^{13}C NMR (100 MHz, $CDCl_3$)

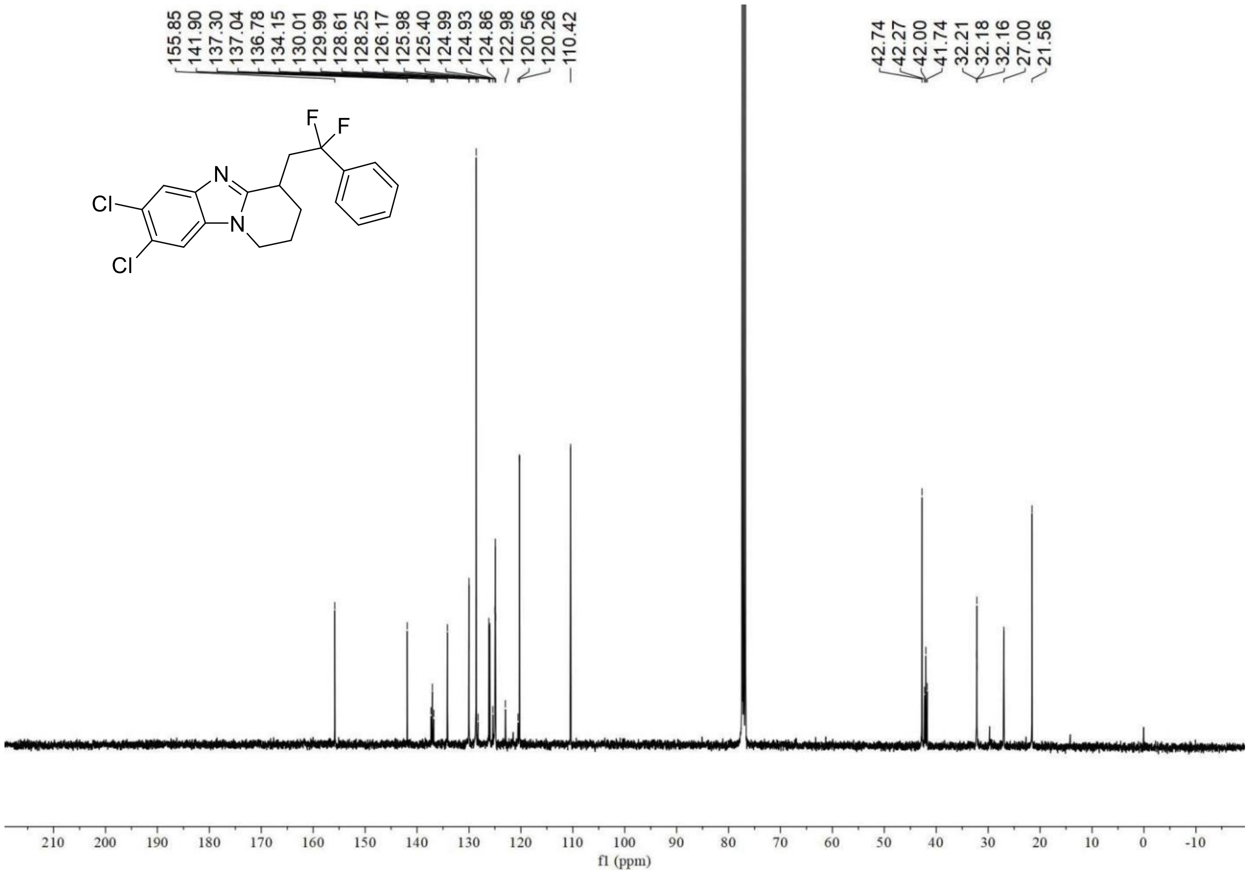

^{19}F {H} NMR (376 MHz, $CDCl_3$)

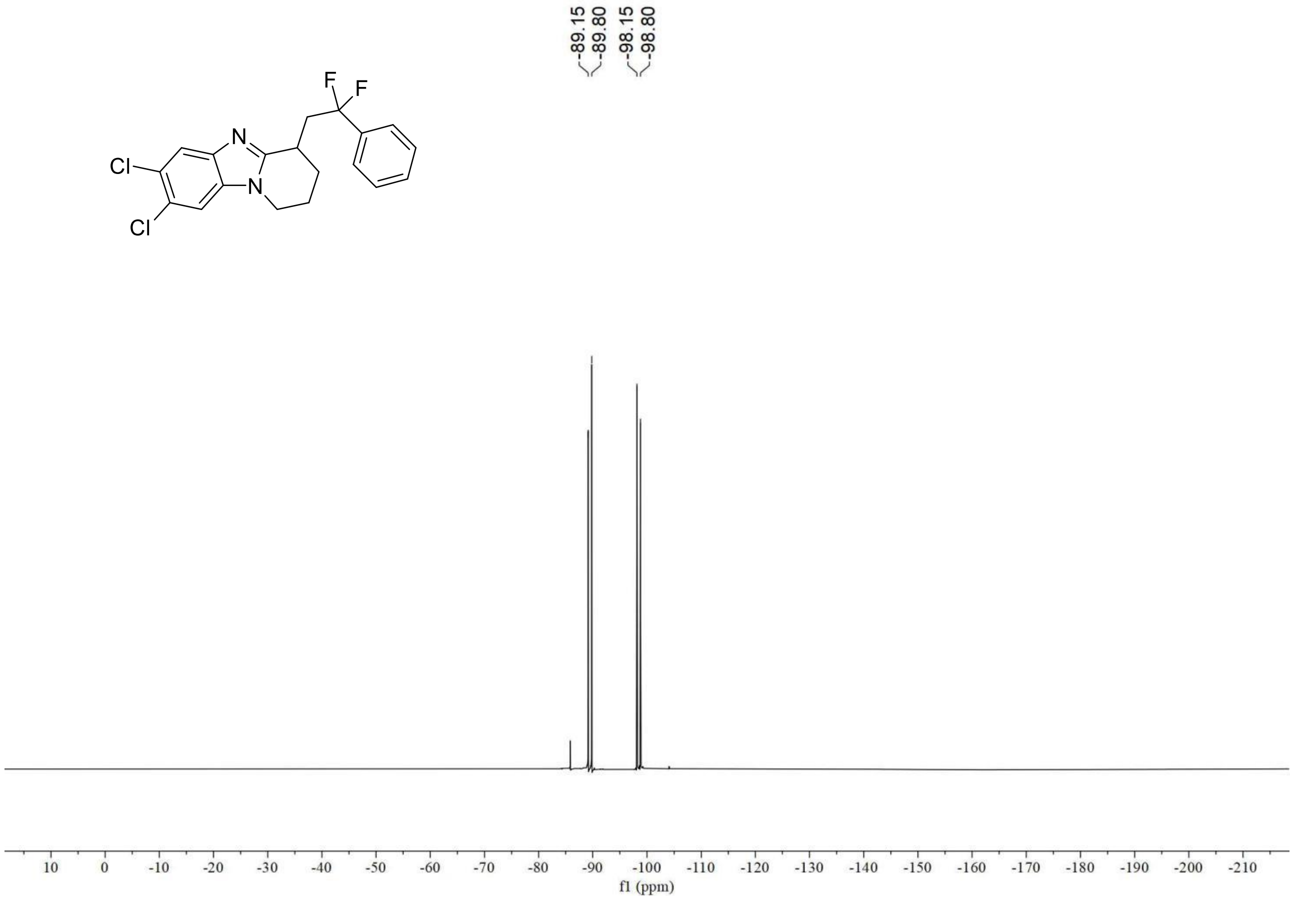

4-(2,2-Difluoro-2-phenylethyl)-7,8-dimethyl-1,2,3,4-tetrahydrobenzo[4,5]imidazo[1,2-*a*]pyridine (3w)

^{1}H NMR (400 MHz, $CDCl_3$)

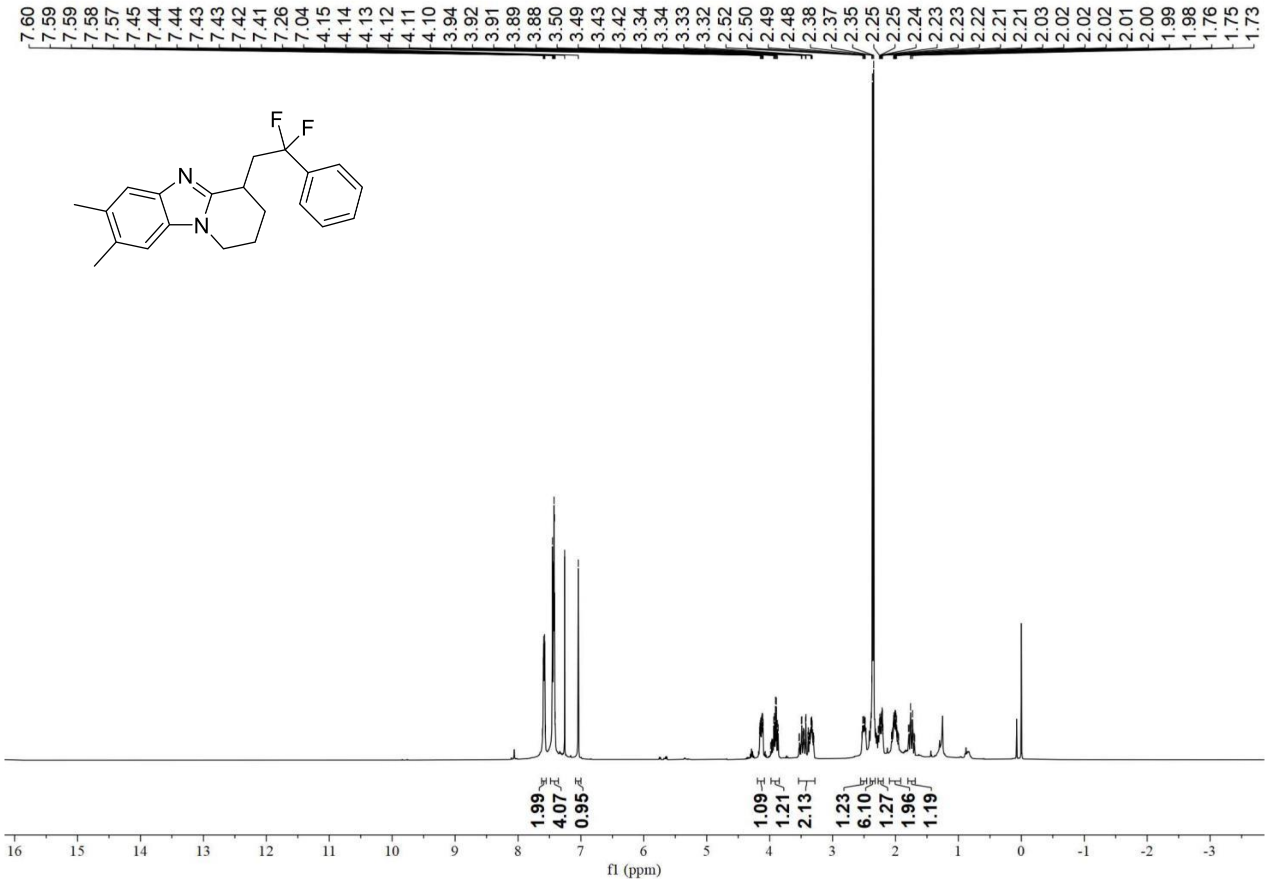

^{13}C NMR (100 MHz, $CDCl_3$)

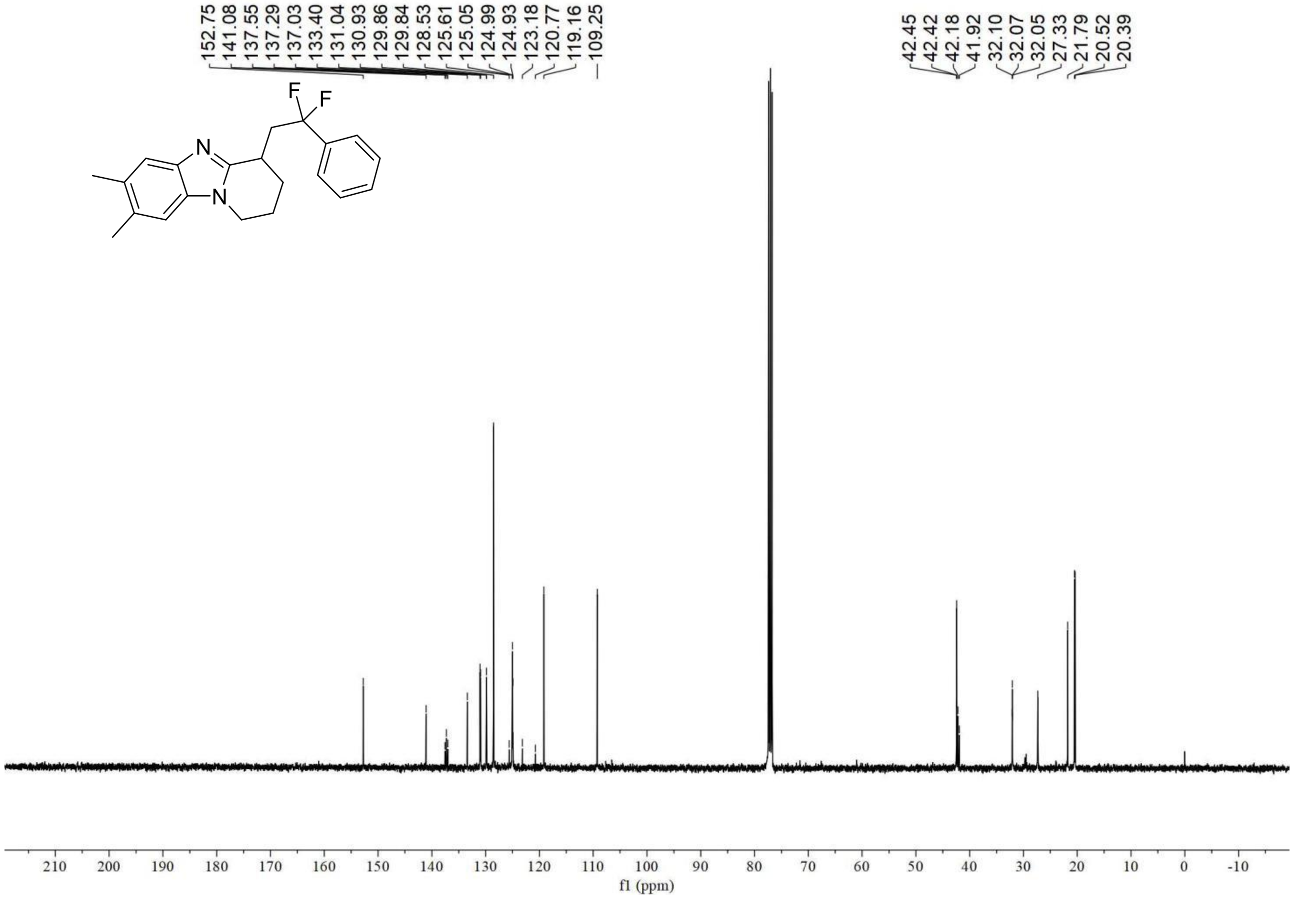

$^{19}F\{H\}$ NMR (376 MHz, $CDCl_3$)

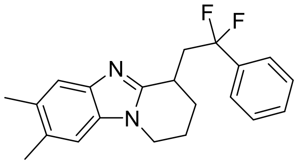

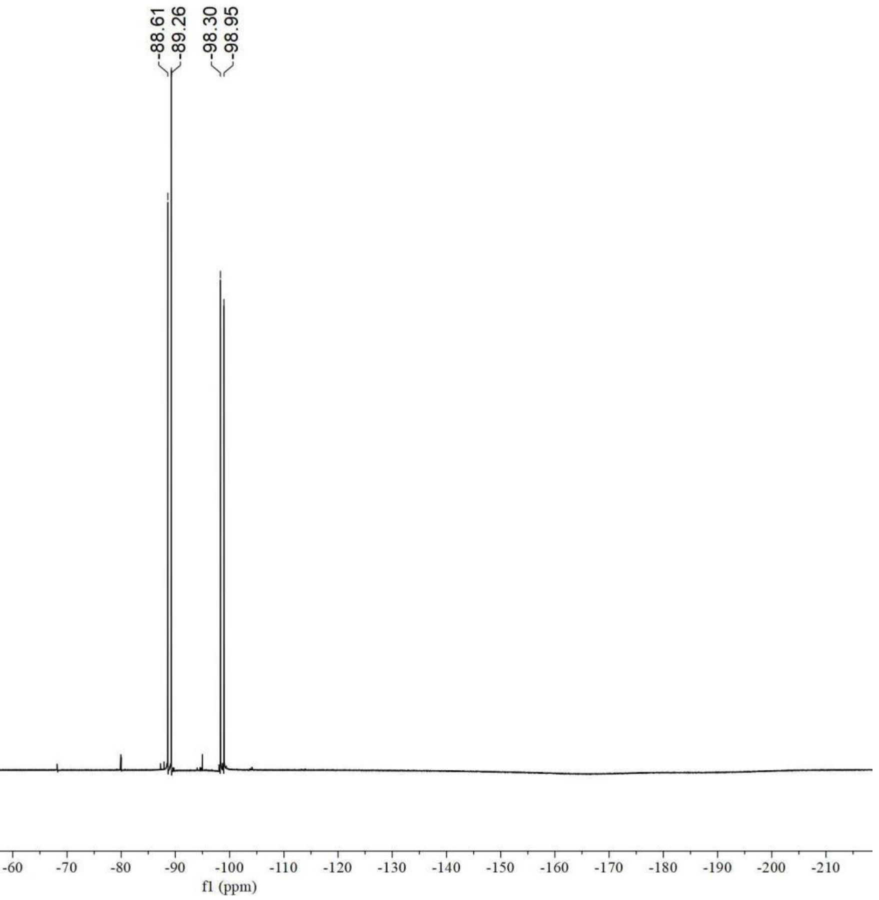

Dimethyl 8-(2,2-difluoro-2-phenylethyl)-5,6,7,8-tetrahydroimidazo[1,2-*a*]pyridine-2,3-dicarboxylate (3x)

^{1}H NMR (400 MHz, $CDCl_3$)

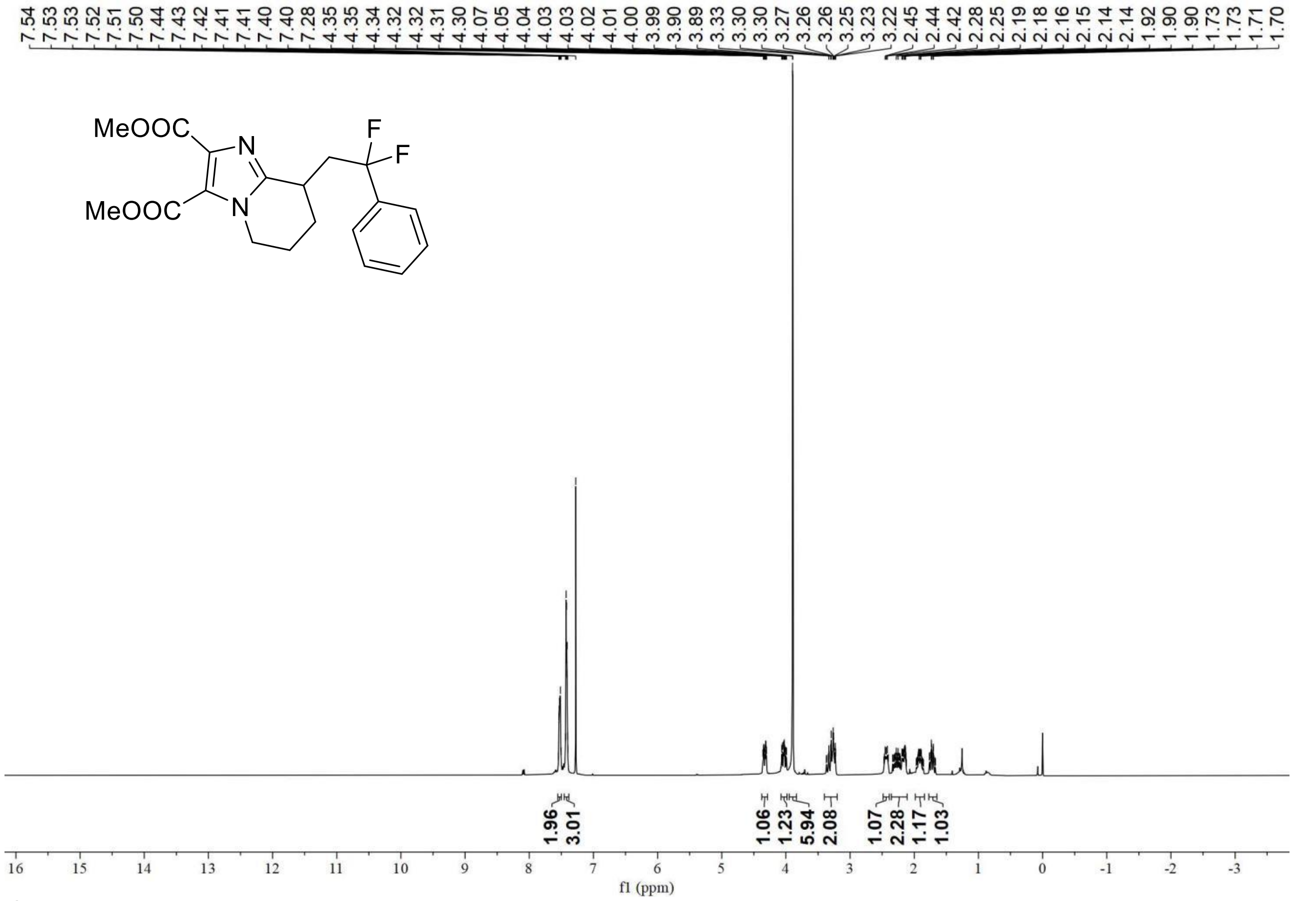

^{13}C NMR (100 MHz, $CDCl_3$)

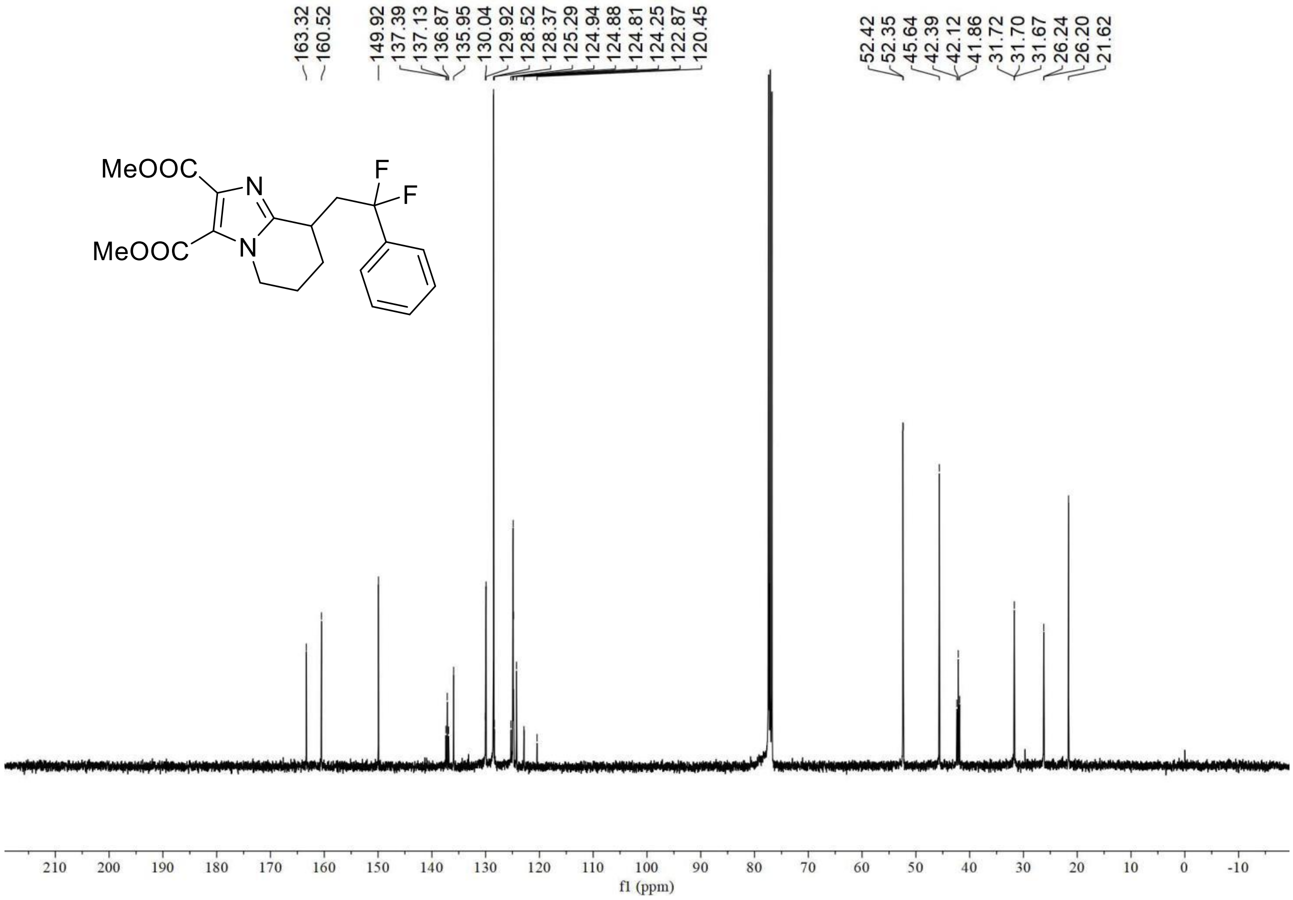

$^{19}F\{H\}$ NMR (376 MHz, $CDCl_3$)

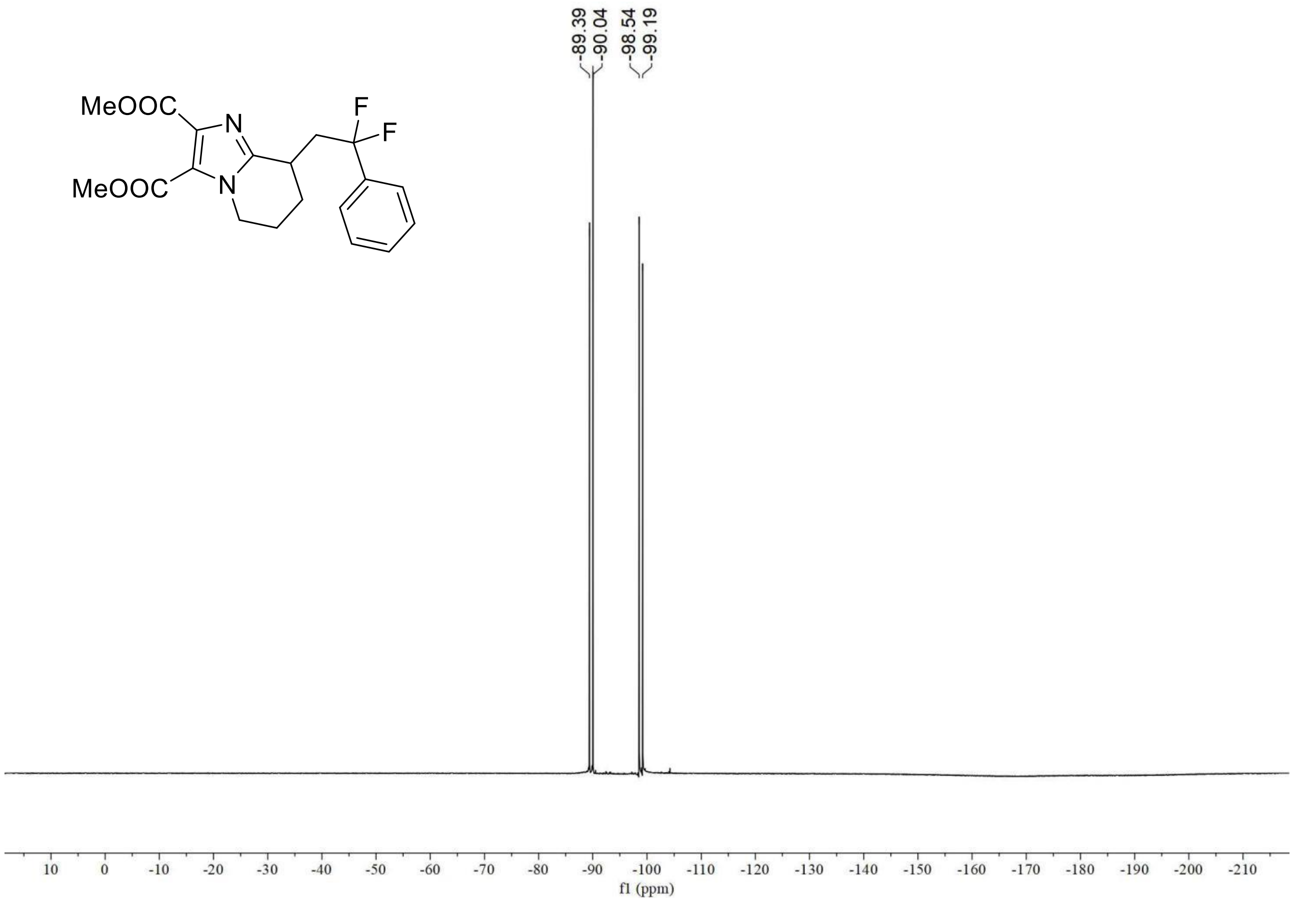

References

[1]. Lin, S.; Deng Y.; Zhong, H.; Mao, L.; Ji, C.; Zhu, X.; Zhang, X.; Yang, B. Visible Light-Induced Radical Cascade Difluoromethylation/Cyclization of Unactivated Alkenes: Access to CF_2H-Substituted Polycyclic Imidazoles. *ACS Omega* **2024**, 9, 26, 28129–28143.

[2]. Huang, P.; Lv, C.; Song, H.; Wang, C.; Du, J.; Li, J.; Sun,B.; Jin, C.; An in situ generated proton initiated aromatic fluoroalkylation via electron donor-acceptor complex photoactivation. *Green Chem*., **2024**,26, 7198-7205.

[3]. Lv, Y.; Dai, J.-Y.; Zhao, Z.-X.; Liu, J.; Li, Z.-W.; Lu, C.-H.; Zhang, Y.-F.; Liu, W.-D.; Li, J.-S. Electrochemical Synthesis of 5-Trifluoroethyl Dihydrobenzimidazo[2,1-a]isoquinolines from Pendent Unactivated Alkenes via Radical Relay. *Tetrahedron Lett*. **2023**, 119, 154410-154414.
